# Primaquine for uncomplicated *Plasmodium vivax* malaria in children younger than 15 years: a systematic review and individual patient data meta-analysis

**DOI:** 10.1016/S2352-4642(24)00210-4

**Published:** 2024-11

**Authors:** Robert J Commons, Megha Rajasekhar, Elizabeth N Allen, Daniel Yilma, Palang Chotsiri, Tesfay Abreha, Ishag Adam, Ghulam Rahim Awab, Bridget E Barber, Larissa W Brasil, Cindy S Chu, Liwang Cui, Peta Edler, Margarete do Socorro M Gomes, Lilia Gonzalez‑Ceron, Matthew J Grigg, Muzamil Mahdi Abdel Hamid, Jimee Hwang, Harin Karunajeewa, Marcus V G Lacerda, Simone Ladeia-Andrade, Toby Leslie, Rhea J Longley, Wuelton Marcelo Monteiro, Ayodhia Pitaloka Pasaribu, Jeanne Rini Poespoprodjo, Caitlin L Richmond, Komal Raj Rijal, Walter R J Taylor, Pham Vinh Thanh, Kamala Thriemer, José Luiz F Vieira, Nicholas J White, Lina M Zuluaga-Idarraga, Lesley J Workman, Joel Tarning, Kasia Stepniewska, Philippe J Guerin, Julie A Simpson, Karen I Barnes, Ric N Price, WorldWide Antimalarial Resistance Network Paediatric Primaquine Vivax Study Group, Bipin Adhikari, Mohammad Shafiul Alam, Nicholas M Anstey, Ashenafi Assefa, J Kevin Baird, Sarah C Boyd, Nguyen H Chau, Nicholas PJ Day, Tamiru Shibiru Degaga, Arjen M Dondorp, Annette Erhart, Marcelo U Ferreira, Prakash Ghimire, Wasif A Khan, Benedikt Ley, Asrat H Mekuria, Ivo Mueller, Mohammad N Naadim, Francois Nosten, David J Price, Sasithon Pukrittayakamee, Mark Rowland, Jetsumon Sattabongkot, Guilherme SuarezKurtz, Inge Sutanto, Lorenz von Seidlein, Timothy William, Charles J Woodrow, Adugna Woyessa

**Affiliations:** a Global Health Division, Menzies School of Health Research and Charles Darwin University, Darwin, NT, Australia; b WorldWide Antimalarial Resistance Network, Asia-Pacific Regional Centre, Melbourne, VIC, Australia; c General and Subspecialty Medicine, Grampians Health Ballarat, Ballarat, VIC, Australia; d Centre for Epidemiology and Biostatistics, Melbourne School of Population and Global Health, The University of Melbourne, Melbourne, VIC, Australia; e Department of Medical Biology, The University of Melbourne, Melbourne, VIC, Australia; f Division of Clinical Pharmacology, Department of Medicine, University of Cape Town, Cape Town, South Africa; g WorldWide Antimalarial Resistance Network Pharmacology Scientific Group, University of Cape Town, Cape Town, South Africa; h Infectious Diseases Data Observatory, Oxford, UK; i Jimma University Clinical Trial Unit, Department of Internal Medicine, Jimma University, Jimma, Ethiopia; j Mahidol Oxford Tropical Medicine Research Unit, Faculty of Tropical Medicine, Mahidol University, Bangkok, Thailand; k Mahidol Vivax Research Unit, Faculty of Tropical Medicine, Mahidol University, Bangkok, Thailand; l Department of Clinical Tropical Medicine, Faculty of Tropical Medicine, Mahidol University, Bangkok, Thailand; m ICAP, Columbia University Mailman School of Public Health, Addis Ababa, Ethiopia; n Department of Obstetrics and Gynecology, College of Medicine, Qassim University, Buraidah, Saudi Arabia; o Nangarhar Medical Faculty, Nangarhar University, Jalalabad, Afghanistan; p QIMR Berghofer Medical Research Institute, Brisbane, QLD, Australia; q Infectious Diseases Society Sabah-Menzies School of Health Research Clinical Research Unit, Kota Kinabalu, Sabah, Malaysia; r Diretoria de Ensino e Pesquisa, Fundação de Medicina Tropical Dr Heitor Vieira Dourado, Manaus, Brazil; s Programa de Pós‑Graduação em Medicina Tropical, Universidade do Estado do Amazonas, Manaus, Brazil; t Shoklo Malaria Research Unit, Mahidol Oxford Tropical Medicine Research Unit, Faculty of Tropical Medicine, Mahidol University, Mae Sot, Thailand; u Centre for Tropical Medicine and Global Health, Nuffield Department of Medicine, University of Oxford, Oxford, UK; v Department of Internal Medicine, Morsani College of Medicine, University of South Florida, Tampa, FL, USA; w Department of Infectious Diseases, The University of Melbourne at the Peter Doherty Institute for Infection and Immunity, Melbourne, VIC, Australia; x Superintendência de Vigilância em Saúde do Estado do Amapá - SVS/AP, Macapá, Amapá, Brazil; y Federal University of aMAPA (Universidade Federal do Amapá - UNIFAP), Macapá, Amapá, Brazil; z Regional Centre for Public Health Research, National Institute for Public Health, Tapachula, Chiapas, Mexico; aa Department of Parasitology and Medical Entomology, Institute of Endemic Diseases, University of Khartoum, Khartoum, Sudan; ab US President's Malaria Initiative, Malaria Branch, US Centers for Disease Control and Prevention, Atlanta, GA, USA; ac Institute for Global Health Sciences, University of California San Francisco, San Francisco, CA, USA; ad Department of Medicine-Western Health, Melbourne Medical School, The University of Melbourne, St Albans, VIC, Australia; ae Fundação de Medicina Tropical Dr Heitor Vieira Dourado, Manaus, Brazil; af Instituto Leônidas & Maria Deane, Fiocruz, Manaus, Brazil; ag University of Texas Medical Branch, Galveston, TX, USA; ah Laboratory of Parasitic Diseases, Oswaldo Cruz Foundation (Fiocruz), Rio de Janeiro, Brazil; ai Global Health and Tropical Medicine, Institute of Hygiene and Tropical Medicine, NOVA University of Lisbon, Lisbon, Portugal; aj Department of Infectious and Tropical Diseases, London School of Hygiene and Tropical Medicine, London, UK; ak HealthNet-TPO, Kabul, Afghanistan; al Population Health and Immunity Division, Walter and Eliza Hall Institute of Medical Research, Melbourne, VIC, Australia; am Universidade do Estado do Amazonas, Manaus, Brazil; an Department of Pediatrics, Medical Faculty, Universitas Sumatera Utara, Medan, North Sumatera, Indonesia; ao Mimika District Hospital, Timika, Indonesia; ap Timika Malaria Research Programme, Papuan Health and Community Development Foundation, Timika, Indonesia; aq Paediatric Research Office, Department of Child Health, Faculty of Medicine, Public Health and Nursing, Universitas Gadjah Mada/Dr Sardjito Hospital, Yogyakarta, Indonesia; ar WorldWide Antimalarial Resistance Network, Oxford, UK; as Central Department of Microbiology, Tribhuvan University, Kirtipur, Nepal; at National Institute of Malariology, Parasitology and Entomology, Hanoi, Vietnam; au Federal University of Pará (Universidade Federal do Pará - UFPA), Belém, Pará, Brazil; av Grupo Malaria, Facultad de Medicina, Universidad de Antioquia, Medellín, Colombia; aw Facultad Nacional de Salud Publica, Universidad de Antioquia, Medellín, Colombia

## Abstract

**Background:**

Primaquine, the only widely available treatment to prevent relapsing *Plasmodium vivax* malaria, is produced as 15 mg tablets, and new paediatric formulations are being developed. To inform the optimal primaquine dosing regimen for children, we aimed to determine the efficacy and safety of different primaquine dose strategies in children younger than 15 years.

**Methods:**

We undertook a systematic review (Jan 1, 2000–July 26, 2024) for *P vivax* efficacy studies with at least one treatment group that was administered primaquine over multiple days, that enrolled children younger than 15 years, that followed up patients for at least 28 days, and that had data available for inclusion by June 30, 2022. Patients were excluded if they were aged 15 years or older, presented with severe malaria, received adjunctive antimalarials within 14 days of diagnosis, commenced primaquine more than 7 days after starting schizontocidal treatment, had a protocol violation in the original study, or were missing data on age, sex, or primaquine dose. Available individual patient data were collated and standardised. To evaluate efficacy, the risk of recurrent *P vivax* parasitaemia between days 7 and 180 was assessed by time-to-event analysis for different total mg/kg primaquine doses (low total dose of ∼3·5 mg/kg and high total dose of ∼7 mg/kg). To evaluate tolerability and safety, the following were assessed by daily mg/kg primaquine dose (low daily dose of ∼0·25 mg/kg, intermediate daily dose of ∼0·5 mg/kg, and high daily dose of ∼1 mg/kg): gastrointestinal symptoms (vomiting, anorexia, or diarrhoea) on days 5–7, haemoglobin decrease of at least 25% to less than 7g/dL (severe haemolysis), absolute change in haemoglobin from day 0 to days 2–3 or days 5–7, and any serious adverse events within 28 days. This study is registered with PROSPERO, CRD42021278085.

**Findings:**

In total, 3514 children from 27 studies and 15 countries were included. The cumulative incidence of recurrence by day 180 was 51·4% (95% CI 47·0–55·9) following treatment without primaquine, 16·0% (12·4–20·3) following a low total dose of primaquine, and 10·2% (8·4–12·3) following a high total dose of primaquine. The hazard of recurrent *P vivax* parasitaemia in children younger than 15 years was reduced following primaquine at low total doses (adjusted hazard ratio [HR] 0·17, 95% CI 0·11–0·25) and high total doses (0·09, 0·07–0·12), compared with no primaquine. In 525 children younger than 5 years, the relative rates of recurrence were also reduced, with an adjusted HR of 0·33 (95% CI 0·18–0·59) for a low total dose and 0·13 (0·08–0·21) for a high total dose of primaquine compared with no primaquine. The rate of recurrence following a high total dose was reduced compared with a low dose in children younger than 15 years (adjusted HR 0·54, 95% CI 0·35–0·85) and children younger than 5 years (0·41, 0·21–0·78). Compared with no primaquine, children treated with any dose of primaquine had a greater risk of gastrointestinal symptoms on days 5–7 after adjustment for confounders, with adjusted risks of 3·9% (95% CI 0–8·6) in children not treated with primaquine, 9·2% (0–18·7) with a low daily dose of primaquine, 6·8% (1·7–12·0) with an intermediate daily dose of primaquine, and 9·6% (4·8–14·3) with a high daily dose of primaquine. In children with 30% or higher glucose-6-phosphate dehydrogenase (G6PD) activity, there were few episodes of severe haemolysis following no primaquine (0·4%, 95% CI 0·1–1·5), a low daily dose (0·0%, 0·0–1·6), an intermediate daily dose (0·5%, 0·1–1·4), or a high daily dose (0·7%, 0·2–1·9). Of 15 possibly drug-related serious adverse events in children, two occurred following a low, four following an intermediate, and nine following a high daily dose of primaquine.

**Interpretation:**

A high total dose of primaquine was highly efficacious in reducing recurrent *P vivax* parasitaemia in children compared with a low dose, particularly in children younger than 5 years. In children treated with high and intermediate daily primaquine doses compared with low daily doses, there was no increase in gastrointestinal symptoms or haemolysis (in children with 30% or higher G6PD activity), but there were more serious adverse events.

**Funding:**

Medicines for Malaria Venture, Bill & Melinda Gates Foundation, and Australian National Health and Medical Research Council.

## Introduction

*Plasmodium vivax* remains endemic in 49 countries and is becoming the predominant malaria species outside Africa.^1^ Between 5 million and 14 million cases of *P vivax* malaria occur each year. *P vivax* and *Plasmodium ovale* are the only major malaria parasites infecting humans that have the ability to form liver stages (hypnozoites) that can lie dormant for weeks to months after the initial infection, before activating to cause recurrent malaria episodes (relapses).

Primaquine, an 8-aminoquinoline, is the only WHO-recommended antimalarial that kills hypnozoites.^2^ The greatest burden of *P vivax* malaria is in young children, who are particularly vulnerable to cumulative anaemia associated with recurrent episodes of malaria.^3^ Despite the need for reliable treatment in this population, no WHO pre-qualified paediatric primaquine formulations are available currently. The reliance on adult tablet formulations results in inaccurate paediatric dosing, potentially resulting in a greater risk of adverse events and lower efficacy. The need for a paediatric primaquine formulation has been recognised by WHO.^4^

At the correct dose, primaquine is highly efficacious against hypnozoites but can cause severe drug-induced haemolysis in individuals with glucose-6-phosphate dehydrogenase (G6PD) deficiency. The risk of haemolysis depends on the duration and dose of primaquine.^5^ At high daily doses, primaquine can also induce gastrointestinal symptoms, potentially reducing adherence.

Since its introduction into clinical practice in the 1950s, clinical trials have documented the safety and efficacy of primaquine, but data have not been collated to define the safety, tolerability, and efficacy of different primaquine regimens in children. To inform the development of optimal weight-based primaquine dosing regimens for new, age-appropriate formulations of primaquine in children, we undertook a systematic review and individual patient data meta-analysis to determine the efficacy and safety of different primaquine dose strategies in children younger than 15 years.

## Methods

### Search strategy and selection criteria

For this systematic review and individual patient data meta-analysis, we searched MEDLINE (PubMed), Web of Science, Embase, Scopus, and Cochrane Central for prospective antimalarial efficacy studies of uncomplicated *P vivax* published in any language between Jan 1, 2000, and July 26, 2024, according to the PRISMA of Individual Patient Data statement (appendix pp 3−6). This systematic review was adapted from a previous review to include studies that followed up patients with *P vivax* malaria actively for at least 28 days, included at least one patient aged younger than 15 years, and included a treatment group with daily primaquine given over multiple days where primaquine was co-administered with a schizontocidal antimalarial and commenced within 7 days of starting a schizontocidal antimalarial (appendix p 7).^6^

The review process was undertaken by two independent reviewers (RJC and RNP), with discrepancies resolved by discussion. Following identification of eligible studies published before June 30, 2022, study investigators were contacted and invited to share individual patient data, in addition to data from any eligible but unpublished studies. Data only available after June 30, 2022, were not collated due to the time needed for data transfer and curation. Shared data were de-identified and harmonised in the WorldWide Antimalarial Resistance Network (WWARN) secure repository using standardised methodology.^7^, ^8^ Patients were excluded if they were aged 15 years or older, presented with severe malaria, received adjunctive antimalarials within 14 days of diagnosis, received an alternative hypnozoiticidal agent to primaquine, commenced primaquine more than 7 days after starting schizontocidal treatment, had a protocol violation in the original study, or were missing data on age, sex, or primaquine dose. Additional study-level and patient-level inclusion and exclusion criteria were used for the efficacy, tolerability, haematology, and adverse event meta-analyses (appendix p 7). The study was registered on PROSPERO (CRD42021278085). Shared data were obtained according to ethical approvals from the country of origin and original study. The data are pseudonymised and cannot be linked to individuals. As such, this analysis did not require additional ethical approval according to the guidelines of the Oxford Central University Research Ethics Committee. We involved people with lived experience of vivax malaria in the study design, analysis, and writing.

**Research in context**

**Evidence before this study**

We identified randomised controlled trials that enrolled children and compared low and high total doses of primaquine regimens for *Plasmodium vivax* mono-infection, published in any language, from Jan 1, 1960, to July 26, 2024, in MEDLINE, Web of Science, Embase, and Cochrane Central, using the terms “vivax” and “primaquine”. We identified three trials, with none reporting results specifically for children.

**Added value of this study**

Our individual patient data meta-analysis included 3514 children younger than 15 years with *Plasmodium vivax* malaria enrolled into 27 studies to assess the efficacy, tolerability, and safety of different primaquine doses. A high total dose of primaquine (7 mg/kg) was 46% more efficacious than a low total dose (3·5 mg/kg) at preventing recurrent malaria, with the greatest benefit in children younger than 5 years. High daily doses and intermediate daily doses compared with low daily doses of primaquine were not associated with increases in gastrointestinal symptoms or haemolysis in children with 30% or higher glucose-6-phosphate dehydrogenase (G6PD) activity; however, more serious adverse events were reported with high daily doses (1 mg/kg).

**Implications of all the available evidence**

In children, a high total dose of primaquine is needed to achieve radical cure of *P vivax* and reduce the risk of recurrences reliably. In children with 30% or higher G6PD activity, primaquine doses up to 0·5 mg/kg per day were safe and well tolerated.

### Definitions

The total dose of primaquine (mg/kg) was used as the exposure of interest for the efficacy analyses, whereas the daily dose of primaquine (mg/kg per day) was the exposure of interest for the tolerability and safety analyses.

The total dose of primaquine was assessed as a categorical variable (a very low dose was defined as <2 mg/kg, a low dose as 2 mg/kg to <5 mg/kg [equivalent to the standard total 3·5 mg/kg dose], and a high dose as ≥5 mg/kg [equivalent to the standard 7 mg/kg dose])^9^ to enable comparison of low-dose and high-dose regimens. A continuous variable for total dose was used to investigate the effect of small dose changes. Intermittent primaquine was defined as weekly or monthly dosing. The daily dose of primaquine was defined as a low dose if it was less than 0·375 mg/kg, an intermediate dose if it was 0·375 mg/kg or higher and less than 0·75 mg/kg, and a high dose if it was 0·75 mg/kg or higher, reflecting the standard 0·25 mg/kg per day, 0·5 mg/kg per day, and 1 mg/kg per day doses, respectively.

The duration of the primaquine regimen was classified as the duration of treatment in days. 7-day and 14-day regimens were compared in patients prescribed a similar total dose of primaquine. Children were categorised by age into younger than 1 year, 1 year to younger than 5 years, and 5 years to younger than 15 years. Only a few studies shared individual patient data on race or ethnicity, and these data could not be pooled due to the global nature of the analysis. Additional definitions are described in the appendix (p 8).

The primary efficacy outcome was the cumulative incidence of recurrent *P vivax* parasitaemia on microscopy between days 7 and 180. The primary gastrointestinal tolerability outcome was a composite endpoint indicating the presence on symptom checklists of vomiting, anorexia, or diarrhoea on days 5–7 after enrolment. The secondary gastrointestinal tolerability outcomes were the presence of the composite endpoint on days 1–2 and vomiting within 1 h of primaquine dosing (acute vomiting). The primary haematology outcomes were a haemoglobin measurement below 7 g/dL on days 1–13 that also represented a 25% or greater decrease in haemoglobin from day 0 to the day of lowest haemoglobin between days 1 and 13 (severe haemolysis); the maximum absolute change in haemoglobin between day 0 and the lowest measured haemoglobin on day 2 or 3; and the maximum absolute change in haemoglobin between day 0 and the lowest measured haemoglobin on days 5–7. The secondary haematology outcomes were a decrease of more than 5 g/dL in haemoglobin between day 0 and the minimum measurement on days 1–13, development of anaemia by day 2–3 or separately by days 5–7, and blood transfusion. Haemoglobin could be measured as haemoglobin or haematocrit by complete blood count or point-of-care test, with haematocrit converted to haemoglobin using the formula by Lee and colleagues.^10^

The primary adverse event outcome was any serious adverse event within 28 days of first primaquine administration. The secondary adverse event outcomes were any adverse events within 28 days of first primaquine administration and adverse events of special interest within 28 days of first primaquine administration including haemoglobinuria, anaemia, methaemoglobinaemia, vomiting, anorexia, diarrhoea, and abdominal pain.

### Data analysis

Included studies were assessed for risk of bias using the Cochrane Risk of Bias 2 tool^11^ for randomised controlled trials and the Joanna Briggs Institute Case Series tool^12^ for single-arm studies. To assess for inclusion bias, the baseline characteristics of included studies were compared with studies that were eligible but not included.

For the efficacy analyses, Kaplan–Meier analysis was used to calculate cumulative incidence of recurrent parasitaemia between day 7 and day 180, presented by primaquine treatment group: no primaquine, low total dose, high total dose, and intermittent primaquine.

Cox regression analysis was used to estimate the association between primaquine use and dose in children and time-to-first *P vivax* recurrence between days 7 and 180. The analysis was restricted to children younger than 15 years treated with daily primaquine or no primaquine. The regression model controlled for age, sex, and baseline parasitaemia, as identified from a causal diagram (appendix p 12) with shared frailty (assumed gamma distribution) for study site. Similar but separate multivariable Cox regression analyses were undertaken to investigate the association between time-to-first recurrence and expected primaquine duration in patients treated with a low total dose of primaquine (∼3·5 mg/kg) and a high total dose of primaquine (∼7 mg/kg). Subgroup analyses were undertaken by relapse periodicity region. Sensitivity analyses were undertaken restricting the outcome to recurrences between days 28 and 180, restricting the analysis to patients for whom the actual dose of primaquine administered was known, restricting the analysis to patients treated with chloroquine, restricting the analysis to studies with at least 180 days of follow-up, restricting the analysis to studies with treatment groups comparing primaquine to no primaquine, undertaking the analysis without shared frailty for study site and by removal of one study site at a time.

A restricted cubic spline model was used to investigate the effect of the continuous mg/kg dose of primaquine on the predicted rate of recurrence between days 7 and day 180 in children. This analysis was repeated post hoc, excluding studies from Melanesia due to a substantially higher risk of recurrence in this region than at other sites. Further details are described in the appendix (p 9).

The risk of gastrointestinal intolerance (and 95% CIs) on days 5–7 was calculated from the number of patients reporting the composite outcome as a percentage of the total number of patients asked about vomiting, anorexia, or diarrhoea on prespecified symptom checklists, specifically on any of days 5–7. The association between primaquine daily dose and the presence of the composite outcome on days 5–7 was estimated using generalised estimating equations (modified Poisson) analysis with a log link, cluster-robust SEs by study site, exchangeable correlation structure, and adjusting for age, sex (male or female), and baseline parasitaemia, as identified by a causal diagram. The effect of co-administering primaquine with food could not be assessed due to absence of data. Sensitivity analyses were undertaken restricted to patients asked about each of vomiting, anorexia, and diarrhoea, restricted to studies that compared a primaquine group with a group without primaquine, restricted to chloroquine or dihydroartemisinin−piperaquine as a schizontocidal treatment, by adjusting for reporting of symptoms on day 0, and after removal of one study at a time. Additional details are described in the appendix (p 10).

Severe haemolysis was defined if the lowest haemoglobin concentration by day 13 was below 7 g/dL and there was a 25% or greater decrease from baseline.^13^ The number and proportion of severe haemolytic events are presented for patients with G6PD activity of 30% or higher, by primaquine daily dose. The absolute reduction in haemoglobin was calculated as the difference in haemoglobin between day 0 and the minimum recorded haemoglobin on days 2–3 or days 5–7, separately. The relationship between haemoglobin change and primaquine daily dose category was modelled using linear mixed-effects models adjusting for age, sex, baseline parasitaemia, and day 0 haemoglobin and normally distributed random intercepts by study site. In a post-hoc analysis, the risk of anaemia by primaquine daily dose category was estimated using generalised estimating equations modified Poisson models. Additional details are described in the appendix (p 11).

The percentage of patients in whom serious adverse events, adverse events, and adverse events of special interest occurred within 28 days of the first dose of primaquine are presented by age group and daily primaquine dose. Additional details are described in the appendix (p 11). Analyses were undertaken in R (version 4.1.3) and Stata (version 17) according to an a priori statistical analysis plan.^14^

### Role of the funding source

The funders of the study reviewed the study protocol and made suggestions that were independently assessed by the authors. They had no role in data collection, data analysis, data interpretation, or writing of the manuscript. The funders reviewed the manuscript before submission and made suggestions that were independently assessed by the authors.

## Results

**Figure 1:**
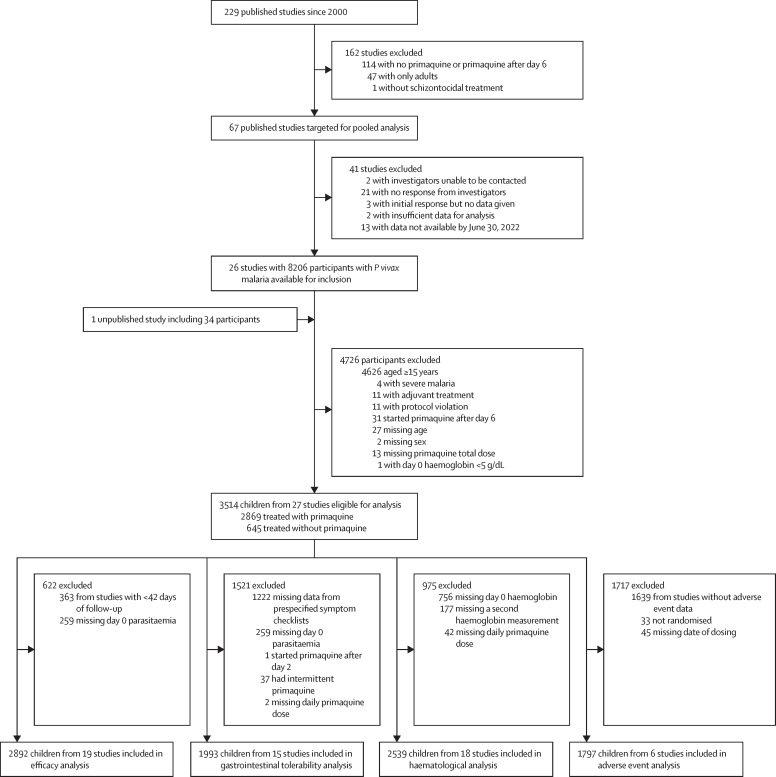
Study flow diagram *P vivax*=*Plasmodium vivax*.

Of 229 *P vivax* efficacy studies published between Jan 1, 2000, and July 26, 2024, 67 were eligible for inclusion in the analysis. Individual data were available from 26 (38·8%) plus one additional unpublished study. Following exclusion of 4726 of the 8240 available participants, 3514 children younger than 15 years (including 628 [17·9%] aged younger than 5 years) from 27 studies (14 randomised controlled trials^15^, ^16^, ^17^, ^18^, ^19^, ^20^, ^21^, ^22^, ^23^, ^24^, ^25^, ^26^, ^27^ and 13 observational studies^28^, ^29^, ^30^, ^31^, ^32^, ^33^, ^34^, ^35^, ^36^, ^37^, ^38^, ^39^, ^40^) in 15 countries were included in the analyses (figure 1; appendix pp 13–21). 24 studies had low or unclear risk of bias (appendix pp 22−23). No included studies recruited children younger than 8 months. Overall, studies included in the analysis recruited patients more recently and followed up patients for longer than those that were eligible but not included (appendix p 24). The majority of the 27 studies were undertaken in the Asia-Pacific region (18 [66·7%]), with seven (25·9%) studies done in the Americas and three (11·1%) done in Africa (one study was multiregional).

Efficacy data were available for 2892 children younger than 15 years from 19 studies^15^, ^17^, ^18^, ^19^, ^20^, ^21^, ^22^, ^23^, ^24^, ^25^, ^26^, ^27^, ^29^, ^33^, ^35^, ^36^, ^38^, ^39^ in 13 countries (538 [18·6%] were aged younger than 5 years). In total, 577 (20·0%) patients were treated without primaquine, 2278 (78·8%) with primaquine administered daily, and 37 (1·3%) with primaquine administered intermittently. Of the 2278 patients administered a daily primaquine regimen, 1043 (45·8%) had a low total dose and 1207 (53·0%) had a high total dose (appendix pp 25–28). 28 (1·0%) patients treated with a very low dose of primaquine, and 37 (1·3%) patients treated with intermittent primaquine were not included in the efficacy analyses. 11 patients with mixed *Plasmodium falciparum* and *P vivax* infection were included in the analysis. Patients treated with low-dose primaquine had lower baseline parasitaemia, were more likely to come from the Americas, and were more likely to have received partially supervised primaquine than those treated with other doses of primaquine or without primaquine (appendix p 25). More patients treated with high-dose primaquine were co-treated with dihydroartemisinin–piperaquine than those with lower doses (appendix p 25).

The cumulative incidence of recurrence by day 180 was 51·4% (95% CI 47·0–55·9) following treatment without primaquine, 16·0% (12·4–20·3) following low-dose primaquine, and 10·2% (8·4–12·3) following high-dose primaquine (appendix p 29). There was marked heterogeneity in cumulative incidence of recurrence between studies (appendix pp 30−31).

**Figure 2:**
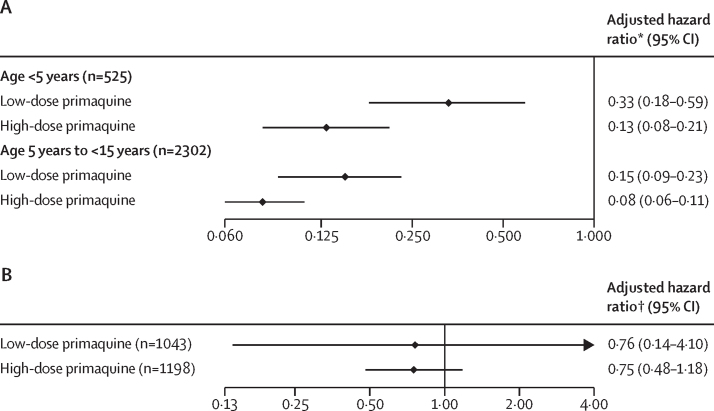
Adjusted hazard ratio for recurrent *P vivax* parasitaemia between day 7 and day 180 for total dose of primaquine by age (A) and 14-day *vs* 7-day primaquine stratified by total dose in children younger than 15 years (B) A low total dose of primaquine was about 3·5 mg/kg, and a high total dose of primaquine was about 7 mg/kg. Cox proportional hazards models adjusting for age, sex (male or female), and (log_10_) baseline parasite density, with shared frailty by study site. *P vivax*=*Plasmodium vivax*. *Adjusted hazard ratio relative to no primaquine. †Adjusted hazard ratio relative to 7 days of primaquine.

After controlling for confounders, the rate of recurrence in 2827 children younger than 15 years between days 7 and 180 was lower following both low-dose primaquine (adjusted hazard ratio [HR] 0·17, 95% CI 0·11–0·25) and high-dose primaquine (adjusted HR 0·09, 0·07–0·12) compared with no primaquine. The corresponding adjusted HRs for recurrence in 525 children younger than 5 years were 0·33 (95% CI 0·18–0·59) for low-dose primaquine and 0·13 (0·08–0·21) for high-dose primaquine, compared with no primaquine (figure 2). The adjusted HR for recurrence following a high total dose of primaquine compared with low-dose primaquine was 0·54 (95% CI 0·35–0·85) in all children and 0·41 (0·21–0·78) in children younger than 5 years. These findings were consistent in sensitivity and subgroup analyses (appendix pp 32–34). The effect of small changes in total primaquine dose are presented in the appendix (pp 34–35).

The rate of recurrence varied with primaquine duration; however, there was substantial uncertainty in the estimated effect. In children treated with a low total dose, the adjusted HR was 0·76 (95% CI 0·14–4·10) for 14-day versus 7-day regimens (76 recurrences in 1043 children). In children treated with a high total dose of primaquine, the corresponding adjusted HR was 0·75 (0·48–1·18, 97 recurrences in 1198 children; figure 2, appendix p 34).

**Figure 3:**
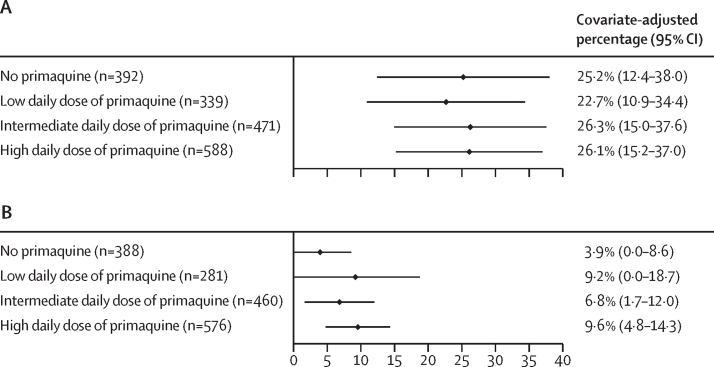
Estimated percentage of children younger than 15 years with *P vivax* malaria experiencing gastrointestinal symptoms (composite outcome) on days 1–2 (A) and on days 5–7 (B) A low daily dose of primaquine was about 0·25 mg/kg, an intermediate daily dose was about 0·5 mg/kg, and a high daily dose was about 1 mg/kg. Generalised estimating equations modified Poisson regression models adjusting for age, sex (male or female), and (log_10_) baseline parasite density, clustering by study site and cluster robust error estimates. Covariate-adjusted percentages were estimated at the mean values of the covariates. *P vivax*=*Plasmodium vivax*.

A total of 1993 children from 15 studies^15^, ^17^, ^18^, ^19^, ^20^, ^22^, ^24^, ^25^, ^26^, ^27^, ^28^, ^29^, ^32^, ^36^ were included in the gastrointestinal tolerability meta-analysis: 378 (19·0%) were younger than 5 years and 1615 (81·0%) were aged 5 years to younger than 15 years (appendix pp 36–37). The unadjusted risk of gastrointestinal symptoms in children (age <15 years) was lower on days 5–7 (ranging from 2·8% to 11·0%), after the acute effects of malaria had abated, compared with the corresponding risks on day 0 (49·4–64·6%), and days 1–2 (28·2–38·4%; appendix p 38). Compared with no primaquine, children treated with any dose of primaquine had a greater risk of gastrointestinal symptoms on days 5–7 after adjustment for confounders, with adjusted risks of 3·9% (95% CI 0–8·6) in children not treated with primaquine, 9·2% (0–18·7) with a low daily dose of primaquine, 6·8% (1·7–12·0) with an intermediate daily dose of primaquine, and 9·6% (4·8–14·3) with a high daily dose of primaquine (figure 3; appendix p 39). Sensitivity analyses were consistent (appendix pp 40–43). The risk of vomiting in children within an hour of taking primaquine on days 3−13 was low (0·0–0·3%) at any daily dose (appendix p 44).

**Figure 4:**
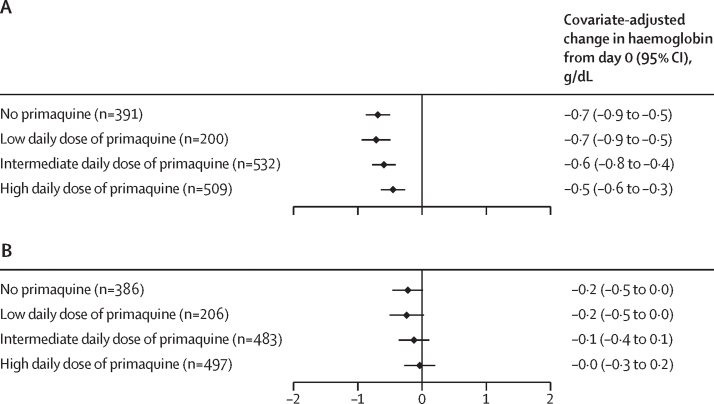
Estimated change in haemoglobin from day 0 to days 2–3 (A) and to days 5–7 (B) in children younger than 15 years with *P vivax* malaria A low daily dose of primaquine was about 0·25 mg/kg, an intermediate daily dose was about 0·5 mg/kg, and a high daily dose was about 1 mg/kg. Mixed-effects linear regression models adjusting for age, sex (male or female), (log_10_) baseline parasite density, and day 0 haemoglobin with random intercepts by study site. Covariate-adjusted changes in haemoglobin were estimated at the mean values of the covariates. *P vivax*=*Plasmodium vivax*.

The analysis of haematological safety included data on 2539 children from 18 studies in 12 countries,^15^, ^16^, ^17^, ^19^, ^20^, ^21^, ^22^, ^23^, ^24^, ^25^, ^26^, ^27^, ^29^, ^30^, ^32^, ^34^, ^38^ of whom 455 (17·9%) were younger than 5 years and 2084 (82·1%) were aged 5 years to younger than 15 years (appendix p 45). Of the 2144 patients with G6PD activity of 30% or higher, the primary haematological safety outcome could be assessed in 1869 children younger than 15 years (mean haemoglobin on day 0 of 11·6 g/dL [SD 1·5]). A haemoglobin decrease of at least 25% to less than 7 g/dL was observed in nine (0·5%) of 1869 children, (0·4% [95% CI 0·1–1·5] after no primaquine, 0·0% [0·0–1·6] after a low daily dose, 0·5% [0·1–1·4] after an intermediate daily dose, and 0·7% [0·2–1·9] after a high daily dose of primaquine). Seven of the nine children with severe haemolysis were female (table 1; appendix p 46). After adjustment for confounders, there were no notable differences in the mean absolute change in haemoglobin on days 2–3 or days 5–7 after primaquine treatment at any daily dose relative to treatment without primaquine in patients with G6PD activity of 30% or higher (figure 4).

**Table 1:** Haematological outcomes in children with *P vivax* malaria with G6PD activity of 30% or higher

		**No primaquine (n=475)**	**Low daily dose of primaquine (n=236)**	**Intermediate daily dose of primaquine (n=612)**	**High daily dose of primaquine (n=546)**
**Primary haematology outcome**					

Decrease in haemoglobin of ≥25% and haemoglobin below 7 g/dL from day 0 to day of lowest haemoglobin between days 1 and 13		2 (0·4%; 0·1–1·5)	0 (0·0%; 0·0–1·6)	3 (0·5%; 0·1–1·4)	4 (0·7%; 0·2–1·9)

**Secondary haematology outcomes**					

Decrease in haemoglobin of >5 g/dL from day 0 to day of lowest haemoglobin between days 1 and 13		0 (0·0%; 0·0–0·8)	0 (0·0%; 0·0–1·6)	1 (0·2%; 0·0–0·9)	2 (0·4%; 0·0–1·3)

Anaemia on days 2 or 3					

	Nil	228/283 (80·6%)	122/142 (85·9%)	265/387 (68·5%)	275/377 (72·9%)

	Mild (haemoglobin starts ≥11 g/dL and falls to <11 g/dL but ≥8 g/dL)	51/283 (18·0%)	19/142 (13·4%)	112/387 (28·9%)	93/377 (24·7%)

	Moderate (haemoglobin starts ≥8 g/dL and falls to <8 g/dL but ≥5 g/dL)	4/283 (1·4%)	1/142 (0·7%)	10/387 (2·6%)	9/377 (2·4%)

Anaemia on days 5−7					

	Nil	276/316 (87·3%)	134/148 (90·5%)	293/381 (76·9%)	285/372 (76·6%)

	Mild (haemoglobin starts ≥11 g/dL and falls to <11 g/dL but ≥8 g/dL)	40/316 (12·7%)	12/148 (8·1%)	81/381 (21·3%)	80/372 (21·5%)

	Moderate (haemoglobin starts ≥8 g/dL and falls to <8 g/dL but ≥5 g/dL)	0/316	2/148 (1·4%)	7/381 (1·8%)	6/372 (1·6%)

	Severe (haemoglobin starts ≥5 g/dL and falls to <5 g/dL)	0/316	0/148	0/381	1/372 (0·3%)

Blood transfusion		0/380	0/147	0/599	1/541 (0·2%)


Data are n (%; 95% CI) or n/N (%). Data on anaemia on days 2–3 were not available for 680 children and on days 5–7 were not available for 652 children. G6PD=glucose-6-phosphate dehydrogenase. *P vivax*=*Plasmodium vivax*.

A decrease in haemoglobin of more than 5 g/dL within 14 days occurred in three (0·2%) of 1869 children, one of whom was treated with an intermediate daily dose and two with a high daily dose of primaquine (table 1). In 283 children treated without primaquine, 51 (18·0%) developed mild anaemia (haemoglobin 8 g/dL to <11 g/dL) and four (1·4%) developed moderate anaemia (haemoglobin 5 g/dL to <8 g/dL) on days 2–3 (table 1). These proportions increased with the dose of primaquine, particularly at intermediate and high daily doses. The risks of mild or moderate anaemia on days 5–7 were similar to those on days 2–3. One (0·3%) child of 372 treated with a high daily dose of primaquine developed severe anaemia on days 5–7, with a minimum haemoglobin of 4·4 g/dL (table 1; appendix p 46). In a post-hoc analysis, after adjustment for confounders, the proportion of children with anaemia of any severity on days 2–3 or days 5–7 did not differ with primaquine use or daily dose compared with treatment without primaquine (appendix p 47). One female child who was treated with a high daily dose of primaquine required a blood transfusion (table 1; appendix p 46).

Adverse events were reported in six studies in six countries,^19^, ^20^, ^21^, ^22^, ^23^, ^26^ with data available from 1797 children, of whom 403 (22·4%) had at least one adverse event (appendix pp 48–50). Overall, the unadjusted proportions of children with adverse events, and some adverse events of special interest (abdominal pain, anaemia, and methaemoglobinaemia) were generally higher in the primaquine groups than in groups without primaquine and generally highest in children treated with a high daily dose of primaquine (appendix pp 51–53). This trend was not apparent with other adverse events of special interest (diarrhoea, anorexia, or vomiting; appendix pp 54–56). There was no difference in all adverse events across age categories; however, children younger than 5 years reported abdominal pain less frequently than children aged 5 years to younger than 15 years (appendix pp 50–51). Adverse events due to methaemoglobin were rare and occurred in 11 (0·6%) children (appendix p 53).

A total of 20 (1·1%) serious adverse events were reported in children within 28 days of primaquine administration (or equivalent time schedule in groups without primaquine), with a similar proportion of patients with serious adverse events across age categories (table 2). In children treated with primaquine (n=1289), there were 17 (1·3%) serious adverse events, of which 15 were considered to be related to the drug. Of the drug-related serious adverse events, two occurred following low, four following intermediate, and nine following high daily doses of primaquine (appendix p 57).

**Table 2:** Children with *P vivax* or mixed malaria infection reporting at least one serious adverse event occurring within 28 days of initiating primaquine (or equivalent time schedule in placebo and no primaquine groups), by age category and primaquine dose

	**All (n=1797)**	**Age <5 years (n=293)**	**Age 5 to <15 years (n=1504)**
Any serious adverse event	20 (1·1%)	2 (0·7%)	18 (1·2%)

No primaquine	3/508* (0·6%)	0/94	3/414 (0·7%)

Low daily dose	2/185† (1·1%)	0/29	2/156 (1·3%)

Intermediate daily dose	4/561‡ (0·7%)	1/89 (1·1%)	3/472 (0·6%)

High daily dose	11/543§ (2·0%)	1/81 (1·2%)	10/462 (2·2%)


Data are n (%) or n/N (%). A low daily dose is defined as about 0·25 mg/kg per day, intermediate daily dose as about 0·5 mg/kg per day, and a high daily dose as about 1 mg/kg per day. *P vivax*=*Plasmodium vivax*.

* Serious adverse events and causality to primaquine were severe vomiting (unrelated; n=1), dengue fever (unrelated; n=1), and dry mouth and taste disorder (unrelated; n=1).

† Serious adverse events and causality to primaquine were jaundice and haemoglobinuria (possibly related; n=1), and lethargy, tachycardia, and abdominal pain (possibly related; n=1).

‡ Serious adverse events and causality to primaquine were haemolysis (probably related; n=1), methaemoglobinaemia and scrub typhus (probably related; n=1), methaemoglobinaemia (probably related; n=1), and pneumonia and oesophagitis (possibly related; n=1).

§ Serious adverse events and causality to primaquine were haemolysis (possibly related; n=1), haemolysis (probably related; n=1), methaemoglobinaemia (definitely related; n=1), methaemolglobinaemia (probably related; n=3), vomiting (possibly related; n=1), dyspepsia (possibly related; n=1), diarrhoea (possibly related; n=1), vomiting (unrelated; n=1), and asthma (unrelated; n=1). Additional details on serious adverse events are provided in the appendix (pp 57–59).

## Discussion

Our individual patient data meta-analysis highlights the benefits and risks of different primaquine dosing regimens in children. A total dose of 7 mg/kg primaquine reduced the rate of recurrence by more than 40% compared with 3·5 mg/kg and by about 60% in the subgroup of children younger than 5 years. An increase in the total dose requires a higher daily dose or longer duration of treatment. Although there were more serious adverse events in children treated with a high (1 mg/kg) than with a low (0·25 mg/kg) or an intermediate (0·5 mg/kg) daily dose of primaquine, in adjusted analyses there were no increases with a high or an intermediate daily dose compared with a low daily dose of primaquine in gastrointestinal or haemolytic adverse effects in children with G6PD activity of 30% or higher.

An estimated 85% of recurrences are caused by relapses;^41^ hence, it is crucial that children and adults receive appropriate radical cure treatment. The current absence of child-friendly primaquine formulations hampers effective implementation of primaquine radical cure in children, who have a disproportionately high burden of *P vivax*-associated anaemia.^3^ Inclusion of paediatric primaquine formulations in the WHO Antimalarial Expression of Interest List since 2019 has highlighted the need to develop a dispersible formulation. Results from our meta-analysis support the use of a higher total dose of primaquine (7 mg/kg) in children, which almost halved the rate of recurrence compared with a low total dose (3·5 mg/kg). The reduced efficacy of low dose primaquine was particularly apparent in children younger than 5 years, who had only a 67% reduction in recurrences compared with no primaquine treatment, versus an 85% reduction in children aged 5 years to younger than 15 years. In young children, a high total dose of primaquine reduced recurrences by 59% compared with low-dose primaquine. These results suggest that younger children might require a higher total dose to achieve the same anti-relapse benefit as older individuals. Allometric scaling has been used to derive an appropriate weight-based dosing regimen for 0·5 mg/kg per day primaquine dosing.^42^

Administration of a high total dose of primaquine requires a 14-day regimen at 0·5 mg/kg per day or 7-day regimen at 1 mg/kg per day. In children with G6PD activity of 30% or higher, there was no increased risk of haemolysis at a population level with primaquine doses of 0·5 mg/kg per day or 1 mg/kg per day. However, seven of nine patients with severe haemolysis received higher daily doses; CIs around these estimates were wide, and we were unable to undertake an adjusted analysis of these events due to their uncommon occurrence. Female patients were over-represented in participants with severe haemolysis, which could potentially reflect heterozygous female patients with intermediate G6PD activity (30–70%) at increased risk of severe haemolysis.

The gastrointestinal analysis was restricted to analysing the risk of vomiting, diarrhoea, and anorexia, since reports of nausea and abdominal pain were considered to be unreliable in young children. This potentially reflects the reason that fewer adverse events related to abdominal pain were documented in young children. There was considerable heterogeneity of symptoms between study sites in the same study and between studies.^43^ This probably results from multiple factors, including differences in the way symptoms were elicited, cultural differences in reporting symptoms, and variability in the co-administration of medication with food. Potential bias relating to this variation was mitigated through the comparison of primaquine doses to no primaquine. Furthermore, the results were similar in sensitivity analyses that excluded one study at a time or restricted the analysis to studies in which primaquine was compared directly with patients not treated with primaquine. Individual patient data on food intake were not available for our analysis; however, recent studies highlight markedly improved gastrointestinal tolerability when primaquine is co-administered with food.^44^, ^45^

Data on adverse events and serious adverse events were only available from six (22%) of 27 studies, but these included 1797 (51%) of 3514 children. Although all studies used the same internationally recognised definitions for adverse events and serious adverse events, the way data are reported by patients (or caregivers) and elicited and recorded by study staff can vary, either knowingly or not. One study acknowledged the high inter-site variability in adverse events and also restricted adverse events to grade 3 or worse.^26^ For example, despite anaemia potentially being a more objective adverse event due to the ability to measure patients' haemoglobin concentrations, there was evidence of reporting bias with anaemia adverse event recording. All anaemia adverse events occurred in patients randomly assigned to primaquine groups in three of the six studies, with almost all of these reported in one study. The results of the haemolysis safety analysis are likely to provide a more reliable and generalisable assessment of haematological safety.

Our meta-analysis had some other limitations. Only 39% of all eligible studies were included in the analysis; however, the included studies were undertaken more recently and followed up patients for longer. Data were available from very few patients younger than 1 year, preventing generalisation to this population. Actual dosing information was only available for 67% of patients in the efficacy analysis, with dosing for the remainder derived from weight-based or age-based protocols. This did not substantially bias results in a sensitivity analysis restricting inclusion only to patients for whom actual dosing was available. Short follow-up (<180 days in six of the 19 studies) potentially underestimates the benefit of primaquine use compared to no primaquine or increased primaquine doses due to a lower chance of developing a recurrence before study cessation. A sensitivity analysis restricted to studies with follow-up of 180 days or longer did not substantially change results. Methodological limitations of models in our analysis include the presence of unmeasured confounding and inherent potential for bias in HRs.

Although estimates in the risk of recurrence could be generated, anti-relapse efficacy could not be derived because there is no reliable way of distinguishing whether recurrent parasitaemia was due to relapses, recrudescence, or reinfection. Anti-relapse efficacy is further confounded by post-schizontocidal treatment prophylaxis, differing regional relapse periodicities, and differing transmission intensities.^46^ The effect of regional differences can potentially be seen in Melanesia, where two studies had a substantially higher rate of recurrence at day 90, even after treatment with a high total dose primaquine (>28%^27^ compared with all other sites where the risk of recurrence was <7%), which might reflect higher local transmission intensity and relapse periodicity.^46^ In addition, there might be *CYP2D6* host genotypic differences in the studies that were not reported or genetic variations in the parasite leading to compromised primaquine efficacy.

Although the majority of patients in our efficacy analysis were from the Asia-Pacific region, 22% of patients were enrolled from the Americas and Africa, suggesting the results are generalisable to most endemic countries. Tolerability and safety findings from our analysis are also likely to be generalisable to the use of primaquine for treatment of malaria in children caused by *P ovale*.

In summary, our analysis suggests that increasing the total dose of primaquine from 3·5 mg/kg to 7 mg/kg would reduce the risk of recurrent *P vivax* parasitaemia by more than 40% in all children and by about 60% in children younger than 5 years. A higher total dose of primaquine could be achieved by administering 0·5 mg/kg per day for 14 days, which seemed to be safe and highly effective in patients with G6PD activity of 30% or higher. A higher daily dose of 1 mg/kg administered for 7 days might provide greater adherence and thus effectiveness through a shorter course regimen, but this might come with a greater risk of severe haemolysis and thus should potentially be reserved for patients with G6PD activity of 70% or higher. Our study provides important evidence that will inform optimal weight-based primaquine dosing guidelines for children and the development of novel paediatric primaquine formulations.

### WWARN Paediatric Primaquine Vivax Study Group

### Contributors

### Data sharing

Pseudonymised participant data used in this analysis are available for access via the WWARN website. Requests for access will be reviewed by a data access committee to ensure that use of data protects the interests of the participants and researchers according to the terms of ethics approval and principles of equitable data sharing. Requests can be submitted by email to malariaDAC@iddo.org via the data access form. WWARN is registered with the Registry of Research Data Repositories.

## Declaration of interests

JKB reports institutional research funding from MMV, GSK, Wellcome Trust, and Sanaria; participation on the US National Institutes of Health data safety monitoring board; and membership of the editorial board of Travel Medicine and Infectious Disease and the guidelines development group for malaria control and elimination, Global Malaria Programme, WHO. RJC, JKB, and RNP report contributions to Up-to-Date. All other authors declare no competing interests.

## References

[1] Price RN, Commons RJ, Battle KE, Thriemer K, Mendis K (2020). *Plasmodium vivax* in the era of the shrinking *P. falciparum* map. Trends Parasitol.

[2] Chu CS, White NJ (2021). The prevention and treatment of *Plasmodium vivax* malaria. PLoS Med.

[3] Douglas NM, Lampah DA, Kenangalem E (2013). Major burden of severe anemia from non-falciparum malaria species in Southern Papua: a hospital-based surveillance study. PLoS Med.

[4] WHO 22nd Invitation to Manufacturers of Antimalarial Medicine to Submit an Expression of Interest (EOI) for Product Evaluation to the WHO Prequalification Unit (PQT). Geneva, Switzerland, 2023. https://extranet.who.int/prequal/sites/default/files/document_files/EOI-MalariaV22.pdf.

[5] Baird JK (2019). 8-aminoquinoline therapy for latent malaria. Clin Microbiol Rev.

[6] Commons RJ, Thriemer K, Humphreys G (2017). The vivax surveyor: online mapping database for *Plasmodium vivax* clinical trials. Int J Parasitol Drugs Drug Resist.

[7] CFAST Malaria Team Therapeutic Area Data Standards User Guide for Uncomplicated Malaria Revision History, Version 1.0 (Provisional): CDISC, 2017. https://www.cdisc.org/standards/therapeutic-areas/malaria/malaria-therapeutic-area-user-guide-v1-0.

[8] Infectious Diseases Data Observatory (2023). IDDO SDTM implementation manual. https://wiki.iddo.org/en/Data-Engineering/IDDO-SDTM-Implementation-Manual.

[9] John GK, Douglas NM, von Seidlein L (2012). Primaquine radical cure of *Plasmodium vivax*: a critical review of the literature. Malar J.

[10] Lee SJ, Stepniewska K, Anstey N (2008). The relationship between the haemoglobin concentration and the haematocrit in *Plasmodium falciparum* malaria. Malar J.

[11] Higgins JPT, Savović J, Page MJ, Elbers RG JAC S. Chapter 8: Assessing risk of bias in a randomized trial. In: Higgins JPT, Thomas J, Chandler J, et al., eds. Cochrane Handbook for Systematic Reviews of Interventions version 63 (updated February 2022): Cochrane; 2022. https://training.cochrane.org/handbook/current/chapter-08#section-8-9.

[12] Munn Z, Moola S, Lisy K, Riitano D, Tufanaru C (2015). Methodological guidance for systematic reviews of observational epidemiological studies reporting prevalence and cumulative incidence data. Int J Evid-Based Healthc.

[13] Commons RJ, Simpson JA, Thriemer K (2019). The haematological consequences of *Plasmodium vivax* malaria after chloroquine treatment with and without primaquine: a WorldWide Antimalarial Resistance Network systematic review and individual patient data meta-analysis. BMC Med.

[14] Vivax and Ovale Paediatric Primaquine Study Group Vivax and Ovale Paediatric Primaquine Study Group Statistical Analysis Plan, v1.3 - 21 June 2022. United Kingdom: WorldWide Antimalarial Resitance Network, 2022. https://www.iddo.org/document/statistical-analysis-plan-vivax-paediatric-primaquine.

[15] Hasugian AR, Purba HL, Kenangalem E (2007). Dihydroartemisinin–piperaquine versus artesunate–amodiaquine: superior efficacy and posttreatment prophylaxis against multidrug-resistant *Plasmodium falciparum* and *Plasmodium vivax* malaria. Clin Infect Dis.

[16] Leslie T, Mayan I, Mohammed N (2008). A randomised trial of an eight-week, once weekly primaquine regimen to prevent relapse of *Plasmodium vivax* in Northwest Frontier Province, Pakistan. PLoS One.

[17] Pasaribu AP, Chokejindachai W, Sirivichayakul C (2013). A randomized comparison of dihydroartemisinin–piperaquine and artesunate–amodiaquine combined with primaquine for radical treatment of vivax malaria in Sumatera, Indonesia. J Infect Dis.

[18] Gonzalez-Ceron L, Rodriguez MH, Sandoval MA (2015). Effectiveness of combined chloroquine and primaquine treatment in 14 days versus intermittent single dose regimen, in an open, non-randomized, clinical trial, to eliminate *Plasmodium vivax* in southern Mexico. Malar J.

[19] Abreha T, Hwang J, Thriemer K (2017). Comparison of artemether-lumefantrine and chloroquine with and without primaquine for the treatment of *Plasmodium vivax* infection in Ethiopia: a randomized controlled trial. PLoS Med.

[20] Awab GR, Imwong M, Bancone G (2017). Chloroquine–primaquine versus chloroquine alone to treat vivax malaria in Afghanistan: an open randomized superiority trial. Am J Trop Med Hyg.

[21] Chu CS, Phyo AP, Lwin KM (2018). Comparison of the cumulative efficacy and safety of chloroquine, artesunate, and chloroquine–primaquine in *Plasmodium vivax* malaria. Clin Infect Dis.

[22] Hamid MMA, Thriemer K, Elobied ME (2018). Low risk of recurrence following artesunate–sulphadoxine–pyrimethamine plus primaquine for uncomplicated *Plasmodium falciparum* and *Plasmodium vivax* infections in the Republic of the Sudan. Malar J.

[23] Chu CS, Phyo AP, Turner C (2019). Chloroquine versus dihydroartemisinin–piperaquine with standard high-dose primaquine given either for 7 days or 14 days in *Plasmodium vivax* malaria. Clin Infect Dis.

[24] Ladeia-Andrade S, Menezes MJ, de Sousa TN (2019). Monitoring the efficacy of chloroquine–primaquine therapy for uncomplicated *Plasmodium vivax* malaria in the main transmission hot spot of Brazil. Antimicrob Agents Chemother.

[25] Rijal KR, Adhikari B, Ghimire P (2019). Efficacy of primaquine in preventing short- and long-latency *Plasmodium vivax* relapses in Nepal. J Infect Dis.

[26] Taylor WRJ, Thriemer K, von Seidlein L (2019). Short-course primaquine for the radical cure of *Plasmodium vivax* malaria: a multicentre, randomised, placebo-controlled non-inferiority trial. Lancet.

[27] Poespoprodjo JR, Burdam FH, Candrawati F (2022). Supervised versus unsupervised primaquine radical cure for the treatment of falciparum and vivax malaria in Papua, Indonesia: a cluster-randomised, controlled, open-label superiority trial. Lancet Infect Dis.

[28] Abdallah TM, Ali AA, Bakri M, Gasim GI, Musa IR, Adam I (2012). Efficacy of artemether−lumefantrine as a treatment for uncomplicated *Plasmodium vivax* malaria in eastern Sudan. Malar J.

[29] Barber BE, William T, Grigg MJ (2013). A prospective comparative study of knowlesi, falciparum, and vivax malaria in Sabah, Malaysia: high proportion with severe disease from *Plasmodium knowlesi* and *Plasmodium vivax* but no mortality with early referral and artesunate therapy. Clin Infect Dis.

[30] Marques MM, Costa MR, Santana Filho FS (2014). *Plasmodium vivax* chloroquine resistance and anemia in the western Brazilian Amazon. Antimicrob Agents Chemother.

[31] Gomes MS, Vieira JL, Machado RL (2015). Efficacy in the treatment of malaria by *Plasmodium vivax* in Oiapoque, Brazil, on the border with French Guiana: the importance of control over external factors. Malar J.

[32] Thanh PV, Hong NV, Van NV (2015). Confirmed *Plasmodium vivax* resistance to chloroquine in central Vietnam. Antimicrob Agents Chemother.

[33] Yuan L, Wang Y, Parker DM (2015). Therapeutic responses of *Plasmodium vivax* malaria to chloroquine and primaquine treatment in northeastern Myanmar. Antimicrob Agents Chemother.

[34] Ley B, Alam MS, Thriemer K (2016). G6PD deficiency and antimalarial efficacy for uncomplicated malaria in Bangladesh: a prospective observational study. PLoS One.

[35] Longley RJ, Sripoorote P, Chobson P (2016). High efficacy of primaquine treatment for *Plasmodium vivax* in western Thailand. Am J Trop Med Hyg.

[36] Zuluaga-Idárraga L, Blair S, Akinyi Okoth S (2016). Prospective study of *Plasmodium vivax* malaria recurrence after radical treatment with a chloroquine–primaquine standard regimen in Turbo, Colombia. Antimicrob Agents Chemother.

[37] Brasil LW, Rodrigues-Soares F, Santoro AB (2018). CYP2D6 activity and the risk of recurrence of *Plasmodium vivax* malaria in the Brazilian Amazon: a prospective cohort study. Malar J.

[38] Grigg MJ, William T, Barber BE (2018). Age-related clinical spectrum of *Plasmodium knowlesi* malaria and predictors of severity. Clin Infect Dis.

[39] de Sena LWP, Mello AGNC, Ferreira MVD, de Ataide MA, Dias RM, Vieira JLF (2019). Doses of chloroquine in the treatment of malaria by *Plasmodium vivax* in patients between 2 and 14 years of age from the Brazilian Amazon basin. Malar J.

[40] Xu S, Zeng W, Ngassa Mbenda HG (2020). Efficacy of directly-observed chloroquine-primaquine treatment for uncomplicated acute *Plasmodium vivax* malaria in northeast Myanmar: a prospective open-label efficacy trial. Travel Med Infect Dis.

[41] Commons RJ, Simpson JA, Watson J, White NJ, Price RN (2020). Estimating the proportion of *Plasmodium vivax* recurrences caused by relapse: a systematic review and meta-analysis. Am J Trop Med Hyg.

[42] Taylor WR, Hoglund RM, Peerawaranun P (2021). Development of weight and age-based dosing of daily primaquine for radical cure of vivax malaria. Malar J.

[43] Thriemer K, Commons RJ, Rajasekhar M (2023). The heterogeneity of symptom reporting across study sites: a secondary analysis of a randomised placebo-controlled multicentre antimalarial trial. BMC Med Res Methodol.

[44] Thriemer K, Degaga TS, Christian M (2023). Primaquine radical cure in patients with *Plasmodium falciparum* malaria in areas co-endemic for *P falciparum* and *Plasmodium vivax* (PRIMA): a multicentre, open-label, superiority randomised controlled trial. Lancet.

[45] Woon SA, Moore BR, Laman M (2023). Ultra-short course, high-dose primaquine to prevent *Plasmodium vivax* infection following uncomplicated pediatric malaria: a randomized, open-label, non-inferiority trial of early versus delayed treatment. Int J Infect Dis.

[46] White NJ (2011). Determinants of relapse periodicity in *Plasmodium vivax* malaria. Malar J.

