# Supplementary material for: Primaquine for uncomplicated Plasmodium vivax malaria in children younger than 15 years: a systematic review and individual patient data meta-analysis
Source: Lancet Child Adolesc Health. 2024 Nov;8(11):798–808. doi: 10.1016/S2352-4642(24)00210-4 (PMC11480364; doi:10.1016/S2352-4642(24)00210-4)
Supplement: Supplementary appendix [file mmc1.pdf]

THE LANCET
Child & Adolescent Health

Supplementary appendix

This appendix formed part of the original submission and has been peer reviewed. We post it as supplied by the authors.

Supplement to: Commons RJ, Rajasekhar M, Allen EN, et al. Primaquine for uncomplicated *Plasmodium vivax* malaria in children younger than 15 years: a systematic review and individual patient data meta-analysis. *Lancet Child Adolesc Health* 2024; published online Sept 24. https://doi.org/10.1016/S2352-4642(24)00210-4.

APPENDIX

Commons RJ, Rajasekhar M, et al, Primaquine for uncomplicated *Plasmodium vivax* malaria in children younger than 15 years: a systematic review and individual patient data meta-analysis

	Page
**Checklist S1.** PRISMA-IPD	3
**Text S1.** Supplementary methods	7
**Figure S1.** Directed acyclic graphs for the relationship between primaquine dose (exposure) and outcomes in children <15 years	12
**Table S1.** Studies included in analysis	13
**Table S2.** Study testing and inclusion criteria	14
**Table S3.** Study sites included in analysis	16
**Table S4.** Reasons for studies not being included in analyses	18
**Table S5.** Studies eligible for the analyses but not included	19
**Figure S2.** Location of study sites for included studies	21
**Table S6.** Risk of bias assessment in randomised controlled studies	22
**Table S7.** Risk of bias assessment in single arm observational studies	23
**Table S8.** Comparison of baseline characteristics between included studies and eligible but not included studies	24
**Table S9.** Demographics and baseline characteristics for efficacy analysis by primaquine category	25
**Figure S3.** Box plot of primaquine dose by age	27
**Figure S4.** Scatter plot of primaquine dose by A) age and B) weight categorised by presence of recurrence between day 7 and day 180	28
**Figure S5.** Kaplan-Meier figure of cumulative incidence of recurrence between day 7 and day 365 for A) total primaquine dosing and use and B) intermittent primaquine dosing	29
**Table S10.** Cumulative incidence of recurrence between day 7 and days 42, 90, 180 and 365 by study and primaquine category	30
**Table S11.** Sensitivity analyses estimating hazard ratios for *Plasmodium vivax* recurrence between days 7 and 180 by total primaquine dose	32
**Table S12.** Sensitivity analysis excluding one study at a time for hazard ratio of *Plasmodium vivax* recurrence between days 7 and 180 following primaquine treatment group compared with no primaquine	33
**Text S2.** Additional efficacy analysis results	34
**Figure S6.** Adjusted hazard ratio for recurrence between day 7 and 180 compared with primaquine total mg/kg dose for children	35
**Table S13.** Baseline characteristics and demographic features for gastrointestinal tolerability analysis by age categories	36
**Figure S7.** Distribution of primaquine daily dose by primaquine mg/kg daily dose category and age category	37

Figure S8. Unadjusted risk of gastrointestinal symptoms in children on day 0, days 1-2 and days 5-7 38

Figure S9. Covariate-adjusted estimates for gastrointestinal intolerance on days 5-7 by primaquine daily dose categories and age categories 39

Table S14. Covariate-adjusted estimated percentages (95% CI) from sensitivity analyses for the composite gastrointestinal tolerability outcome assessed on days 5-7 40

Table S15. Covariate-adjusted estimated percentages (95% CI) from sensitivity analyses for the composite gastrointestinal tolerability outcome assessed on days 1-2, restricted to patients who started primaquine on day 0 41

Table S16. Contribution of patients from each study included in the analysis of the primary composite gastrointestinal tolerability end point on days 5-7 to each primaquine dose category 42

Figure S10. Sensitivity analysis for the covariate-adjusted estimated percentage of patients reporting gastrointestinal symptoms (95% CI) assessed on days 5-7, excluding one study at a time 43

Table S17. Risk of acute vomiting on days 0-2 and 3-13 by primaquine daily dose categories in children 44

Table S18. Baseline characteristics and demographic features of patients included in the haematological safety analysis by age groups 45

Table S19. Details of patients experiencing haematological outcomes 46

Figure S11. Estimated percentage of children with G6PD activity ≥30% developing mild, moderate or severe anaemia on days 2-3 or days 5-7 by primaquine daily dose groups 47

Table S20. Baseline characteristics and demographic features of patients in the adverse event meta-analysis, by age category 48

Table S21. Patients reporting at least one adverse event within 28 days of initiating primaquine (or equivalent time schedule in placebo / no primaquine arms) in patients with *P. vivax* or mixed malaria infection, by age category and primaquine dose 50

Table S22. Patients with any abdominal pain within 28 days of initiating primaquine (or equivalent time schedule in placebo / no primaquine arms) in patients with *P. vivax* or mixed malaria infection, by age category and primaquine dose 51

Table S23. Patients with any anaemia within 28 days of initiating primaquine (or equivalent time schedule in placebo / no primaquine arms) in patients with *P. vivax* or mixed malaria infection, by age category and primaquine dose 52

Table S24. Patients with any methaemoglobinaemia reported within 28 days of initiating primaquine (or equivalent time schedule in placebo / no primaquine arms) in patients with *P. vivax* or mixed malaria infection, by age category and primaquine dose 53

Table S25. Patients with any diarrhoea within 28 days of initiating primaquine (or equivalent time schedule in placebo / no primaquine arms) in patients with *P. vivax* or mixed malaria infection, by age category and primaquine dose 54

Table S26. Patients with any anorexia within 28 days of initiating primaquine (or equivalent time schedule in placebo / no primaquine arms) in patients with *P. vivax* or mixed malaria infection, by age category and primaquine dose 55

Table S27. Patients with any vomiting within 28 days of initiating primaquine (or equivalent time schedule in placebo / no primaquine arms) in patients with *P. vivax* or mixed malaria infection, by age category and primaquine dose 56

Table S28: Description of serious adverse events considered by study investigator to be primaquine related (possibly, probably or definitely related) 57

References S1. 60

Checklist S1. PRISMA-IPD Checklist of items to include when reporting a systematic review and meta-analysis of individual participant data (IPD)

PRISMA-IPD Section/topic	Item No	Checklist item	Reported on page
**Title**			
Title	1	Identify the report as a systematic review and meta-analysis of individual participant data.	1
**Abstract**			
Structured summary	2	Provide a structured summary including as applicable:	8
		**Background**: state research question and main objectives, with information on participants, interventions, comparators and outcomes.	
		**Methods**: report eligibility criteria; data sources including dates of last bibliographic search or elicitation, noting that IPD were sought; methods of assessing risk of bias.	
		**Results**: provide number and type of studies and participants identified and number (%) obtained; summary effect estimates for main outcomes (benefits and harms) with confidence intervals and measures of statistical heterogeneity. Describe the direction and size of summary effects in terms meaningful to those who would put findings into practice.	9
		**Discussion:** state main strengths and limitations of the evidence, general interpretation of the results and any important implications.	
		**Other:** report primary funding source, registration number and registry name for the systematic review and IPD meta-analysis.	
**Introduction**			
Rationale	3	Describe the rationale for the review in the context of what is already known.	11
Objectives	4	Provide an explicit statement of the questions being addressed with reference, as applicable, to participants, interventions, comparisons, outcomes and study design (PICOS). Include any hypotheses that relate to particular types of participant-level subgroups.	11-12
**Methods**			
Protocol and registration	5	Indicate if a protocol exists and where it can be accessed. If available, provide registration information including registration number and registry name. Provide publication details, if applicable.	13
Eligibility criteria	6	Specify inclusion and exclusion criteria including those relating to participants, interventions, comparisons, outcomes, study design and characteristics (e.g. years when conducted, required minimum follow-up). Note whether these were applied at the study or individual level i.e. whether eligible participants were included (and ineligible participants excluded) from a study that included a wider population than specified by the review inclusion criteria. The rationale for criteria should be stated.	13; Text S1
Identifying studies -	7	Describe all methods of identifying published and unpublished studies including, as applicable: which bibliographic databases were searched with dates of coverage; details of any hand searching including of conference proceedings; use of study registers	13; Text S1

information sources		and agency or company databases; contact with the original research team and experts in the field; open adverts and surveys. Give the date of last search or elicitation.	
Identifying studies - search	8	Present the full electronic search strategy for at least one database, including any limits used, such that it could be repeated.	Text S1
Study selection processes	9	State the process for determining which studies were eligible for inclusion.	13; Text S1
Data collection processes	10	Describe how IPD were requested, collected and managed, including any processes for querying and confirming data with investigators. If IPD were not sought from any eligible study, the reason for this should be stated (for each such study).<br>If applicable, describe how any studies for which IPD were not available were dealt with. This should include whether, how and what aggregate data were sought or extracted from study reports and publications (such as extracting data independently in duplicate) and any processes for obtaining and confirming these data with investigators.	13; Ref 7-8
Data items	11	Describe how the information and variables to be collected were chosen. List and define all study level and participant level data that were sought, including baseline and follow-up information. If applicable, describe methods of standardising or translating variables within the IPD datasets to ensure common scales or measurements across studies.	13-14; Text S1
IPD integrity	A1	Describe what aspects of IPD were subject to data checking (such as sequence generation, data consistency and completeness, baseline imbalance) and how this was done.	Ref 7-8
Risk of bias assessment in individual studies.	12	Describe methods used to assess risk of bias in the individual studies and whether this was applied separately for each outcome. If applicable, describe how findings of IPD checking were used to inform the assessment. Report if and how risk of bias assessment was used in any data synthesis.	14-15
Specification of outcomes and effect measures	13	State all treatment comparisons of interests. State all outcomes addressed and define them in detail. State whether they were pre-specified for the review and, if applicable, whether they were primary/main or secondary/additional outcomes. Give the principal measures of effect (such as risk ratio, hazard ratio, difference in means) used for each outcome.	14-16, Box 1, Text S1
Synthesis methods	14	Describe the meta-analysis methods used to synthesise IPD. Specify any statistical methods and models used. Issues should include (but are not restricted to):<br>• Use of a one-stage or two-stage approach.<br>• How effect estimates were generated separately within each study and combined across studies (where applicable).<br>• Specification of one-stage models (where applicable) including how clustering of patients within studies was accounted for.<br>• Use of fixed or random effects models and any other model assumptions, such as proportional hazards.<br>• How (summary) survival curves were generated (where applicable).<br>• Methods for quantifying statistical heterogeneity (such as I^2 and t^2).	14-16, Box 1, Text S1

		• How studies providing IPD and not providing IPD were analysed together (where applicable).<br>• How missing data within the IPD were dealt with (where applicable).	
Exploration of variation in effects	A2	If applicable, describe any methods used to explore variation in effects by study or participant level characteristics (such as estimation of interactions between effect and covariates). State all participant-level characteristics that were analysed as potential effect modifiers, and whether these were pre-specified.	14-16, Box 1, Text S1
Risk of bias across studies	15	Specify any assessment of risk of bias relating to the accumulated body of evidence, including any pertaining to not obtaining IPD for particular studies, outcomes or other variables.	15
Additional analyses	16	Describe methods of any additional analyses, including sensitivity analyses. State which of these were pre-specified.	14-16, Text S1
**Results**			
Study selection and IPD obtained	17	Give numbers of studies screened, assessed for eligibility, and included in the systematic review with reasons for exclusions at each stage. Indicate the number of studies and participants for which IPD were sought and for which IPD were obtained. For those studies where IPD were not available, give the numbers of studies and participants for which aggregate data were available. Report reasons for non-availability of IPD. Include a flow diagram.	18, Figure 1
Study characteristics	18	For each study, present information on key study and participant characteristics (such as description of interventions, numbers of participants, demographic data, unavailability of outcomes, funding source, and if applicable duration of follow-up). Provide (main) citations for each study. Where applicable, also report similar study characteristics for any studies not providing IPD.	Tables S1-S3
IPD integrity	A3	Report any important issues identified in checking IPD or state that there were none.	18
Risk of bias within studies	19	Present data on risk of bias assessments. If applicable, describe whether data checking led to the up-weighting or down-weighting of these assessments. Consider how any potential bias impacts on the robustness of meta-analysis conclusions.	Tables S6-S7
Results of individual studies	20	For each comparison and for each main outcome (benefit or harm), for each individual study report the number of eligible participants for which data were obtained and show simple summary data for each intervention group (including, where applicable, the number of events), effect estimates and confidence intervals. These may be tabulated or included on a forest plot.	18-21, Appendix pp 23-26, 30, 36, 42, 45, 49, 58
Results of syntheses	21	Present summary effects for each meta-analysis undertaken, including confidence intervals and measures of statistical heterogeneity. State whether the analysis was pre-specified, and report the numbers of studies and participants and, where applicable, the number of events on which it is based.	18-21
		When exploring variation in effects due to patient or study characteristics, present summary interaction estimates for each characteristic examined, including confidence intervals and measures of statistical heterogeneity. State whether the analysis was pre-specified. State whether any interaction is consistent across trials.	
		Provide a description of the direction and size of effect in terms meaningful to those who would put findings into practice.	

Risk of bias across studies	22	Present results of any assessment of risk of bias relating to the accumulated body of evidence, including any pertaining to the availability and representativeness of available studies, outcomes or other variables.	18, Tables S6-S8
Additional analyses	23	Give results of any additional analyses (e.g. sensitivity analyses). If applicable, this should also include any analyses that incorporate aggregate data for studies that do not have IPD. If applicable, summarise the main meta-analysis results following the inclusion or exclusion of studies for which IPD were not available.	18-21, Appendix pp 32-34, 38-44, 46-47, 51-57
Discussion			
Summary of evidence	24	Summarise the main findings, including the strength of evidence for each main outcome.	22
Strengths and limitations	25	Discuss any important strengths and limitations of the evidence including the benefits of access to IPD and any limitations arising from IPD that were not available.	22-25
Conclusions	26	Provide a general interpretation of the findings in the context of other evidence.	22-25
Implications	A4	Consider relevance to key groups (such as policy makers, service providers and service users). Consider implications for future research.	24-25
Funding			
Funding	27	Describe sources of funding and other support (such as supply of IPD), and the role in the systematic review of those providing such support.	26-27

© Reproduced with permission of the PRISMA IPD Group, which encourages sharing and reuse for non-commercial purposes

Text S1. Supplementary methods

Systematic review

All prospective *P. vivax* antimalarial clinical trials published between Jan 1, 2000 and July 26, 2024 were identified by the application of the key terms through Medline (Pubmed), Web of Science, Embase, Scopus and Cochrane Central. Abstracts of all references containing any mention of antimalarial drugs were manually checked to confirm prospective clinical trials, with review of full text when needed. This review was based on an original process that is documented in detail in Commons *et al*, Int J Parasitol Drug Drug Res 2017.[6] The year of the study was taken as the year of publication, although the start and end dates of patient enrolment were also recorded.

The following search terms were used in Pubmed: vivax AND (artefenomel OR arterolane OR amodiaquine OR atovaquone OR artemisinin OR arteether OR artesunate OR artemether OR artemotil OR azithromycin OR artekin OR chloroquine OR chlorproguanil OR cycloguanil OR clindamycin OR coartem OR dapsone OR dihydroartemisinin OR duo-cotecxin OR doxycycline OR halofantrine OR lumefantrine OR lariam OR malarone OR mefloquine OR naphthoquine OR naphthoquinone OR piperaquine OR primaquine OR proguanil OR pyrimethamine OR pyronaridine OR proguanil OR quinidine OR quinine OR riamet OR sulphadoxine OR tetracycline OR tafenoquine); filtered for publication from Jan 1, 2000 to July 26, 2024.

Inclusion and exclusion criteria

Studies were included if they:

- had active follow up of 28 days or more,
- included at least one patient aged <15 years,
- included a treatment group with daily primaquine given over multiple days where primaquine was commenced within 7 days of schizontocidal treatment and was co-administered with a schizontocidal antimalarial.

Studies on prevention, prophylaxis, reviews, animal studies, patients with severe malaria, where schizontocidal treatment was unsupervised or where data were extracted retrospectively from medical records outside of a planned trial were excluded.

Patients were excluded if

- they were ≥15 years old
- they had severe malaria on enrolment including haemoglobin <5 g/dL or haematocrit <15%,
- received adjunctive antimalarials during the first 14 days,
- were treated with an alternative hypnozoitocidal agent,
- received primaquine more than 7 days after starting antimalarial treatment,
- had a protocol violation in the original study, or
- were missing age, sex, or primaquine dose.

Inclusion in the efficacy meta-analysis was further restricted to:

- studies with 42 days or more follow up and
- patients with data on day 0 parasitaemia.

Inclusion in the gastrointestinal tolerability meta-analysis was further restricted to:

- studies with data from pre-specified symptom questionnaires (symptom checklist),
- patients with data on vivax parasite count at baseline,
- patients starting primaquine by day 2,

- patients not receiving intermittent primaquine (defined as primaquine administered weekly or monthly, rather than daily) and
- patients with data on daily primaquine dose.

Inclusion in the haematological safety meta-analysis was further restricted to:

- patients with available data on day 0 haemoglobin levels,
- patients with an available haemoglobin measurement on at least one more day during the follow-up period,
- patients starting primaquine by day 2 and
- patients with data on daily primaquine dose.

Inclusion in the adverse event meta-analysis was further restricted to:

- studies that shared data on adverse events in patients with confirmed *P. vivax* infection.

Definitions

The primaquine dose administered was calculated from the number of tablets administered to each patient. If the daily tablet counts were not available, doses were back-calculated using the dosing scheme available from study protocols, or if this was not available doses were assumed according to the planned dosing regimen.

Anaemia was defined as mild (Hb ≥8 g/dL and <11g/dl), moderate anaemia (Hb ≥5 g/dL and Hb <8g/dl) or severe anaemia (Hb <5g/dL).

G6PD deficiency was classified as severely deficient (<30% activity or a deficient qualitative test) or normal (≥30% activity or a normal qualitative test). Patients were also categorised into: severely deficient (<30% activity or a deficient qualitative test), intermediate deficiency (≥30% to <70% activity) or normal (≥70% activity).

The nutritional status of children aged <5 years of age was calculated as a weight-for-age z-score, using the igrowup package developed by WHO.[47] Those with weight-for-age z-scores <-2 (i.e. below the 3rd centile) were classified as underweight-for-age (termed underweight). Weight-for-age Z scores were set to missing if the score is less than -6 or greater than 6.

Transmission intensity of study sites was classified as low, moderate and high based on the malaria incidence estimates obtained for subnational regions and year from the Malaria Atlas Project.[48] Relapse periodicity (the time between vivax relapses) was classified as *low (long) and high (short)* according to Battle's regions, with high periodicity considered to include regions where the median periodicity is <47 days.[49]

CYP2D6 status was classified by expected phenotype using the activity score (AS) system[50,51] to estimate phenotype from genotype. The activity score assigns values of 0 to 2 to the CYP2D6 alleles identified in the patient as follows: zero, no-function alleles (*4, *4xN, *5); 0.25, substantially decreased-function (*10); 0.5, decreased-function (*9, *17, *29, *41); 1, normal-function (*1, *2, *39) and 2, increased function (*1xN, *2xN). The AS of diplotypes results from the sum of the assigned value to each allele. Patients with AS = 0 are designated as poor metabolisers. Patients with AS = 0.25, 0.5, 0.75 and 1 were designated as intermediate metabolisers. Patients with AS >2.25 were designated as ultrarapid metabolisers, respectively. Patients with AS = 1.25, 1.5, 2 and 2.25 were designated as normal metabolisers.[52]

South American studies that treat patients for 7 to 9 days depending on their bodyweight were considered with the 7-day treatment regimens. Primaquine treatment supervision were classified

as supervised if all doses were observed, partially supervised if ≥2 doses were observed or unsupervised if 0 or 1 dose was observed.

Gastrointestinal tolerability was assessed primarily on days 5-7 to reduce confounding related to symptomatic malaria or schizontocidal treatment. Symptoms on days 1-2 were also presented to allow comparison during the initial therapeutic response.

Additional outcome definitions and justification

Adverse events and serious adverse events were considered as such according to the primary studies' definitions, assumed to be the following unless otherwise indicated:

- Adverse events: Any untoward medical occurrence in a patient or clinical investigation subject administered a pharmaceutical product and which does not necessarily have a causal relationship with this treatment. An adverse event can therefore be any unfavourable and unintended sign (including an abnormal laboratory finding), symptom, or disease temporally associated with the use of a medicinal (investigational) product, whether or not related to the medicinal (investigational) product (ICH E6).
- Serious adverse events: A serious adverse event (experience) is any untoward medical occurrence that results in death or is life-threatening, requires inpatient hospitalisation or prolongation of existing hospitalisation; results in persistent or significant disability/incapacity or requires intervention to prevent permanent impairment or damage; is a congenital anomaly/birth defect; or other serious, important medical events.
  The term "life-threatening" in the definition of "serious" adverse event, refers to an event in which the patient was at risk of death at the time of the event (it does not refer to an event that hypothetically might have caused death if it were more severe) (ICH E2A).
  Medical and scientific judgement should be exercised in deciding whether expedited reporting is appropriate in other situations, such as important medical events that may not be immediately life-threatening or result in death or hospitalisation, but may jeopardise the patient or may require intervention to prevent one of the other outcomes listed in the definition above; these are also usually considered serious. Examples of such events are intensive treatment in an emergency room or at home for allergic bronchospasm; blood dyscrasias or convulsions that do not result in hospitalisation; or development of drug dependency or drug abuse (ICH E2A).

Grading classification and causality assessment of adverse events were as assessed by the primary study, and standardised as mild (grade 1), moderate (grade 2), severe (grade 3) and life-threatening (grade 4).

Only adverse events known to occur after starting primaquine (or equivalent date in control groups) were considered (i.e., treatment-emergent). If the day of adverse event onset was missing, or time of onset in relation to the primaquine dose was not recorded, the investigator was contacted for clarification, whenever possible.

Analysis

Efficacy analysis

For the Kaplan-Meier analysis for cumulative incidence of recurrence between day 7 and day of outcome, patients were left censored (origin) at day 7 and right censored (end time) at the first of: the day of recurrent malaria parasitaemia, the day prior to a >60 day blood smear gap, the day last reviewed, the last day of study follow up or the day of outcome.

In the Cox regression analyses body weight was excluded due to collinearity with age. Relapse periodicity, geographical region and transmission intensity were not included due to their

collinearity and the expectation that they would not satisfy the proportional hazards assumption due to differing time to first relapse in differing regions. Baseline haemoglobin and temperature were not included due to less complete data and no expected impact on the exposure of interest. Schizontocidal treatment was not included due to collinearity with study site, which was the clustering variable. The proportional hazards assumption was checked by visual inspection of the log-log plot (-$\log_e$[-$\log_e$(survival probability)] plotted against $\log_e$(analysis time)). The appropriateness of the assumption of linearity of the association between the outcome and the continuous covariates was confirmed using fractional polynomials. Additional subgroup analyses were undertaken sub-categorising childhood age into <5 years and 5 to <15 years. The <1 year old children were included in the <5 year category due to small numbers of patients in this age group.

Sensitivity analyses were undertaken restricting the outcome to recurrences between day 28 and 180, restricting the analysis to patients where the actual dose of primaquine administered was known, restricted to patients treated with chloroquine, restricted to studies with at least 180 days follow up, restricted to studies with treatment groups comparing primaquine to no primaquine, without shared frailty for study site and by removal of one study site at a time to determine the coefficient of variation.

To investigate the relationship between the continuous mg/kg dose of primaquine and the predicted rate of recurrence between day 7 and day 180, a restricted cubic spline model was used with four knots at default locations (5th, 35th, 65th and 95th percentiles).

Gastrointestinal tolerability analysis
Generalised estimating equations modified Poisson models were used to assess the association between primaquine daily dose and the presence of the composite outcome on days 5-7 or days 1-2, adjusted for age, sex and ($\log_{10}$) baseline parasite density with a log link, clustering by study site, exchangeable correlation structure and cluster robust error estimates. From each model the covariate-adjusted percentage of patients reporting the outcome was estimated at the mean values of the covariates. The linearity of the relationship between the quantitative variables (age and parasitaemia) and the outcome variable was assessed using fractional polynomial regression models, as well as the impact on model estimates of the effect of primaquine dosing on the outcome when adjusting for these confounders assuming either a linear or nonlinear association.

The risk of bias in the gastrointestinal tolerability analysis due to missing data is expected to be low based on the small proportion of patients with missing data for the confounders.

The risk of the composite gastrointestinal outcome (95% Confidence Intervals (CIs)) was calculated as the proportion of the total number of patients asked about the symptoms vomiting, diarrhoea or anorexia as part of a symptom checklist on any day between days 5-7. The 95% confidence intervals were estimated as Agresti-Coull confidence intervals.

The risk of acute vomiting was calculated as the number of patients reported to have vomited within an hour of a primaquine dose on days 0-2 and on days 3-13 as a proportion of the patients for whom these data were collected. The risks were stratified by primaquine daily dose categories and age categories (<5 years, 5 to <15 years).

Sensitivity analyses were performed using generalised estimating equations (modified Poisson models) as specified above, but with data restricted as specified for each analysis. In analyses where the modified Poisson regression models did not converge, mixed effects logistic regression models were fitted. All models adjusted for age, sex, and ($\log_{10}$) baseline parasite density, with

clustering by study site, and subsequently, covariate adjusted percentages were estimated at the means values of the covariates.

Haematological safety analysis

The percentage of patients experiencing each of the primary and secondary outcomes was calculated as the number of patients experiencing the outcome as a percentage of the total number of patients for whom data on haemoglobin were available in the time frame being examined.

In a post hoc analysis, the risk of anaemia by primaquine daily dose category was estimated using generalised estimating equations modified Poisson models, adjusting for age, sex and (log_{10}) baseline parasite density and baseline haemoglobin, with a log link, clustering by study site, exchangeable correlation structure and cluster robust error estimates. The covariate-adjusted percentage of patients reporting the outcome was estimated at the mean values of the covariates.

Adverse event analysis

The percentage of patients (95% CI) with the following events within 28 days after the first primaquine administration are presented by primaquine dose and age-category subgroups: serious adverse events, any adverse event, haemoglobinuria, anaemia, leucopoenia, methaemoglobinaemia, vomiting, anorexia, diarrhoea, abdominal pain, QT prolongation (data permitting).

A listing of adverse events by MedDRA System Organ Class and Preferred Term were described, detailing daily and total primaquine dose by day of adverse event, adverse event day in relation to primaquine initiation, and adverse event severity, relatedness and outcome, age/sex, schizontocidal drug, G6PD status and other relevant variables.

Figure S1. Directed acyclic graphs for the relationship between primaquine dose (exposure) and outcomes in children <15 years

A

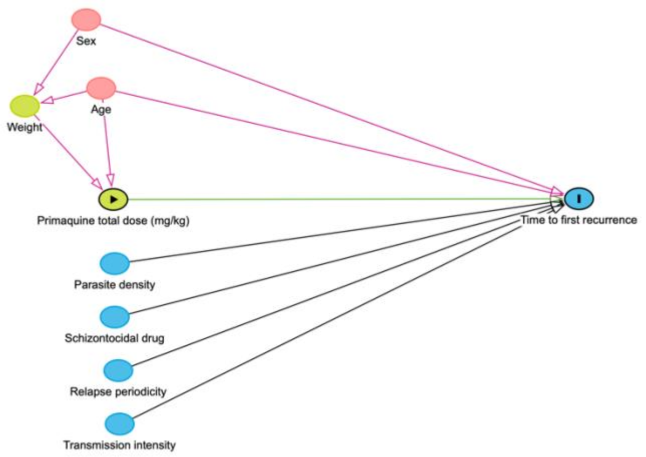

B

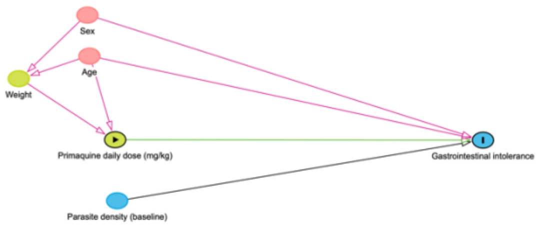

C

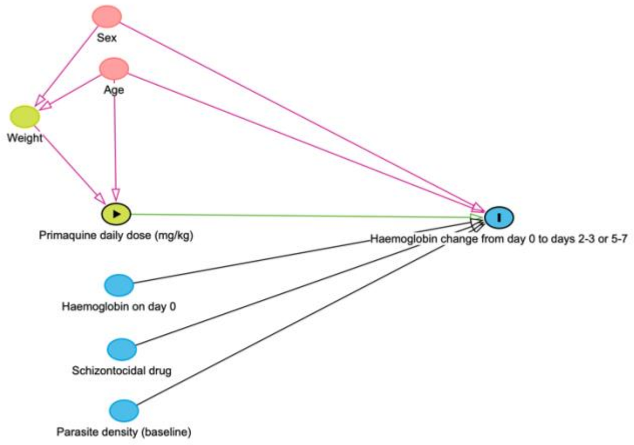

Green circles with triangles show exposures, blue circles with 'I' show outcomes, red circles show confounders and plain blue circles show other prognostic variables (A) The relationship between total primaquine dose in mg/kg and time to first *P. vivax* recurrence; (B) The relationship between daily primaquine dose in mg/kg and gastrointestinal intolerance and (C) The relationship between daily primaquine dose in mg/kg and haemoglobin on days 2-3 or days 5-7.

Table S1. Studies included in analysis

Author-year	Country	Recruitment Period	Age range	Follow up (days)	Included treatment groups*	PQ supervision	Randomised	Patients available (children)	Included in GI tolerability analysis	Included in adverse event analysis	Included in haematology analysis	Included in efficacy analysis
Hasugian-2007[15]	Indonesia	2005	1-56	84	AsAq_Pq_4.2_14d_D2, Dp_Pq_4.2_14d_D2	No	Yes	75	Yes	No	Yes	Yes
Leslie-2008[16]	Pakistan	2004 – 2006	4-80	330	Cq, Cq_Pq_7.0_14d_D0	Full	Yes	154	No	No	Yes	No
Abdallah-2012[28]	Sudan	2011	4-60	28	Al_Pq_4.2_14d_D0	No	No	12	Yes	No	No	No
Barber-2013[29]	Malaysia	2010 - 2015	13-62	42	Cq/ACT +/- Pq	No	No	3	Yes	No	Yes	Yes
Pasaribu-2013[17]	Indonesia	2010 - 2012	2-70	365	AsAq_Pq_3.5_14d_D0, Dp_Pq_3.5_14d_D0	Full	Yes	174	Yes	No	Yes	Yes
Marques-2014[30]	Brazil	2007 – 2008	13-65	28	Cq_Pq_3.5_7-9d_D0	Full	No	4	No	No	Yes	No
Gomes-2015[31]	Brazil	2011	10-56	28	Cq_Pq_3.5_7-12d_D0	Full	No	10	No	No	No	No
Gonzalez-Ceron-2015[18]	Mexico	2008 - 2010	3-78	365	Cq_Pq_3.5_14d_D0	Full	Yes	45	Yes	No	No	Yes
Thanh-2015[32]	Vietnam	2009 – 2010	3-60	28	Cq_Pq_5.0_10d_D0	Full	No	160	Yes	No	Yes	No
Yuan-2015[33]	Myanmar	2012 - 2013	1-77	42	Cq_Pq_3.0_8d_D0	Partial	No	437	No	No	No	Yes
Ley-2016[34]	Bangladesh	2014 – 2015	1-66	30	Cq_Pq_3.5_14d_D2	No	No	27	No	No	Yes	No
Longley-2016[35]	Thailand	2014	7-71	270	Cq_Pq_3.5_14d_D1	Full	No	17	No	No	No	Yes
Zuluaga-Idarraga-2016[36]	Colombia	2012 - 2013	4-71	180	Cq_Pq_3.5_14d_D0	Full	No	18	Yes	No	No	Yes
Abreha-2017[19]	Ethiopia	2012 - 2014	1-67	365	Al, Al_Pq_3.5_14d_D2, Cq, Cq_Pq_3.5_14d_D2	Partial	Yes	156	Yes	Yes	Yes	Yes
Awab-2017[20]	Afghanistan	2009 - 2013	2-84	390	Cq, Cq_Pq_3.5_14d_D0	Partial	Yes	257	Yes	Yes	Yes	Yes
Brasil-2018[37]	Brazil	2012 – 2014	9m-92	180	Cq_Pq_3.5_7d_D0	No	No	67	No	No	No	No
Chu-2018[21]	Thailand	2010 - 2011	1-63	365	Cq, Cq_Pq_7.0_14d_D0	Full	Yes	211	No	Yes	Yes	Yes
Grigg-2018[38]	Malaysia	2013 - 2015	8m-65	230	Cq/ACT +/- Pq	No	No	19	No	No	Yes	Yes
Hamid-2018[22]	Sudan	2015-2016	2-70	42	AsSP_Pq_3.5_14d_D2, AsSP	No	Yes	32	Yes	Yes	Yes	Yes
Chu-2019[23]	Thailand	2012 - 2014	1-63	365	Cq_Pq_7.0_14d_D0, Cq_Pq_7.0_7d_D0, Dp_Pq_7.0_14d_D0, Dp_Pq_7.0_7d_D0	Full	Yes	206	No	Yes	Yes	Yes
de Sena-2019[39]	Brazil	2016 - 2017	2-14	42	Cq_Pq_3.5_7d_D0	Partial	No	113	No	No	No	Yes
Ladeia-Andrade-2019[24]	Brazil	2014 - 2015	7-60	180	Cq_Pq_3.5_7d_D0	Full	Yes	24	Yes	No	Yes	Yes
Rijal-2019[25]	Nepal	2015 - 2016	5-75	365	Cq, Cq_Pq_3.5_14d_D0	Partial	Yes	13	Yes	No	Yes	Yes
Taylor-2019[26]	Multinational	2014 - 2017	9m-94	365	Cq, Cq_Pq_7.0_14d_D0, Cq_Pq_7.0_7d_D0, Dp, Dp_Pq_7.0_14d_D0, Dp_Pq_7.0_7d_D0	Full	Yes	1013	Yes	Yes	Yes	Yes
Xu-2020[40]	Myanmar	2014 – 2016	3-59	28	Cq_Pq_3.5_14d_D0	Full	No	150	No	No	No	No
Poespoprodjo-2022[27]	Indonesia	2016-2018	1-63	180	Dp_Pq_7.0_14d_D2 (partially supervised), Dp_Pq_7.0_14d_D2 (unsupervised)	Partial/No	Cluster randomised	88	Yes	No	Yes	Yes
Karunajeewa-unpublished	Vanuatu	2013	2-35	84	Al, Al_Pq_3.5_14d_D0, Al_Pq_7.0_14d_D0	Full	Yes	29	Yes	No	Yes	Yes

ACT – artemisinin-based combination treatment; As – artesunate; Al – artemether-lumefantrine; Aq – amodiaquine; Cq – chloroquine; Dp – dihydroartemisinin-piperaquine; GI – gastrointestinal; Mf – mefloquine; PQ/Pq – primaquine; SP – sulfadoxine-pyrimethamine; *Treatment code describes (schizontocidal drug)_(hypnozoitocidal drug)_(total primaquine dose)_(duration of primaquine treatment eg 14d = 14 days)_(primaquine start day)

Table S2. Study testing and inclusion criteria

Author-year	Hb exclusion criteria	G6PD testing undertaken	G6PD activity included	G6PD data available	Days symptom checklist undertaken and data available	Days Hb/Hct measured and data available	CYP2D6 data available	Haemoglobinuria assessment	Anaemia definition	Adverse event elicitation method
Hasugian-2007[15]	Hct <15%	FST	≥30% activity	Yes	0, 1, 2, 7, 14	0, 7	No			
Leslie-2008[16]	Hb <7 g/dL	Colorimetric test	All activities	Yes	-	0, 7, 14	No			
Abdallah-2012[28]	Not stated	No	All activities	No	0	-	No			
Barber-2013[29]	No exclusions	FST	≥30% activity	Yes	0	0, 1, 2, 3	No			
Pasaribu-2013[17]	Hb <5 g/dL	Only in patients with haemolysis at study end	All activities	No	0, variable	0, 2	No			
Marques-2014[30]	No exclusions	Not stated	Unknown	No	-	0, 3, 7, 14	No			
Gomes-2015[31]	Not stated	Not stated	Unknown	No	-	0	No			
Gonzalez-Ceron-2015[18]	Hb <5 g/dL	No	All activities	No	0, 2, 3, 7	-	No			
Thanh-2015[32]	Not stated	No	All activities	No	0, 1, 2, 3, 7, 14	0, 14	No			
Yuan-2015[33]	Not stated	No	All activities	No	-	-	No			
Ley-2016[34]	Hb <8 g/dL	Spectrophotometry	All activities (if deficient not given PQ)	Yes	-	0, 2, 9	No			
Longley-2016[35]	Not stated	Yes; type not stated	≥30% activity	Yes	-	-	Yes			
Zuluaga-Idarraga-2016[36]	Hb <5 g/dL	No	All activities	No	0, 1, 2, 3, 7, 13	-	No			
Abreha-2017[19]	Hb <8 g/dL	FST	≥30% activity	Yes	0, 1, 2, 3, 7, 14	0, 3, 7, 14	No	Routine question re colour of urine	Hb <8 g/dL	Solicited and unsolicited
Awab-2017[20]	Hb <8 g/dL	FST	≥30% activity	Yes	0, 1, 2, 7	0, 1, 2, 7, 14	No	Patient informed to report dark urine	Hb <8 g/dL	Checklists and open question
Brasil-2018[37]	Not stated	Not stated	Unknown	No	-	-	Yes			
Chu-2018[21]	Hct <25%	FST	All activities (if deficient not given PQ)	Yes	-	0	No	Not done	Haematocrit <30% (<33% if <2 years old)	Observed or reported
Grigg-2018[38]	Hb <5 g/dL	FST	All activities (if deficient not given PQ)	Yes	-	0, 1, 2, 7, 14	No			
Hamid-2018[22]	Hb <8 g/dL	At 1 of 2 sites; Spectrophotometry & Biosensor (AccessBio)	≥30% activity	Yes	-	0, 1, 2, 7, 14	No	Routine question on colour of urine (very dark or not)	Hb <10 g/dL	Checklist
Chu-2019[23]	Hct <25%	FST	≥30% activity	Yes	-	0, 1, 2, 3, 4, 5, 6, 7, 14	No	Not done	Haematocrit <30%	Observed or reported
de Sena-2019[39]	Hb <5 g/dL	Not stated	Unknown	No	-	0	No			
Ladeia-Andrade-2019[24]	Hb <8 g/dL	BinaxNow RDT	≥30% activity	Yes	0, 1, 2, 3, 7, 14	0, 1, 2, 3, 7, 14	Yes			
Rijal-2019[25]	Not stated	Carestart RDT	≥30% activity	Yes	0	0, 1, 3, 7	No			
Taylor-2019[26]	Hb <9 g/dL	FST	≥30% activity	Yes	0, 1, 2, 3, 4, 5, 6, 7, 8, 9, 10, 11, 12, 13	0, 3, 7, 13	No	Hillmen ≥5	Hb <10 g/dL	Checklists (Grade 3 or 4 AEs only recorded)

Xu-2020[40]	Not stated	No	All activities	No	-	-	No
Poespoprodjo-2022[27]	Hb <9 g/dL	FST	≧30% activity	Yes	0, 1, 2	0, 2	No
Karunajeewa-unpublished	Hb < 5 g/dL	Carestart or BinaxNow RDT	≧30% activity	Yes	0, 1	0, 1, 2, 3, 7, 10, 14	No

Table S3. Study sites included in analysis

Paper	Study site	Country	Region	Lat	Long	Year Start	Year End	MAP Incidence rate (per 1000 persons)	Transmission intensity*	Relapse periodicity#
Hasugian-2007[15]	Timika	Indonesia	Asia-Pacific	-4.50	136.85	2005	2005	22.61	High	High
Leslie-2008[16]	Adizai	Pakistan	Asia-Pacific	33.79	71.58	2004	2006	2.48	Moderate	Low
Leslie-2008[16]	Baghicha	Pakistan	Asia-Pacific	34.23	72.16	2004	2006	2.48	Moderate	Low
Leslie-2008[16]	Khagan	Pakistan	Asia-Pacific	34.54	73.32	2004	2006	2.48	Moderate	Low
Abdallah-2012[28]	Kassala	Sudan	Africa	15.41	36.42	2011	2011	6.35	Moderate	Low
Barber-2013[29]	Sabah	Malaysia	Asia-Pacific	5.98	116.08	2011	2011	0.21	Low	High
Pasaribu-2013[17]	Tanjung Leidong	Indonesia	Asia-Pacific	2.77	99.98	2011	2011	2.75	Moderate	High
Marques-2014[30]	Manaus	Brazil	Americas	-3.11	-60.03	2007	2008	73.17	High	Low
Gomes-2015[31]	Oiapoque-AP	Brazil	Americas	3.84	-51.83	2011	2011	37.70	High	Low
Gonzalez-Ceron-2015[18]	Huehuetan	Mexico	Americas	15.06	-92.33	2008	2009	0.55	Low	Low
Gonzalez-Ceron-2015[18]	Tuxtla Chico	Mexico	Americas	14.91	-92.15	2008	2009	0.55	Low	Low
Gonzalez-Ceron-2015[18]	Fr Hidalgo	Mexico	Americas	14.78	-92.18	2008	2009	0.55	Low	Low
Gonzalez-Ceron-2015[18]	Cacahoatan	Mexico	Americas	15.09	-92.21	2008	2009	0.55	Low	Low
Gonzalez-Ceron-2015[18]	Tapachula	Mexico	Americas	14.91	-92.26	2008	2009	0.55	Low	Low
Thanh-2015[32]	Tra Leng	Vietnam	Asia-Pacific	24.75	97.55	2009	2011	0.15	Low	High
Yuan-2015[33]	Laiza	Myanmar	Asia-Pacific	24.75	97.55	2013	2013	9.41	Moderate	High
Ley-2016[34]	Alikadam	Bangladesh	Asia-Pacific	21.65	92.31	2014	2015	0.59	Low	High
Longley-2016[35]	Tha Song Yang	Thailand	Asia-Pacific	17.57	97.92	2014	2014	3.09	Moderate	High
Zuluaga-Idarraga-2016[36]	Turbo	Colombia	Americas	8.10	-76.73	2012	2012	5.08	Moderate	Low
Abreha-2017[19]	Batu	Ethiopia	Africa	6.67	39.42	2013	2013	85.49	High	Low
Abreha-2017[19]	Bishoftu	Ethiopia	Africa	8.73	39.01	2013	2013	85.49	High	Low
Awab-2017[20]	Jalalabad	Afghanistan	Asia-Pacific	34.43	70.46	2009	2014	40.12	High	Low
Brasil-2018[37]	Manaus	Brazil	Americas	-3.11	-60.02	2012	2014	35.04	High	Low
Chu-2018[21]	Mae Sot	Thailand	Asia-Pacific	16.72	98.58	2010	2010	2.15	Moderate	High
Grigg-2018[38]	Kudat	Malaysia	Asia-Pacific	6.89	116.85	2014	2014	0.11	Low	High
Hamid-2018[22]	Khartoum	Sudan	Africa	15.51	32.54	2015	2016	6.78	Moderate	Low
Hamid-2018[22]	New Halfa	Sudan	Africa	15.33	35.60	2015	2016	2.70	Moderate	Low
Chu-2019[23]	Mae Sot	Thailand	Asia-Pacific	16.72	98.58	2014	2014	3.09	Moderate	High
de Sena-2019[39]	Marajo Island	Brazil	Americas	-0.94	-49.64	2016	2017	5.89	Moderate	Low
Ladeia-Andrade-2019[24]	Mancio Lima	Brazil	Americas	-7.61	-72.91	2014	2014	47.40	High	Low
Rijal-2019[25]	Jhapa	Nepal	Asia-Pacific	26.55	87.89	2016	2016	0.12	Low	High
Rijal-2019[25]	Kailali	Nepal	Asia-Pacific	28.83	80.90	2016	2016	0.22	Low	High
Taylor-2019[26]	Krong Pa	Vietnam	Asia-Pacific	13.22	108.67	2015	2017	0.18	Low	High
Taylor-2019[26]	Dak O	Vietnam	Asia-Pacific	12.00	107.50	2015	2017	0.24	Low	High
Taylor-2019[26]	Bu Gia Map	Vietnam	Asia-Pacific	12.04	107.05	2015	2017	0.24	Low	High
Taylor-2019[26]	Hanura	Indonesia	Asia-Pacific	-5.53	105.24	2015	2017	1.01	Moderate	High
Taylor-2019[26]	Tanjung Leidong	Indonesia	Asia-Pacific	2.77	99.98	2015	2017	1.03	Moderate	High

Taylor-2019[26]	Arba Minch	Ethiopia	Africa	6.01	37.54	2015	2017	17.80	High	Low
Taylor-2019[26]	Laghman	Afghanistan	Asia-Pacific	34.70	70.15	2015	2017	97.70	High	Low
Taylor-2019[26]	Metahara	Ethiopia	Africa	8.90	39.92	2015	2017	26.02	High	Low
Taylor-2019[26]	Jalalabad	Afghanistan	Asia-Pacific	34.43	70.46	2015	2017	116.49	High	Low
Xu-2020[40]	Laiza	Myanmar	Asia-Pacific	24.75	97.55	2014	2016	6.67	Moderate	High
Poespoprodjo-2022[27]	Papua	Indonesia	Asia-Pacific	-4.24	136.82	2016	2018	18.40	High	High
Karunajeewa-unpublished	Nambauk	Vanuatu	Asia-Pacific	-15.45	167.08	2013	2013	24.58	High	High
Karunajeewa- unpublished	Port Olry	Vanuatu	Asia-Pacific	-15.04	167.07	2013	2013	24.58	High	High
Karunajeewa- unpublished	Luganville	Vanuatu	Asia-Pacific	-15.51	167.20	2013	2013	24.58	High	High

Lat – latitude; Long – longitude; MAP – malaria Atlas Project; *Transmission intensity is classified as low (an incidence rate of <1 per 1000 persons), moderate (1 to <10 per 1000 persons), high (≥10 per 1000 persons); # High relapse periodicity <47 days.

Table S4. Reasons for studies not being included in analyses

Reason	Number of studies	Studies
Data not available by 30 June 2022	9	[45,53-64]
Investigators unable to be contacted	2	[65,66]
Missing minimum data for inclusion	2	[67,68]
Initial investigator response but no data provided	4	[69-72]
No response from investigators	21	[73-93]

Table S5. Studies eligible for the analyses but not included

First Author	Treatment groups	Region	Country	Follow up (days)	Randomised	Recruitment period	Treatment groups	Pv patients enrolled	Treated with PQ	Female (%)	Mean Age (SD)	Median Age (range)	Reasons for exclusion	Eligibility			
														Efficacy	GI tol	Haem	Adverse events
Villalobos-Salcedo-2000[65]	2	Americas	Brazil	28	Yes	1998	Cq_Pq_3.5_14d_D3; Cq_Pq_2.5_5d_D0	79	79	21.5	31.7 (-)		Unable to contact	No	No	No	Not stated
Abdon-2001[73]	3	Americas	Brazil	180	Yes	1994-1995	Cq_Pq_3.5_14d_D0; Cq_Pq_3.5_7d_D0; Cq_Pq_2.5_5d_D0	120	120	37.5	27.3 (-)		No response	Yes	No	No	Not stated
Buchachart-2001[74]	1	Asia-Pacific	Thailand	28	No	1992-1997	Cq_Pq_3.5_14d_D3	593	593	37.1	25.1 (-)		No response	No	No	No	Yes
Solari Soto-2002[75]	2	Americas	Peru	60	Yes	1998-1999	Cq_Pq_3.5_7d_D2; Cq_Pq_3.5_14d_D2	60	60	43.3	26.45 (16.96)		No response	Yes	Yes	No	Yes
Yadav-2002[76]	2	Asia-Pacific	India	365	Yes	1988-1991	Cq; Cq_Pq_1.25_5d_D2	1482	759	Not stated	Not stated		No response	Yes	No	No	Not stated
Pinto-2003[77]	2	Americas	Brazil	28	Yes	1997-1998	Cq_Pq_3.0_14d_D0; Cq_Pq_3.0_14d_D0	132	132	37.9	30.7 (-)		No response	No	Not stated	No	Yes
Leslie-2004[67]	3	Asia-Pacific	Pakistan	270	Yes	2000-2001	Cq; Cq_Pq_3.5_14d_D0; Cq_Pq_3.5_14d_D0	595	383	50.8	12.9 (-)		Missing minimum data	Yes	No	No	Yes
Maguire-2006[78]	2	Asia-Pacific	Indonesia	28	Yes	1996-1999	Cq_Pq_3.5_14d_D0; Mf_Pq_3.5_14d_D0	243	243	35.2	22.6 (-)		No response	No	Not stated	No	Not stated
Krudsood-2008[79]	6	Asia-Pacific	Thailand	28	Yes	Not stated	As_Pq_2.5_5d_D5; As_Pq_3.5_7d_D5; As_Pq_4.5_9d_D5; As_Pq_5.5_11d_D5; As_Pq_7.0_7d_D5; As_Pq_7.0_7d_D5;	399	399	35.1	24.2 (-)		No response	No	No	No	Yes
Carmona-Fonseca-2009[53]	4	Americas	Colombia	120	Yes	2001-2003	Cq_Pq_3.5_14d_D1; Cq_Pq_1.75_3d_D1; Cq_Pq_2.5_3d_D1; Cq_Pq_3.5_3d_D1	188	188	30.4	Not stated		Data not available	Yes	No	No	Not stated
Nateghpour-2009[80]	1	Asia-Pacific	Iran	28	No	Not stated	Cq_Pq_6.0_8w_D3	Not available	Not available	Not available	Not available		No response	No	No	No	Not stated
Orjuela-Sanchez-2009[68]	2	Americas	Brazil	336	No	2004-2007	Cq_Pq_3.5_7d_D0; Cq_Pq_3.5_7d_D0	164	164	Not stated	Not stated		Missing minimum data	Yes	No	No	Not stated
Carmona-Fonseca-2010[54]	2	Americas	Colombia	120	Yes	2005-2008	Cq_Pq_3.5_7d_D1; Cq_Pq_3.5_3d_D1	79	79	Not stated	Not stated		Data not available	Yes	Yes	No	Yes
Takeuchi-2010[81]	2	Asia-Pacific	Thailand	90	Yes	2007-2009	Cq_Pq_3.5_14d_D3; Cq_Pq_3.5_14d_D3	216	216	39.8	Not stated		No response	Yes	No	No	Yes
Maneeboonyang-2011[82]	2	Asia-Pacific	Thailand	90	Yes	2005-2006	Cq_Pq_3.5_14d_D3; Cq_Pq_3.5_14d_D3	92	92	40	Not stated		No response	Yes	No	No	Not stated
Van Den Eede-2011[69]	1	Americas	Peru	365	No	2008	Cq_Pq_3.5_7d_D0	51	51	49	Not stated	15 (2-80)	Data not provided	Yes	Yes	No	Yes
Eibach-2012[66]	1	Americas	Guyana	28	No	2009-2010	Al_Pq_3.5_14d_D0	74	74	9.5	Not stated	24 (5-57)	Unable to contact	No	No	No	Not stated
Graf-2012[83]	3	Americas	Peru	210	Yes	2005-2008	Cq_Pq_2.5_5d_D0; Cq_Pq_3.5_7d_D0; Cq_Pq_3.5_14d_D0	540	540	Not stated	Not stated		No response	Yes	No	No	Not stated
Pedro-2012[84]	1	Americas	Brazil	28	No	2005-2011	Cq_Pq_3.5_Xd_D0	47	47	24.5	Not stated		No response	No	No	No	Yes
Ganguly-2013[85]	2	Asia-Pacific	India	42	Yes	2011-2012	Cq; Cq_Pq_3.5_14d_D0	250	125	10.8	25.2 (-)		No response	Yes	Yes	Yes	Yes
Liu-2013[86]	2	Asia-Pacific	China	365	Yes	2009-2010	Cq_Pq_4.0_8d_D0; Anq	260	128	14	Not stated		No response	Yes	Yes	Yes	Yes
Miahipour-2013[87]	2	Asia-Pacific	Iran	540	No	2008-2011	Cq; Cq_Pq_6.0_8w_D3	163	163	23.3	Not stated		No response	Yes	No	No	Not stated
Zhu-2013[88]	1	Asia-Pacific	China	28	No	2008-2009	Cq_Pq_4.0_8d_D3	39	39	42.1	43 (-)		No response	No	No	No	Yes
Delgado-Ratto-2014[89]	1	Americas	Peru	720	No	2008	Cq_Pq_3.5_7d_D0	37	37	48.6	Not stated	15	No response	Yes	Yes	No	Yes

Kheng-2015[55]	1	Asia-Pacific	Cambodia	56	No	2013-2014	Dp_Pq_6.0_8w_D0	75	75	16	Not stated	24 (5-63)	Data not available	Yes	No	No	Yes
Negreiros-2016[71]	1	Americas	Brazil	168	No	2014	Cq_Pq_3.5_7d_D0	119	119	45.4	Not stated	23.4 (5-67.3)	Data not provided	Yes	Yes	No	Yes
Valecha-2016[56]	2	Asia-Pacific	India	42	Yes	2011-2013	Cq_Pq_3.5_14d_D3; ArtmPip_Pq_3.5_14d_D3	317	317	8.2	33.7 (13.5)		Data not available	Yes	No	No	Yes
Mac Donald-Ottevanger-2017[90]	2	Americas	Suriname	365	Yes	2006-2008	Cq_Pq_3.5_7d_D3; Cq_Pq_3.5_14d_D3	79	79	34.4		24.64 (-)	No response	Yes	No	No	Not stated
Dharmawardena-2017[57]	1	Asia-Pacific	Sri Lanka	365	No	2015-2016	Cq_Pq_3.5_14d_D2	32	32	6.8		35.5 (13-66)	Data not available	Yes	No	No	Not stated
Azarian Moghadam-2018[91]	1	Asia-Pacific	Iran	28	No	2013-2015	Dp_Pq_6.0_8w_D2	170	170	35.3	Not stated		No response	No	No	No	Not stated
Pham-2019[72]	1	Asia-Pacific	Vietnam	730	No	2009-2011	Cq_Pq_5.0_10d_D0	260	260	39	Not stated		Data not provided	Yes	No	No	Not stated
Han-2020[92]	1	Asia-Pacific	Myanmar	28	No	2017-2019	AsPy_Pq_3.5_14d_D0	206	206	35	27.2		No response	No	No	No	Yes
Shaikh-2021[93]	1	Asia-Pacific	Pakistan	56	No	2018-2019	Al_Pq_6.0_8w_D0	40	40	0	Not stated		No response	Yes	No	No	Yes
Arcelia-2023[60]	1	Asia-Pacific	Indonesia	28	No	2019-2020	Dp_Pq_3.5_14d_D0	60	60	Not stated	Not stated		Data not available by June 30, 2022	No	Yes	No	Yes
Mekonnen-2023[58]	1	Africa	Ethiopia	42	No	2019-2020	Cq_Pq_3.5_14d_D0	102	102	50	Not stated	13.5	Data not available by June 30, 2022	Yes	Yes	Yes	Yes
Moore-2023[59]	3	Asia-Pacific	PNG	63	Yes	2013-2018	Al_Pq_7.0_14d_D0; Al_Pq_7.0_7d_D0; Al_Pq_7.0_3.5d_D0	73	73	40	Not stated	6.6	Data not available by June 30, 2022	Yes	No	Yes	Yes
Woon-2023[45]	2	Asia-Pacific	PNG	84	Yes	2018-2019	Al_Pq_7.0_3.5d_D3; Al_Pq_7.0_3.5d_D24	219	219	41	Not stated	6.6	Data not available by June 30, 2022	Yes	No	No	Yes
Malla-2023[61]	1	Asia-Pacific	Myanmar	365	No	2012-2013	Cq_Pq_3.5_14d_D0	556	556	Not stated	10.7 (-)		Data not available by June 30, 2022	Yes	No	No	No
Liu-2024[62]	2	Asia-Pacific	Myanmar	365	Yes	2017-2020	Anq_Pq_3.5_14d_D0; Anq_Pq_0.9_3.5d_D0;	307	307	43.4	Not stated	19 (-)	Data not available by June 30, 2022	Yes	No	No	Yes
Gebrie-2024[63]	1	Africa	Ethiopia	42	No	2022-2023	Cq_Pq_3.5_14d_D0	100	100	38	12 (-)		Data not available by June 30, 2022	Yes	Yes	Yes	Yes
Manh-2024[64]	1	Asia-Pacific	Vietnam	42	No	2018-2019	AsPy_Pq_3.5_14d_D0	59	59	11.9	36.2 (-)		Data not available by June 30, 2022	Yes	No	Yes	Yes

Al – artemether-lumefantrine; Anq – artemisinin-naphthoquine; Artm – arterolane maleate; As – artesunate; Bq – bulaquine; Cq – chloroquine; Dp – dihydroartemisinin-piperaquine; Mf – mefloquine; Pip – piperaquine; PQ/Pq – primaquine; Pv – *P. vivax*; Py – pyronaridine; SD – standard deviation; Tnd – tinidazole; Tq – tafenoquine; *Treatment code describes (schizontocidal drug)_(hypnozoitocidal drug)_(total primaquine dose)_(duration of primaquine treatment eg 14d = 14 days)_(primaquine start day)

Figure S2. Location of study sites for included studies

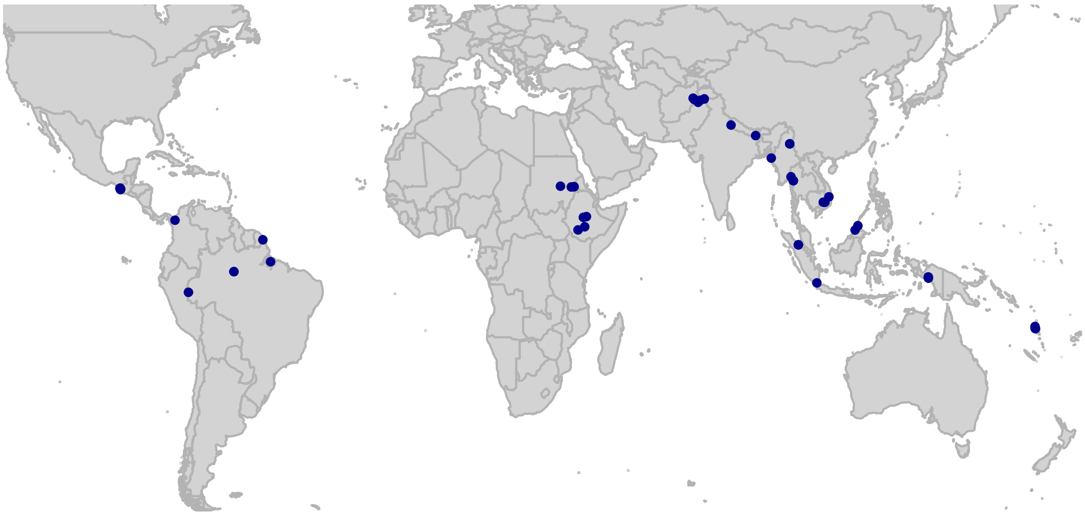

Table S6. Risk of bias assessment in randomised controlled studies

Author-year	Bias from randomisation	Bias due to deviation from intervention	Bias from missing outcome				Bias in measurement of the outcome				Bias in selection of the reported results	Overall bias	Balanced age groups	Follow up to 180 days	Comparison of no PQ to PQ
			Efficacy	Haematology	Tolerability	AEs	Efficacy	Haematology	Tolerability	AEs					
Hasugian-2007[15]	Green	Green	Orange	Green	Green	Grey	Green	Green	Orange	Grey	Orange	Orange	Green	Red	Red
Leslie-2008[16]	Orange	Green	Green	Green	Grey	Grey	Green	Green	Grey	Grey	Orange	Orange	Red	Grey	Green
Pasaribu-2013[17]	Green	Green	Orange	Green	Orange	Grey	Green	Green	Orange	Grey	Orange	Orange	Red	Green	Red
Gonzalez-Ceron-2015[18]	Red	Green *	Orange	Grey	Green	Grey	Green	Grey	Orange	Grey	Orange	Red	Red	Green	Red
Abreha-2017[19]	Green	Green	Orange	Green	Green	Orange	Green	Green	Orange	Orange	Orange	Orange	Red	Green	Green
Awab-2017[20]	Green	Green	Orange	Green	Green	Orange	Green	Green	Orange	Orange	Orange	Orange	Red	Green	Green
Chu-2018[21]	Green	Green	Orange	Green	Grey	Orange	Green	Green	Grey	Orange	Orange	Orange	Red	Green	Green
Hamid-2018[22]	Green	Green	Orange	Green	Grey	Orange	Green	Green	Grey	Orange	Orange	Orange	Red	Red	Green
Chu-2019[23]	Green	Green *	Orange	Grey	Grey	Orange	Green	Green	Grey	Green	Orange	Orange	Red	Green	Red
Ladeia-Andrade-2019[24]	Green	Green	Orange	Green	Green	Grey	Green	Green	Orange	Grey	Orange	Orange	Red	Green	Red
Rijal-2019[25]	Green	Green *	Orange	Green	Green	Grey	Green	Green	Orange	Grey	Orange	Orange	Red	Green	Green
Taylor-2019[26]	Green	Green	Green	Green	Green	Orange	Green	Green	Green	Green	Green	Orange	Red	Green	Green
Poespoprodjo-2022[27]	Green	Green	Green	Green	Green	Grey	Green	Green	Orange	Grey	Orange	Orange	Green	Green	Green
Karunajeewa-unpublished	Green	Green	Green	Green	Green	Grey	Green	Green	Orange	Grey	Orange	Orange	Green	Red	Green

Green – low risk of bias; Red – high risk of bias; Orange – unclear risk of bias; Grey – not applicable; Assessed according to the Cochrane Risk of Bias 2 tool[12] for randomised controlled trials, using the entire study population (children and adults); * Studies analysed per protocol but all data available for these meta-analyses; AEs – adverse events; PQ – primaquine.

Table S7. Risk of bias assessment in single arm observational studies

Author-year	Clear criteria for inclusion	Condition measured in reliable way	Valid methods for condition	Consecutive inclusion	Complete inclusion	Demographics reported	Clinical information reported	Outcomes reported	Site description	Analysis appropriate	Balanced age groups	Follow up to 180 days	Comparison of no PQ to PQ
Abdallah-2012[28]	Green	Green	Green	Green	Green	Green	Green	Green	Green	Green	Red	Grey	Grey
Barber-2013[29]	Green	Green	Green	Green	Green	Green	Green	Green	Green	Green	Red	Red	Grey
Marques-2014[30]	Green	Green	Green	Red	Grey	Green	Green	Green	Green	Green	Red	Grey	Grey
Gomes-2015[31]	Green	Green	Green	Green	Green	Green	Green	Green	Green	Green	Red	Grey	Grey
Thanh-2015[32]	Green	Green	Green	Green	Green	Green	Green	Green	Green	Green	Green	Grey	Grey
Yuan-2015[33]	Green	Green	Green	Green	Green	Green	Green	Green	Green	Green	Red	Red	Grey
Ley-2016[34]	Green	Green	Green	Green	Green	Green	Green	Green	Green	Green	Red	Grey	Grey
Longley-2016[35]	Green	Green	Green	Green	Green	Green	Green	Green	Green	Green	Red	Green	Grey
Zuluaga-Idarraga-2016[36]	Green	Green	Green	Green	Green	Green	Green	Green	Green	Green	Red	Green	Grey
Brasil-2018[37]	Green	Green	Green	Green	Green	Green	Green	Green	Green	Green	Green	Grey	Grey
Grigg-2018[38]	Orange	Green	Green	Orange	Orange	Red	Red	Green	Green	Green	Green	Red	Grey
de Sena-2019[39]	Green	Green	Green	Green	Green	Green	Green	Green	Green	Green	Red	Red	Grey
Xu-2020[40]	Green	Green	Green	Green	Green	Green	Green	Green	Green	Green	Red	Grey	Grey

Green – yes (low risk of bias); Red – no (higher risk of bias); Orange – unclear; Grey – not applicable; Assessed according to the Joanna Briggs Institute Case Series tool[13] for single arm studies, using the entire study population (children and adults); The appropriateness of analysis was considered appropriate for all studies given that the individual patient data were re-analysed as part of these meta-analyses; PQ – primaquine.

Of 21 studies not included in the adverse events analysis, 11 were not explicit about assessing adverse events and did not report any serious adverse events. Of the remaining ten, seven found no serious adverse events among a total of 1714 participants treated with primaquine, with Gonzalez-Ceron 2015 elaborating that 'some' patients had light jaundice, but none reported dark urine or any other clinical symptoms of haemolysis. Three studies reported serious adverse events. Hasugian 2007 found two adverse events among 334 participants treated with primaquine, although these were likely experienced prior to primaquine dosing. Both were adults with recurrent vomiting hospitalised on D3 after completion of artesunate plus amodiaquine, and made a full recovery. Pasaribu 2013 found one serious adverse events in 331 participants treated with artesunate or dihydroartemisinin-piperaquine and primaquine at 0.25 mg/kg for 14 days, a pericarditis 10 days after treatment, whereupon primaquine was discontinued and he made a full recovery. Poespoprodjo 2022 found two serious adverse events in 474 participants dosed with primaquine at 15 mg daily for 14 days (0.5mg/kg). One was a 56-year-old with acute falciparum malaria who suffered cerebral complications on day 1 and the other an 11-year-old boy, also with acute falciparum malaria who had intractable vomiting from day 1. Both events were deemed unrelated to primaquine, and the participants recovered.

Table S8. Comparison of baseline characteristics between included studies and eligible but not included studies

Characteristic	Included studies (n=27)	Studies eligible but not included (n=41)
Region[#]		
Asia-Pacific, studies (%)	17.75 (66%)	25 (61%)
Africa, studies (%)	2.25 (8%)	2 (5%)
The Americas, studies (%)	7 (26%)	14 (4%)
Year of enrolment[†]		
Pre-2009, studies (%)	3 (11%)	18 (44%)
2009-2017, studies (%)	24 (89%)	23 (56%)
Follow up duration (days)		
28	5 (19%)	12 (29%)
>28 to 42	5 (19%)	5 (12%)
>42 to <180	2 (7%)	10 (24%)
180	4 (15%)	1 (2%)
>180	11 (41%)	13 (32%)
Age (years), median (IQR)	16 (10-28)	23 (11-25)*[§]
Female, % of patients, median (IQR)	40	37 (30-43)*[¶]

IQR – interquartile range; [#] Multinational studies considered as a proportion of the number of study sites; * Age, and female percentage of targeted studies were calculated using frequency weighted mean or median according to number of patients; † Year of enrolment defined as the year study enrolment completed; § Mean or median age not available from 16 studies; ¶ Percentage not available from 7 studies.

Table S9. Demographics and baseline characteristics for efficacy analysis by primaquine category

	Overall	No PQ	Very low dose total PQ (<2 mg/kg)	Low dose total PQ (2 to <5 mg/kg)	High dose total PQ (≥5 mg/kg)	Intermittent PQ
	N=2,892	N=577	N=28	N=1,043	N=1,207	N=37
Sex						
Male	1,615 (55.8%)	330 (57.2%)	17 (60.7%)	594 (57.0%)	655 (54.3%)	19 (51.4%)
Female	1,277 (44.2%)	247 (42.8%)	11 (39.3%)	449 (43.0%)	552 (45.7%)	18 (48.6%)
Age (years)						
Median (IQR)	9.0 (6.0-12.0)	8.5 (5.5-11.7)	7.7 (5.5-11.5)	8.0 (5.3-11.0)	9.0 (6.0-12.0)	9.0 (6.0-12.1)
5-<15	2,354 (81.4%)	468 (81.1%)	23 (82.1%)	845 (81.0%)	989 (81.9%)	29 (78.4%)
1-<5	533 (18.4%)	107 (18.5%)	5 (17.9%)	198 (19.0%)	216 (17.9%)	7 (18.9%)
<1	5 (0.2%)	2 (0.3%)	0 (0.0%)	0 (0.0%)	2 (0.2%)	1 (2.7%)
Enrolment variables						
Weight (kg)	23.0 (16.0-32.0)	23.0 (16.0-31.0)	22.0 (15.6-32.2)	24.0 (15.0-34.0)	22.9 (16.0-31.1)	28.0 (16.0-36.0)
Malnutrition	123/565 (21.8%)	34/132 (25.8%)	2/7 (28.6%)	32/160 (20.0%)	55/258 (21.3%)	0/8 (0.0%)
Presence of fever	2,515/2,729 (92.2%)	553/577 (95.8%)	25/28 (89.3%)	782/880 (88.9%)	1,121/1,207 (92.9%)	34/37 (91.9%)
Parasitaemia, parasites/mL	3493.8 (1100.0-8845.0)	4560.0 (2000.0-10800.0)	6037.0 (3625.0-8100.0)	2400.0 (750.0-5800.0)	4170.4 (1111.1-11520.0)	5105.5 (2067.3-8413.5)
Haemoglobin, g/dL	11.6 (1.5)	11.5 (1.5)	10.9 (1.6)	11.4 (1.7)	11.7 (1.4)	11.4 (1.7)
Schizontocidal treatment						
Chloroquine	1,794 (62.0%)	349 (60.5%)	7 (25.0%)	788 (75.6%)	619 (51.3%)	31 (83.8%)
Artemether-lumefantrine	109 (3.8%)	55 (9.5%)	0 (0.0%)	45 (4.3%)	9 (0.7%)	0 (0.0%)
Artesunate	65 (2.2%)	65 (11.3%)	0 (0.0%)	0 (0.0%)	0 (0.0%)	0 (0.0%)
Artesunate-amodiaquine	136 (4.7%)	0 (0.0%)	0 (0.0%)	108 (10.4%)	28 (2.3%)	0 (0.0%)
Artesunate-mefloquine	1 (0.0%)	0 (0.0%)	1 (3.6%)	0 (0.0%)	0 (0.0%)	0 (0.0%)
Dihydroartemisinin-piperaquine	755 (26.1%)	91 (15.8%)	5 (17.9%)	102 (9.8%)	551 (45.7%)	6 (16.2%)
Artesunate sulfadoxine-pyrimethamine	32 (1.1%)	17 (2.9%)	15 (53.6%)	0 (0.0%)	0 (0.0%)	0 (0.0%)
Primaquine dosing						
PQ total mg/kg dose	5.5 (3.1-7.2)		0.8 (0.6-1.3)	3.0 (3.0-3.8)	7.1 (6.6-8.1)	5.4 (4.1-6.0)
PQ start day	0.0 (0.0-0.0)		2.0 (0.0-2.0)	0.0 (0.0-0.0)	0.0 (0.0-0.0)	0.0 (0.0-0.0)
Primaquine duration						
7-9 days	1,087/2,268 (47.9%)		4/27 (14.8%)	575/1,043 (55.1%)	508/1,198 (42.4%)	
14 days	1,181/2,268 (52.1%)		23/27 (85.2%)	468/1,043 (44.9%)	690/1,198 (57.6%)	
PQ supervised						
Unsupervised	128/2,315 (5.5%)		18/28 (64.3%)	81/1,043 (7.8%)	29/1,207 (2.4%)	0/37 (0.0%)
Partially supervised	808/2,315 (35.9%)		3/28 (10.7%)	753/1,043 (72.2%)	52/1,207 (4.3%)	0/37 (0.0%)
Fully supervised	1,379/2,315 (59.6%)		7/28 (25.0%)	209/1,043 (20.0%)	1,126/1,207 (93.3%)	37/37 (100.0%)
Relapse periodicity						
Low periodicity	1,126 (38.9%)	317 (54.9%)	22 (78.6%)	366 (35.1%)	393 (32.6%)	28 (75.7%)
High periodicity	1,766 (61.1%)	260 (45.1%)	6 (21.4%)	677 (64.9%)	814 (67.4%)	9 (24.3%)
Transmission intensity						
Low	501 (17.3%)	58 (10.1%)	2 (7.1%)	164 (15.7%)	247 (20.5%)	30 (81.1%)

Moderate	1,377 (47.6%)	212 (36.7%)	21 (75.0%)	563 (54.0%)	578 (47.9%)	3 (8.1%)
High	1,014 (35.1%)	307 (53.2%)	5 (17.9%)	316 (30.3%)	382 (31.6%)	4 (10.8%)
Region						
Africa	426 (14.7%)	136 (23.6%)	19 (67.9%)	78 (7.5%)	193 (16.0%)	0 (0.0%)
Americas	200 (6.9%)	0 (0.0%)	2 (7.1%)	157 (15.1%)	17 (1.4%)	24 (64.9%)
Asia-Pacific	2,266 (78.4%)	441 (76.4%)	7 (25.0%)	808 (77.5%)	997 (82.6%)	13 (35.1%)
Study follow up						
<180 days	681 (23.5%)	27 (4.7%)	17 (60.7%)	626 (60.0%)	11 (0.9%)	0 (0.0%)
≥180 days	2,211 (76.5%)	550 (95.3%)	11 (39.3%)	417 (40.0%)	1,196 (99.1%)	37 (100.0%)
G6PD status (data from qualitative or quantitative test)						
<30% activity	38/2,072 (1.8%)	21/560 (3.8%)	1/15 (6.7%)	5/351 (1.4%)	1/1,133 (0.1%)	10/13 (76.9%)
≥30% activity	2,034/2,072 (98.2%)	539/560 (96.2%)	14/15 (93.3%)	346/351 (98.6%)	1,132/1,133 (99.9%)	3/13 (23.1%)
CYP2D6 metaboliser status						
Poor	1/40 (2.5%)			1/40 (2.5%)		
Intermediate	7/40 (17.5%)			7/40 (17.5%)		
Extensive	30/40 (75.0%)			30/40 (75.0%)		
Ultra	2/40 (5.0%)			2/40 (5.0%)		

CI – confidence interval; G6PD – glucose-6-phosphate dehydrogenase; IQR – interquartile range; PQ – primaquine; sd – standard deviation; Data recorded as number (%), median (IQR), mean (SD) or n/N (%); Primaquine dosing and primaquine supervision are based on 2,315 patients administered primaquine; Data were not available for 461 patients on weight, 2,327 patients on malnutrition, 163 patients on presence of fever, 526 patients on day 0 haemoglobin, 820 patients on G6PD status and 2,852 patients on CYP2D6 status.

Figure S3. Box plot of primaquine dose by age

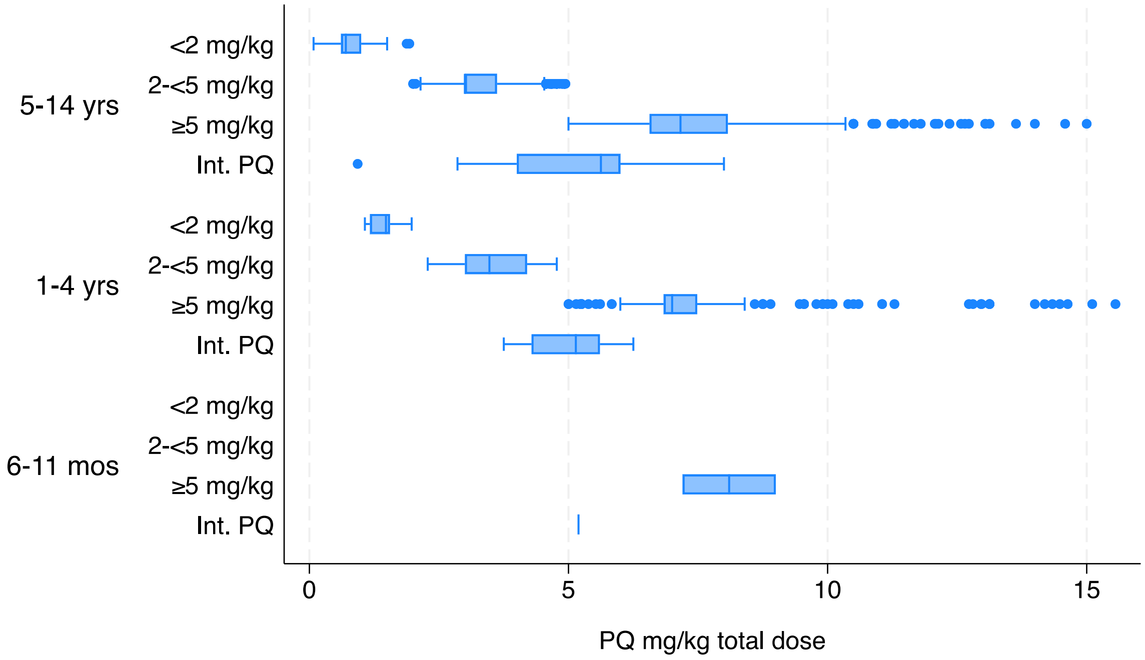

Int. – intermittent; PQ – primaquine. Values above and below 1.5× the interquartile range from quarter 1 to quarter 3 are shown as outliers.

Figure S4. Scatter plot of primaquine dose by A) age and B) weight categorised by presence of recurrence between day 7 and day 180

A:

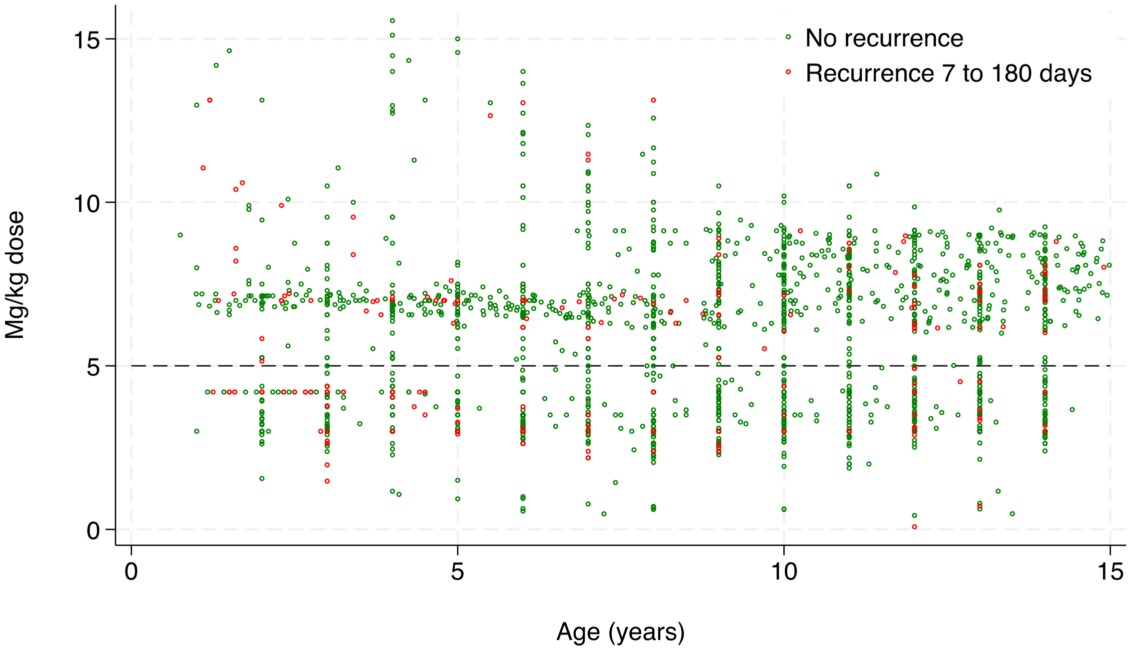

B:

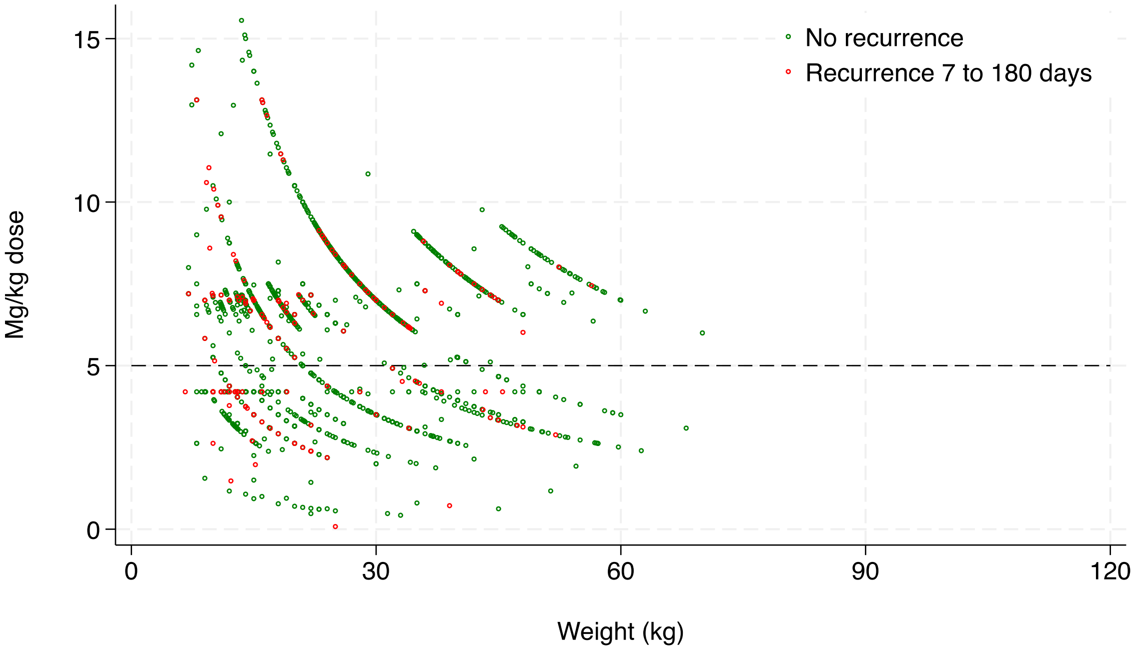

Figure S5. Kaplan-Meier figure of cumulative incidence of recurrence between day 7 and day 365 for A) total primaquine dosing and use and B) intermittent primaquine dosing

A:

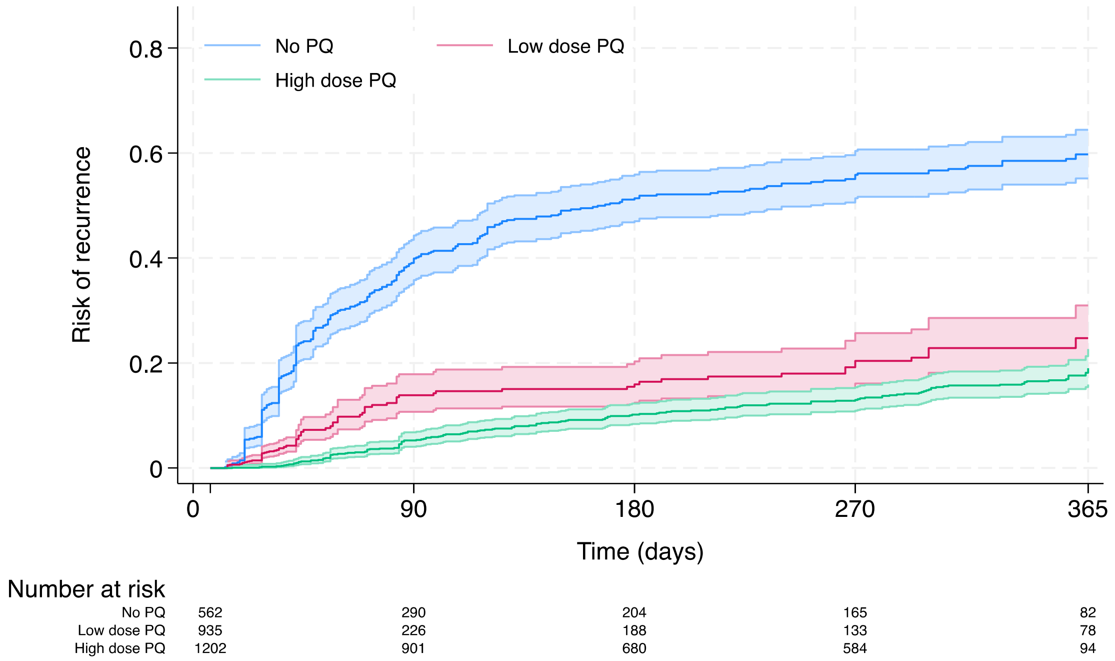

B:

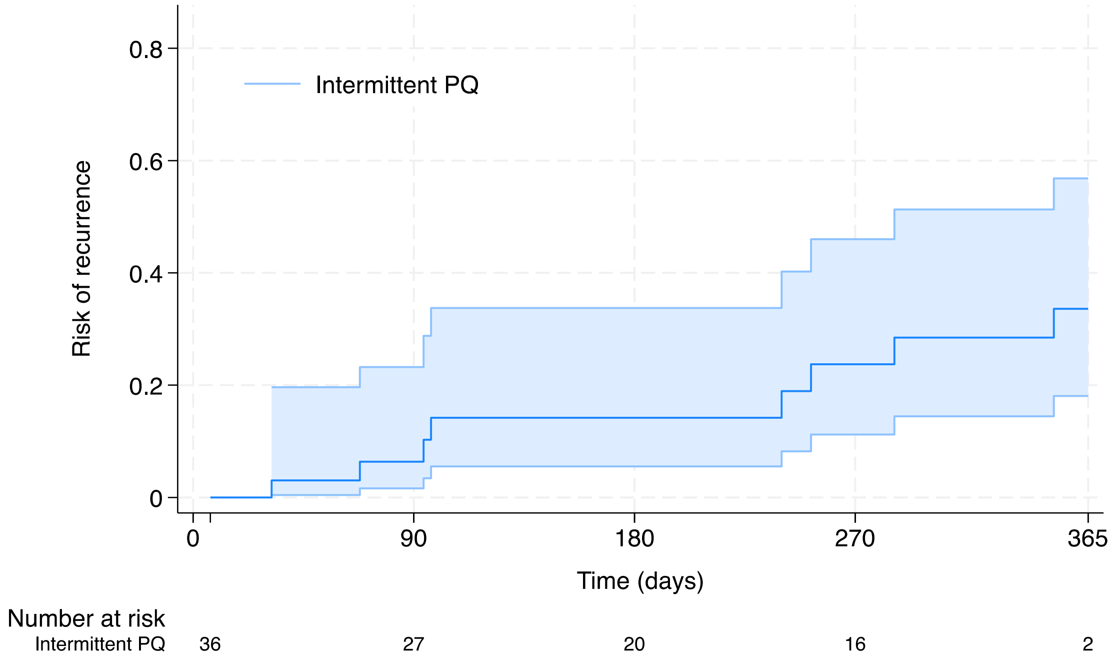

Shading shows 95% confidence interval; PQ – primaquine. By day 180, in the no PQ group there were 262 (46.6%) patients with recurrences and 96 (17.1%) patients censored, in the low dose PQ group there were 81 (8.7%) patients with recurrences and 666 (71.2%) patients censored and in the high dose PQ group there were 98 (8.2%) patients with recurrences and 424 (35.3%) patients censored. Log rank test no PQ vs low dose PQ $p<0.0001$, log rank test no PQ vs high dose PQ $p<0.0001$, log rank test low dose PQ vs high dose PQ $p<0.0001$.

Table S10. Cumulative incidence of recurrence between day 7 and days 42, 90, 180 and 365 by study and primaquine category

Study	Kaplan-Meier cumulative incidence (%, 95% CI)			
	Day 42	Day 90	Day 180	Day 365
Hasugian-2007[15]				
No primaquine	-	-	-	-
Low dose primaquine	34.7 (24.1-48.2)	-	-	-
High dose primaquine	-	-	-	-
Barber-2013[29]				
No primaquine	0	-	-	-
Low dose primaquine	-			
High dose primaquine	-	-	-	-
Pasaribu-2013[17]				
No primaquine	-	-	-	-
Low dose primaquine	0	-	-	-
High dose primaquine	0	0	-	-
Gonzalez-Ceron-2015[18]				
No primaquine	-	-	-	-
Low dose primaquine	0	0	0	0
High dose primaquine	0	0	0	0
Yuan-2015[33]				
No primaquine	-	-	-	-
Low dose primaquine	6.0 (3.8-9.4)	-	-	-
High dose primaquine	-	-	-	-
Longley-2016[35]				
No primaquine	-	-	-	-
Low dose primaquine	0	0	0	
High dose primaquine	-	-	-	-
Zuluaga-Idarraga-2016[36]				
No primaquine	-	-	-	-
Low dose primaquine	0	6.7 (1.0-38.7)	20.6 (7.1-51.2)	-
High dose primaquine	0	0	0	-
Abreha-2017[19]				
No primaquine	37.5 (27.5-49.7)	58.3 (47.1-70.0)	64.3 (53.1-75.4)	-
Low dose primaquine	5.5 (2.1-14.0)	15.3 (8.8-25.9)	19.7 (12.2-31.0)	-
High dose primaquine	0	0	0	-
Awab-2017[20]				
No primaquine	2.3 (0.7-6.8)	6.3 (3.2-12.2)	13.7 (8.7-21.1)	25.8 (18.6-35.1)
Low dose primaquine	0	0	0	9.5 (4.9-18.2)
High dose primaquine	-	-	-	-
Chu-2018[21]				
No primaquine	53.2 (44.9-61.9)	68.6 (60.5-76.5)	76.3 (68.3-83.6)	78.2 (69.8-85.5)
Low dose primaquine	0	0	0	0
High dose primaquine	2.7 (0.7-10.4)	4.1 (1.3-12.1)	5.6 (2.1-14.3)	24.8 (15.6-37.9)
Grigg-2018[38]				
No primaquine	55.6 (21.5-93.4)	-	-	-
Low dose primaquine	0	-	-	-
High dose primaquine	16.7 (2.5-72.7)	-	-	-
Hamid-2018[22]				
No primaquine	6.7 (1.0-38.7)	-	-	-
Low dose primaquine	-	-	-	-
High dose primaquine	-	-	-	-
Chu-2019[23]				
No primaquine	-	-	-	-
Low dose primaquine	0	33.3 (5.5-94.6)	33.3 (5.5-94.6)	33.3 (5.5-94.6)
High dose primaquine	0.5 (0.1-3.7)	6.4 (3.7-11.0)	10.7 (7.0-16.3)	15.9 (11.1-22.7)

De Sena-2019[39]				
No primaquine	-	-	-	-
Low dose primaquine	0	-	-	-
High dose primaquine	0	-	-	-
Ladeia-Andrade-2019[24]				
No primaquine	-	-	-	-
Low dose primaquine	28.6 (8.0-74.2)	0	-	-
High dose primaquine	0	-	-	-
Rijal-2019[25]				
No primaquine	0	-	-	-
Low dose primaquine	0	-	-	-
High dose primaquine	-	-	-	-
Taylor-2019[26]				
No primaquine	9.6 (6.1-15.0)	35.2 (28.6-42.9)	55.7 (48.1-63.6)	64.7 (56.9-72.5)
Low dose primaquine	0	0	0	0
High dose primaquine	0.8 (0.4-1.8)	3.0 (2.0-4.6)	8.0 (6.1-10.4)	19.1 (12.4-28.8)
Poespoprodjo-2022[27]				
No primaquine				
Low dose primaquine	0	16.7 (4.5-51.8)	16.7 (4.5-51.8)#	
High dose primaquine	1.7 (0.2-11.4)	28.2 (17.7-43.0)	39.5 (27.2-54.9)#	
Karunajeewa-unpublished				
No primaquine	42.9 (16.3-82.8)	71.4 (38.9-95.9)*	-	-
Low dose primaquine	28.6 (8.0-74.2)	42.9 (16.3-82.8)*	-	-
High dose primaquine	0	50.0 (19.6-88.9)*	-	-

CI – confidence interval; Cumulative risk was unable to be determined for some treatment groups due to inclusion of small numbers of children; * Cumulative incidence at day 84; # cumulative incidence at day 173

Table S11. Sensitivity analyses estimating hazard ratios for *Plasmodium vivax* recurrence between days 7 and 180 by total primaquine dose

	Adjusted hazard ratio (95% CI)					
**Total primaquine dose**	**Recurrence between day 28 and 180**	**Patients where actual PQ dose recorded**	**Patients treated with chloroquine**	**Studies with ≥180 days follow up**	**Studies which compared PQ versus no PQ**	**Analysis without shared frailty**
	**n=2,699**	**n=1,888**	**n=1,627**	**n=2,137**	**n=1,625**	**n=2,699**
None	Reference	Reference	Reference	Reference	Reference	Reference
Low	0.18 (0.11, 0.27)	0.16 (0.08, 0.31)	0.16 (0.09, 0.30)	0.14 (0.09, 0.22)	0.18 (0.11, 0.31)	0.27 (0.21, 0.35)
High	0.10 (0.07, 0.13)	0.09 (0.07, 0.12)	0.08 (0.05, 0.11)	0.09 (0.07, 0.12)	0.09 (0.06, 0.12)	0.14 (0.11, 0.17)

CI – confidence interval; PQ – primaquine; Sensitivity analyses for multivariable Cox regression model adjusted for primaquine treatment category, age, sex and baseline parasitaemia with shared frailty for study site unless otherwise stated.

Table S12. Sensitivity analysis excluding one study at a time for hazard ratio of *Plasmodium vivax* recurrence between days 7 and 180 following primaquine treatment group compared with no primaquine

Total primaquine dose	Range of AHR	Coefficient of Variation (%)
None	Reference	-
Low	0.15-0.22	6.25
High	0.08-0.10	3.28

AHR – adjusted hazard ratio; Sensitivity analyses for multivariable Cox regression model adjusted for primaquine treatment category, age sex and baseline parasitaemia with shared frailty for study site.

Text S2. Additional efficacy analysis results

Low dose primaquine versus high dose primaquine: The rate of recurrence was significantly lower following high dose primaquine in children (AHR 0.54, 0.35-0.85). These results were consistent in multiple sensitivity analyses (Tables S11-S12). In a subgroup analysis, there was a similar reduction in the rate of recurrence following high dose compared with low dose primaquine in children in both low relapse periodicity regions (AHR 0.52, 0.27-1.00) and high relapse periodicity regions (AHR 0.64, 0.33 to 1.25).

Effect of the total dose of primaquine as a continuous variable: In a Cox model controlling for age, sex and baseline parasitaemia with total mg/kg primaquine dose as a continuous variable, there was no change in the rate of recurrence between day 7 and 180 for children following a 1 mg/kg increase in total primaquine dose (AHR 0.94, 95%CI 0.84 to 1.05). However, analysis between studies showed marked heterogeneity in the rate of recurrence with significantly high risks of recurrences with primaquine in studies conducted in Melanesia; Table S10.[15,27] This region is recognised as having the highest reported relapse periodicity globally.[49] In a post-hoc analysis excluding the Melanesian studies there was a significant reduction in the rate of recurrence between day 7 and 180 following a 1 mg/kg increase in total primaquine dose, that was apparent in children (AHR 0.84, 95%CI 0.72 to 0.99); Supplementary Figure S6).

Effect of duration of primaquine: Data on the duration of primaquine treatment were available for 2,241 children. Of 1,043 children treated with low total dose primaquine, 575 (55.1%) were treated for 7 days and 468 (44.9%) for 14 days. Of 1,198 children treated with high dose primaquine, 508 (42.4%) were treated for 7 days and 690 (57.6%) for 14 days. After adjusting for total primaquine mg/kg dose, age, sex and baseline parasitaemia, there was a non-significant trend for a lower rate of recurrence between day 7 and 180, following a 14-day low dose primaquine regimen compared with a low dose 7-day regimen (AHR 0.76, 95%CI 0.14-4.10). The same trend was apparent for children treated with a 14-day high dose primaquine regimen compared to a 7-day high dose regimen (AHR 0.75 (0.48-1.18); Figure 2).

Figure S6. Adjusted hazard ratio for recurrence between day 7 and 180 compared with primaquine total mg/kg dose for children

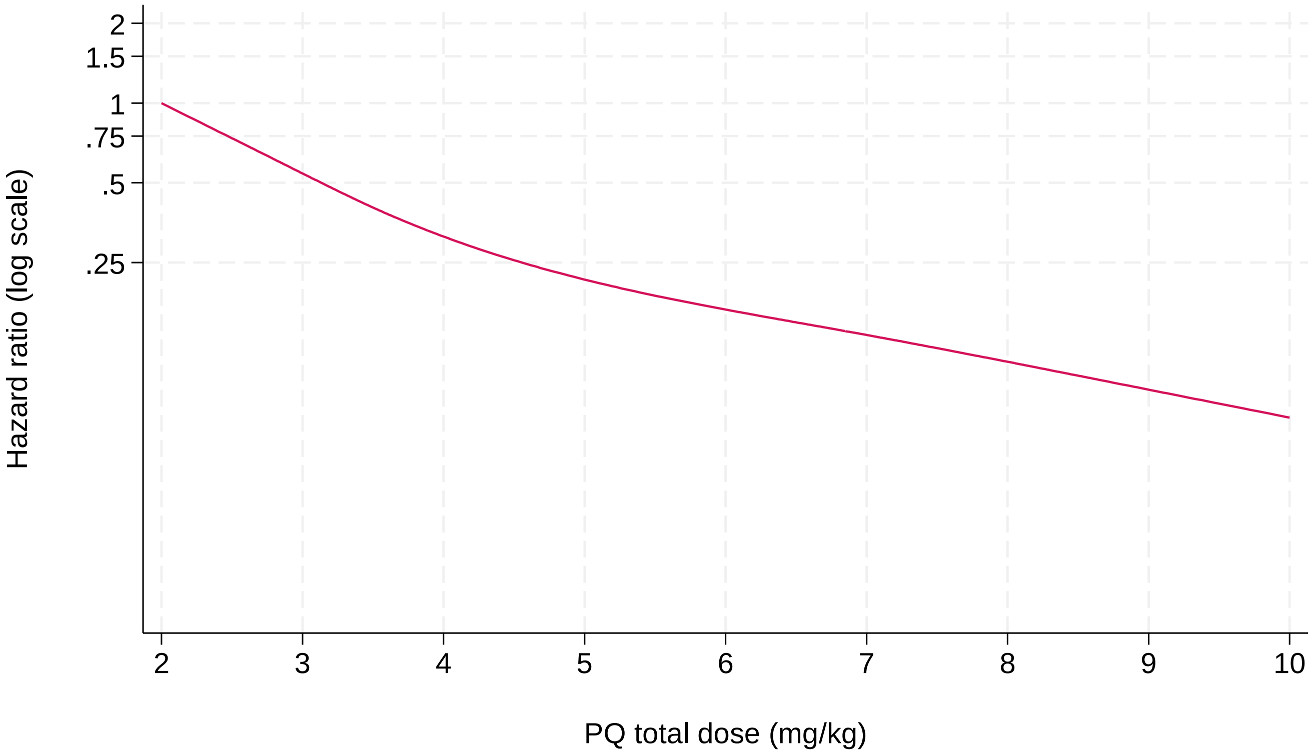

PQ – primaquine; Spline model with 4 knots at default locations (5th, 35th, 65th and 95th percentiles) based on Cox regression model adjusted for age, sex and baseline parasitaemia with shared frailty for study site. Three studies from Melanesia (including one unpublished study) have been excluded.[15,27]

Table S13. Baseline characteristics and demographic features for gastrointestinal tolerability analysis by age categories

	Total<br>N=1,993	Children ≥5 to <15 years<br>N=1,615	Children <5 years<br>N=378
**Daily PQ dose (mg/kg) category**			
No primaquine	430 (21.6%)	361 (22.4%)	69 (18.3%)
Low dose daily primaquine (<0.375 mg/kg/day)	450 (22.6%)	346 (21.4%)	104 (27.5%)
Intermediate dose daily primaquine (≥0.375 & <0.75 mg/kg/day)	518 (26.0%)	426 (26.4%)	92 (24.3%)
High dose daily primaquine (≥0.75 mg/kg/day)	595 (29.9%)	482 (29.8%)	113 (29.9%)
**Age (years)**	9.0 (5.9-12.0)	10.0 (7.4-12.0)	3.0 (2.5-4.0)
**Female**	897 (45.0%)	724 (44.8%)	173 (45.8%)
**Weight (kg)**	24.6 (10.9)	27.5 (10.2)	12.5 (2.5)
**Malnutrition**	113 (24.7%)	19 (23.8%)	94 (24.9%)
**Haemoglobin day 0 (g/dL)**	11.6 (1.5)	11.8 (1.5)	10.8 (1.6)
**Parasite density on day 0 (/µL)**	3703.7 (1166.7-9200.0)	3648.1 (1144.4-8940.7)	3990.7 (1296.3-10687.5)
**Fever on day 0**	1,838 (92.9%)	1,477 (92.3%)	361 (95.8%)
**Schizontocidal treatment**			
Artemether-lumefantrine	117 (5.9%)	88 (5.4%)	29 (7.7%)
Artesunate	136 (6.8%)	92 (5.7%)	44 (11.6%)
Artesunate-amodiaquine	32 (1.6%)	30 (1.9%)	2 (0.5%)
Artesunate sulfadoxine-pyrimethamine	1,089 (54.6%)	907 (56.2%)	182 (48.1%)
Chloroquine	619 (31.1%)	498 (30.8%)	121 (32.0%)
**G6PD status (data from qualitative or quantitative test)**			
Deficient (<30% activity)	6 (0.3%)	3 (0.2%)	3 (0.8%)
Normal (≥30% activity)	1,572 (78.9%)	1,275 (78.9%)	297 (78.6%)
Unknown	415 (20.8%)	337 (20.9%)	78 (20.6%)
**G6PD status (data from qualitative or quantitative test if deficient and from quantitative test if ≥30% activity)**			
Deficient (<30%)	6 (0.3%)	3 (0.2%)	3 (0.8%)
Intermediate (≥30-<70%)	18 (0.9%)	15 (0.9%)	3 (0.8%)
Normal (≥70%)	495 (24.8%)	415 (25.7%)	80 (21.2%)
Unknown	1,474 (74.0%)	1,182 (73.2%)	292 (77.2%)
**CYP2D6A metabolizer status**			
Poor	1 (5%)	1 (5%)	
Intermediate	1 (5%)	1 (5%)	
Extensive	20 (91%)	20 (91%)	
**Geographical region**			
Africa	425 (21.3%)	342 (21.2%)	83 (22.0%)
Americas	63 (3.2%)	58 (3.6%)	5 (1.3%)
Asia-Pacific	1,505 (75.5%)	1,215 (75.2%)	290 (76.7%)
**Relapse periodicity**			
Low periodicity	996 (50.0%)	837 (51.8%)	159 (42.1%)
High periodicity	997 (50.0%)	778 (48.2%)	219 (57.9%)
**Transmission intensity of study site**			
Low	595 (29.9%)	502 (31.1%)	93 (24.6%)
Moderate	389 (19.5%)	313 (19.4%)	76 (20.1%)
High	1,009 (50.6%)	800 (49.5%)	209 (55.3%)

Data show number (%), median (IQR) or mean (SD). Data were not available for 7 patients for weight, 1,536 patients for nutrition status, 15 patients for presence of fever on day 0, 55 patients on haemoglobin on day 0 and 1,971 patients for CYP2D6A status. IQR – interquartile range, SD – standard deviation.

Figure S7. Distribution of primaquine daily dose by primaquine mg/kg daily dose category and age category

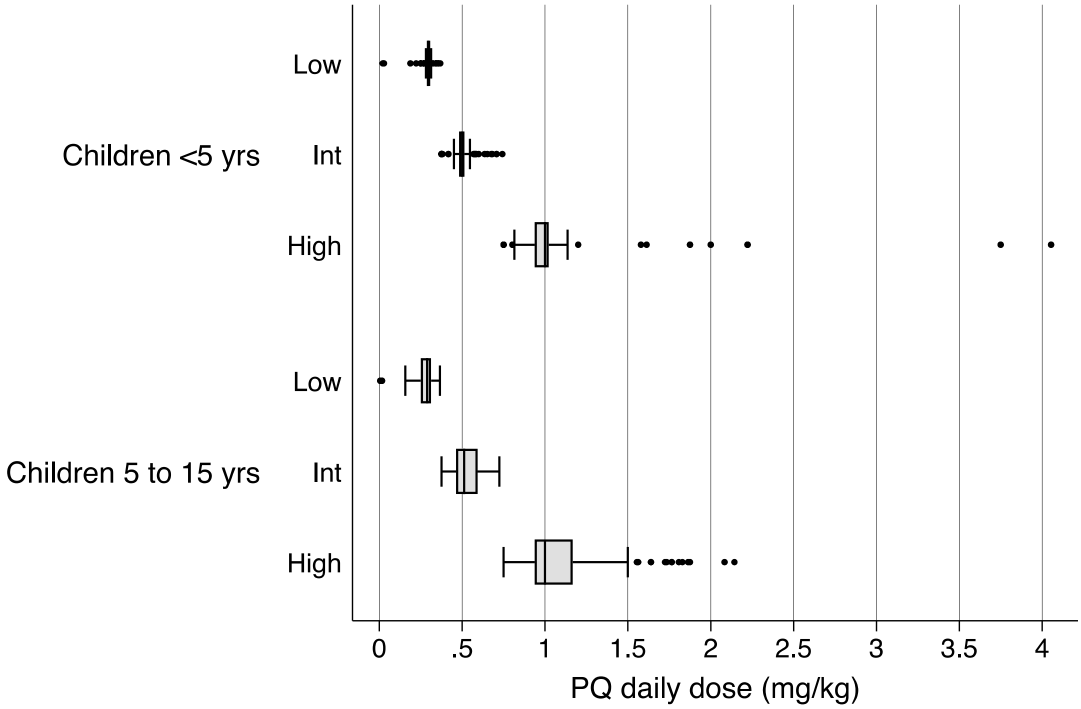

Primaquine daily dose categories: Low = <0.375 mg/kg/day, Int (intermediate) = ≥0.375 mg/kg/day and <0.750 mg/kg/day, and High = ≥0.750 mg/kg/day. Values above and below 1.5× the interquartile range from quarter 1 to quarter 3 are shown as outliers.

Figure S8. Unadjusted risk of gastrointestinal symptoms in children on day 0, days 1-2 and days 5-7

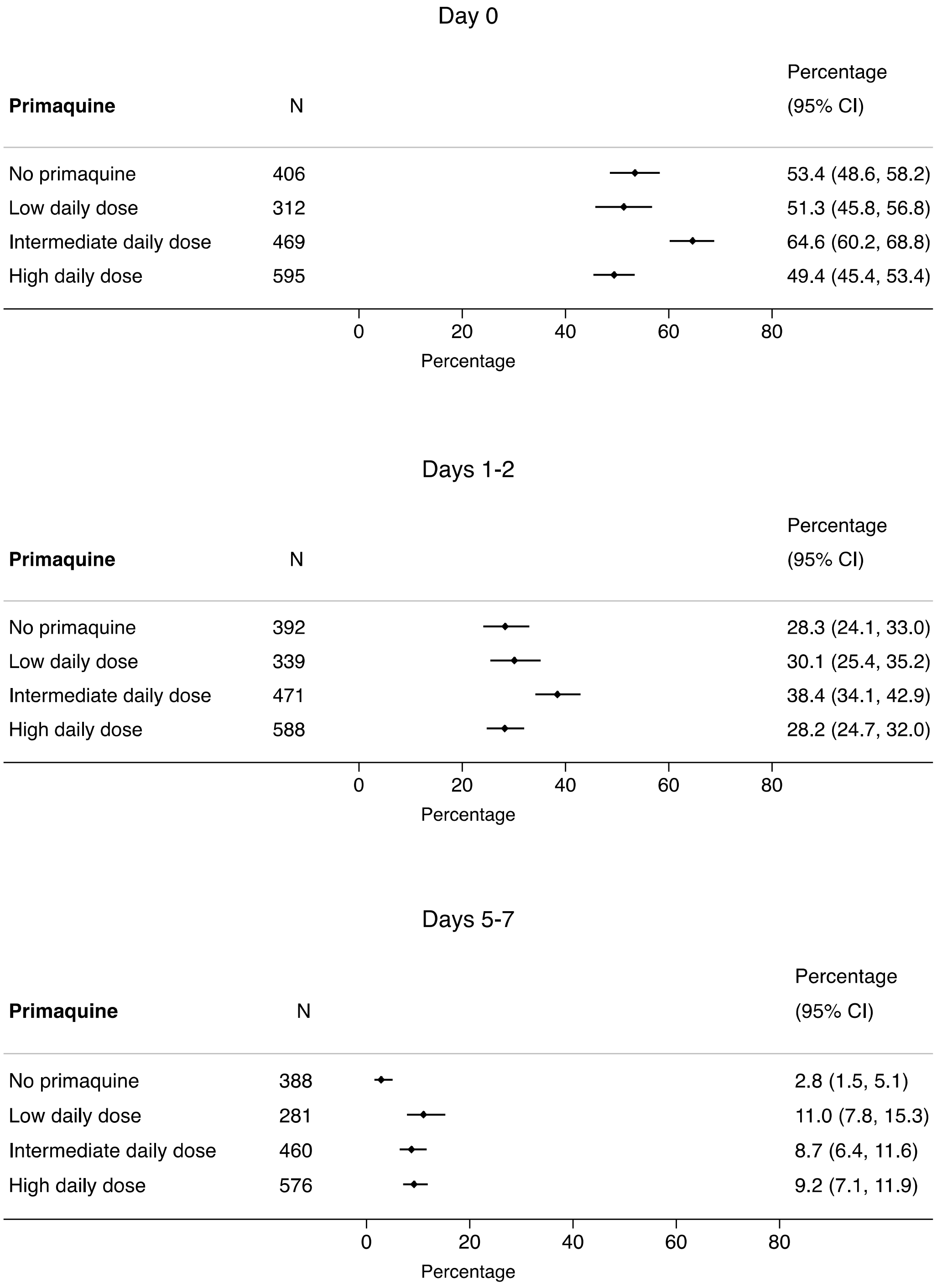

The risk of the composite outcome (composite of vomiting or anorexia or diarrhoea) was calculated for children by primaquine daily dose category as the number of individuals experiencing a symptom as a proportion of the number of individuals asked about the symptom. The Confidence Intervals (CIs) are Agresti-Coull CIs.

Figure S9. Covariate-adjusted estimates for gastrointestinal intolerance on days 5-7 by primaquine daily dose categories and age categories

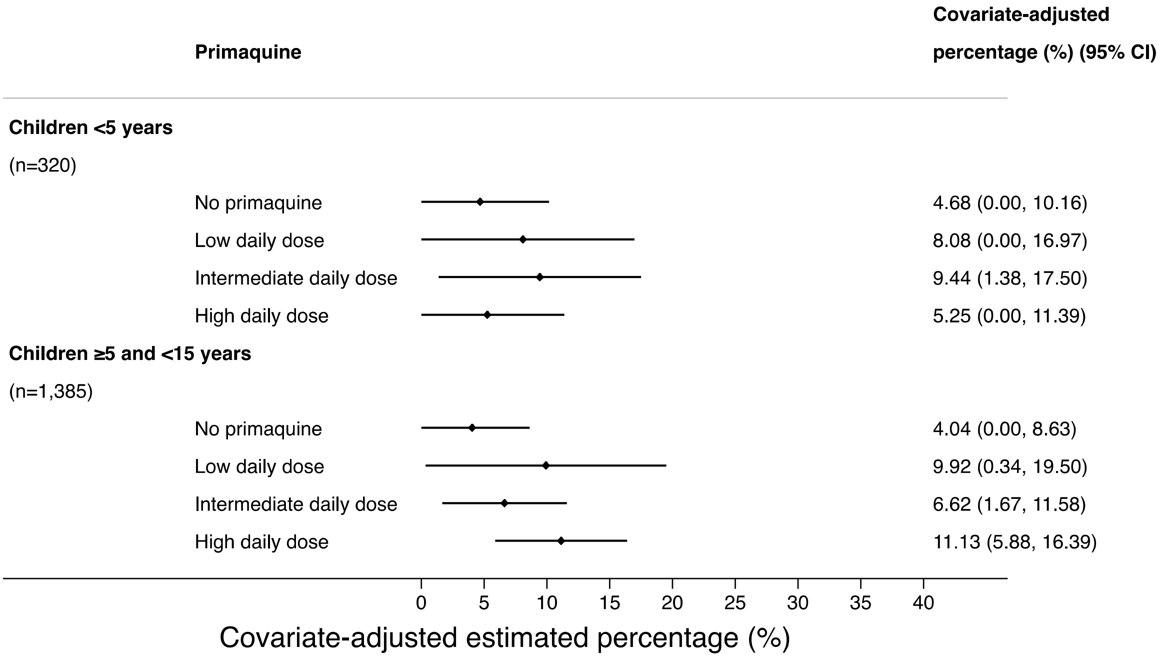

CI – confidence interval; low daily dose ~0.25 mg/kg/day; intermediate daily dose ~5 mg/kg/day; high daily dose - ~1 mg/kg/day; Generalised estimating equations modified Poisson regression models adjusting for age category, sex (male or female) and (1og$_{10}$) baseline parasite density, clustering by study site and cluster robust error estimates. Covariate-adjusted proportions were estimated at the mean values of the covariates.

Table S14. Covariate-adjusted estimated percentages (95% CI) from sensitivity analyses for the composite gastrointestinal tolerability outcome assessed on days 5-7

	Restricted to those asked about all three symptoms that make up the composite outcome<br><br>N=1,224	Adjusting for reporting of symptoms on day 0<br><br>N=1,671	Restricted to studies with a no PQ group as well as a PQ treatment group<br><br>N=1,366
**Children**			
No primaquine	2.5 (0 to 7.5)	2.6 (1.1 to 6.3)	2.4 (1.9 to 6.7)
Low daily dose	15.6 (0 to 34.4)	5.7 (0 to 12.5)	3.4 (0 to 8.1)
Intermediate daily dose	5.4 (0.5 to 10.3)	5.0 (1.0 to 9.1)	5.3 (0.3 to 10.3)
High daily dose	7.9 (3.7 to 12.1)	7.5 (4.0 to 11.0)	8.0 (4.4 to 11.7)

For each analysis, generalised estimating equations modified Poisson models were fitted adjusting for age, sex and (log_{10}) baseline parasite density with a log link, clustering by study site, exchangeable correlation structure and cluster robust error estimates. The covariate-adjusted percentage of patients reporting the outcome was estimated at the mean values of the covariates.

Table S15. Covariate adjusted estimated percentages (95% CI) from sensitivity analyses for the composite gastrointestinal tolerability outcome assessed on days 1-2, restricted to patients who started primaquine on day 0

	Restricted to patients treated with chloroquine<br>N=1,020	Restricted to patients treated with dihydroartemesinin-piperaquine<br>N=471
**Children**		
No primaquine	21.8 (7.0 to 36.6)	44.7 (35.2 to 54.2)
Low daily dose	17.4 (3 to 31.8)	9.5 (8.5 to 10.5)
Intermediate daily dose	26.2 (13.0 to 39.5)	37.4 (29.4 to 45.4)
High daily dose	23.0 (11.0 to 35.1)	42.3 (40.9 to 43.7)

For each analysis, generalised estimating equations modified Poisson models were fitted adjusting for age, sex and (log_{10}) baseline parasite density with a log link, clustering by study site, exchangeable correlation structure and cluster robust error estimates. The covariate-adjusted percentage of patients reporting the outcome was estimated at the mean values of the covariates.

Table S16 Contribution of patients from each study included in the analysis of the primary composite gastrointestinal tolerability end point on days 5-7 to each primaquine dose category

Study	Daily PQ dose (mg/kg) category (n)				Total
	No primaquine	Low dose daily primaquine (<0.375 mg/kg/day)	Intermediate dose daily primaquine (≥0.375 & <0.75 mg/kg/day)	High dose daily primaquine (≥0.75 mg/kg/day)	
Pasaribu-2013	0	22	12	0	34
Thanh-2015	0	0	0	150	150
Hasugian-2007	0	51	0	0	51
Gonzalez-Ceron-2015	0	14	4	0	18
Zuluaga-Idarraga-2016	0	11	6	0	17
Abreha-2017	74	75	2	0	151
Taylor-2019	182	0	362	416	960
Ladeia-Andrade-2019	0	0	20	3	23
Awab-2017	132	100	20	3	255
Poespoprodjo-2021	0	8	34	4	46
Total	388	281	460	576	1,705

Figure S10. Sensitivity analysis for the covariate-adjusted estimated percentage of patients reporting gastrointestinal symptoms (95% CI) assessed on days 5-7, excluding one study at a time

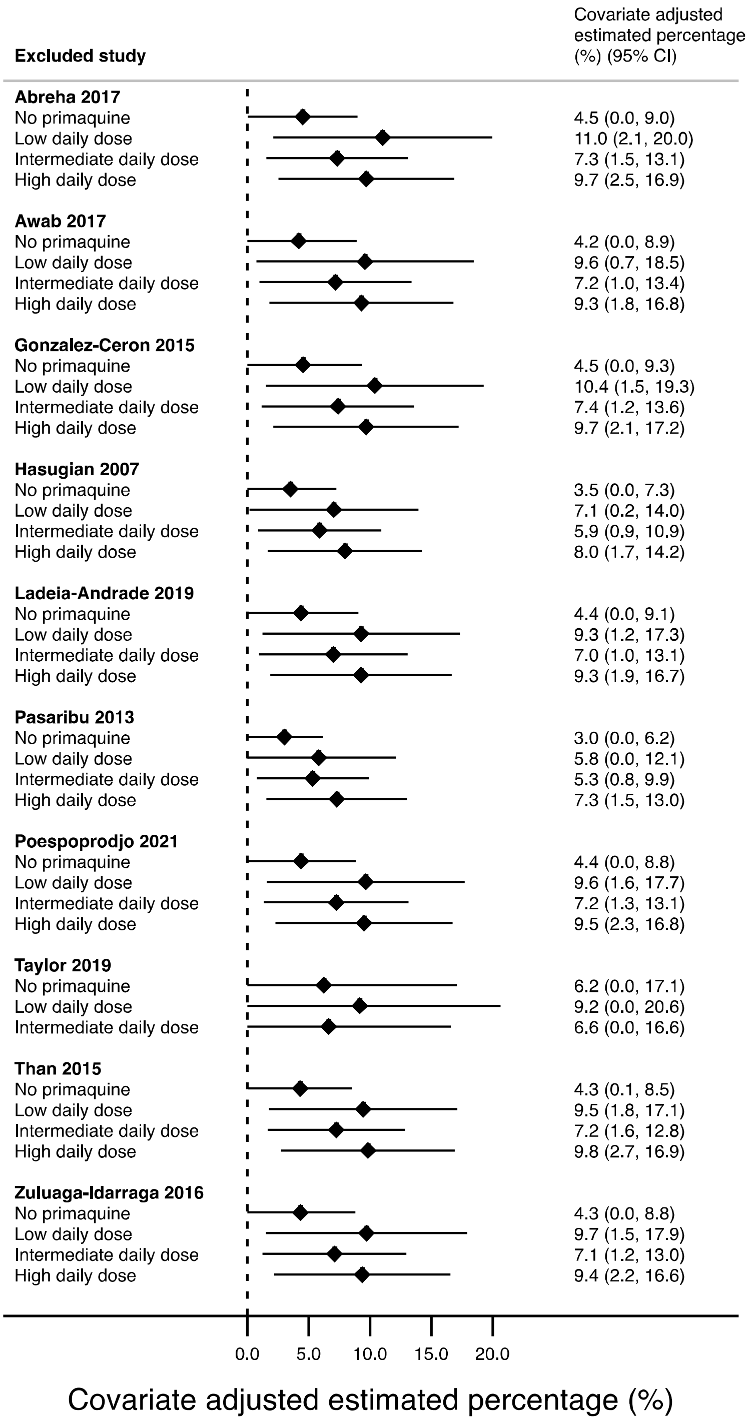

CI – confidence interval; Multivariable logistic regression mixed effects models were fitted adjusting for age, sex and (log_{10}) baseline parasite density with random intercepts by study site. Taylor-2019[26] was influential and affected the estimated proportions. However, after excluding this study children still had a higher percentage of gastrointestinal intolerance following primaquine relative to patients not treated with primaquine, when estimable.

Table S17. Risk of acute vomiting on days 0-2 and 3-13 by primaquine daily dose categories in children

	Risk of acute vomiting Days 0-2		Risk of acute vomiting Days 3-13	
**PQ daily dose category**	**Children <5 yrs**	**Children 5 - <15 yrs**	**Children <5 yrs**	**Children 5 - <15 yrs**
Low	0% (0/28)	0.6% (1/161)	0% (0/16)	0% (0/69)
Intermediate	7.1% (6/85)	1.2% (4/346)	0% (0/62)	0.3% (1/307)
High	1.8% (2/113)	2.1% (10/479)	0% (0/104)	0.2% (1/469)

Table S18. Baseline characteristics and demographic features of patients included in the haematological safety analysis by age groups

	Total N=2,539	Children ≥5 to <15 years N=2,084	Children <5 years N=455
**Daily PQ dose (mg/kg) category**			
No primaquine	626 (24.7%)	516 (24.8%)	110 (24.2%)
Low dose daily primaquine (<0.375 mg/kg/day)	413 (16.3%)	324 (15.5%)	89 (19.6%)
Intermediate dose daily primaquine (≥0.375 & <0.75 mg/kg/day)	735 (28.9%)	612 (29.4%)	123 (27.0%)
High dose daily primaquine (≥0.75 mg/kg/day)	698 (27.5%)	574 (27.5%)	124 (27.3%)
Intermittent PQ	67 (2.6%)	58 (2.8%)	9 (2.0%)
**Age (years)**	**9.0 (6.0-12.0)**	**10.0 (7.1-12.0)**	**3.2 (2.5-4.0)**
**Female**	1,160 (45.7%)	947 (45.4%)	213 (46.8%)
**Weight (kg)**	**24.5 (10.8)**	**27.1 (10.1)**	**12.4 (2.5)**
**Malnutrition**	149 (25.9%)	25 (20.7%)	124 (27.3%)
**Haemoglobin day 0 (g/dL)**	**11.6 (1.5)**	**11.8 (1.4)**	**10.7 (1.5)**
**Parasite density on day 0 (/µL)**	3800.0 (1196.0-9720.0)	3759.3 (1183.8-9563.0)	4040.0 (1260.0-10780.0)
**Fever on day 0**	**2,205 (93.8%)**	**1,780 (93.4%)**	**425 (95.7%)**
**Schizontocidal treatment**			
Artemether-lumefantrine	119 (4.7%)	85 (4.1%)	34 (7.5%)
Artesunate	62 (2.4%)	43 (2.1%)	19 (4.2%)
Artesunate-amodiaquine	124 (4.9%)	88 (4.2%)	36 (7.9%)
Artesunate sulfadoxine-pyrimethamine	32 (1.3%)	30 (1.4%)	2 (0.4%)
Chloroquine	1,464 (57.7%)	1,233 (59.2%)	231 (50.8%)
Dihydroartemisinin-piperaquine	738 (29.1%)	605 (29.0%)	133 (29.2%)
**G6PD status (data from qualitative or quantitative test)**			
Deficient (<30% activity)	38 (1.5%)	28 (1.3%)	10 (2.2%)
Normal (≥30% activity)	2,144 (84.4%)	1,768 (84.8%)	376 (82.6%)
Unknown	357 (14.1%)	288 (13.8%)	69 (15.2%)
**G6PD status (data from qualitative or quantitative test if deficient and from quantitative test if ≥30% activity)**			
Deficient (<30%)	38 (1.5%)	28 (1.3%)	10 (2.2%)
Intermediate (≥30-<70%)	19 (0.7%)	16 (0.8%)	3 (0.7%)
Normal (≥70%)	507 (20.0%)	426 (20.4%)	81 (17.8%)
Unknown	1,975 (77.8%)	1,614 (77.4%)	361 (79.3%)
**CYP2D6A metabolizer status**			
Poor	1 (5%)	1 (5%)	-
Intermediate	1 (5%)	1 (5%)	-
Extensive	20 (91%)	20 (91%)	-
**Geographical region**			
Africa	419 (16.5%)	335 (16.1%)	84 (18.5%)
Americas	27 (1.1%)	27 (1.3%)	0 (0.0%)
Asia-Pacific	2,093 (82.4%)	1,722 (82.6%)	371 (81.5%)
**Relapse periodicity**			
Low periodicity	1,092 (43.0%)	928 (44.5%)	164 (36.0%)
High periodicity	1,447 (57.0%)	1,156 (55.5%)	291 (64.0%)
**Transmission intensity of study site**			
Low	583 (23.0%)	493 (23.7%)	90 (19.8%)
Moderate	964 (38.0%)	802 (38.5%)	162 (35.6%)
High	992 (39.1%)	789 (37.9%)	203 (44.6%)

Data show number (%), median (IQR) or mean (SD). Data were not available for 9 patients for weight, 1,964 patients for nutrition status, 191 patients for parasite density on day 0, 189 patients for presence of fever on day 0 and 2,517 patients for CYP2D6A status. IQR – interquartile range, SD – standard deviation.

Table S19. Details of patients experiencing haematological outcomes

ID	Sex	Age	PQ daily dose category	G6PD activity	Hb on day 0	Lowest Hb on days 1-13	Day of lowest Hb	Day 2-3 Hb	Day 5-7 Hb	Hb drop ≥25% to <7 g/dL	Hb drop >5 g/dL	Blood transfusion required
1	Female	13	High	61.5	8.8	4.4	5	5.5	4.4	Yes	No	Yes
2	Male	5	Intermediate	≥30%	10.1	5.9	3	5.9	7.84	Yes	No	No
3	Female	5	No PQ	≥30%	10.3	6.3	3	6.3	9.9	Yes	No	.
4	Female	12	High	≥30%	10.5	6.3	7	9.0	6.3	Yes	No	No
5	Female	4	No PQ	≥30%	9.6	6.4	3	6.4	10	Yes	No	.
6	Female	10	Intermediate	≥30%	9.0	6.7	5	7.1	6.7	Yes	No	No
7	Female	13	High	≥30%	11.0	6.7	6	7.81	6.7	Yes	No	No
8	Male	3	Intermediate	≥30%	10.2	6.8	3	6.8	7.7	Yes	No	No
9	Female	10	High	37.4%	11.6	6.9	3	6.9	8.4	Yes	No	No
10	Female	10	Intermediate	75.9%	12.6	7.5	5	10.11	7.5	No	Yes	No
11	Female	12	High	130.5%	13.9	8.8	7	11.4	8.8	No	Yes	No
12	Male	14	High	≥30%	14.4	9.4	8	14.4	14.4	No	Yes	No

Hb – haemoglobin; PQ – primaquine

Figure S11. Estimated percentage of children with G6PD activity ≥30% developing mild, moderate or severe anaemia on days 2-3 or days 5-7 by primaquine daily dose groups

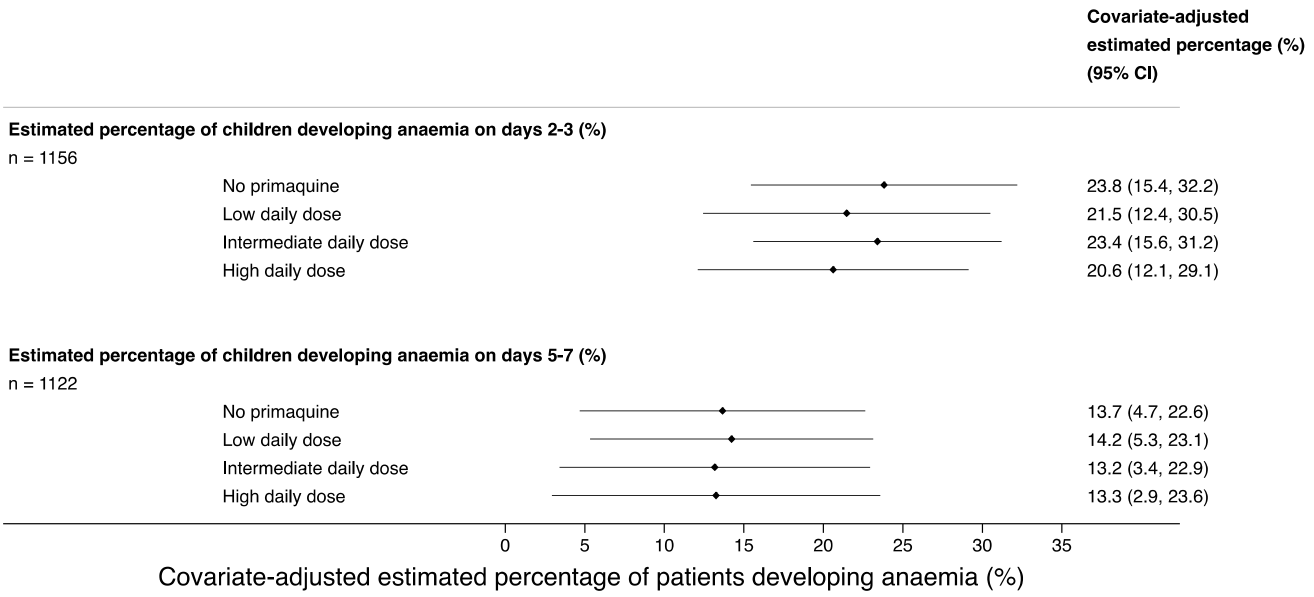

CI – confidence interval; low daily dose ~0.25 mg/kg/day; intermediate daily dose ~5 mg/kg/day; high daily dose - ~1 mg/kg/day; Generalised estimating equations modified Poisson regression models adjusting for age, day 0 haemoglobin, sex (male or female) and ($1og_{10}$) baseline parasite density, clustering by study site and cluster robust error estimates. Covariate-adjusted percentages were estimated at the mean values of the covariates.

Table S20. Baseline characteristics and demographic features of patients in the adverse event meta-analysis, by age category

		Age group (years)	
	**Overall N=1,797**	**<5 N=293**	**5-<15 N=1,504**
Sex			
Male	998 (55.5)	154 (52.6)	844 (56.1)
Female	799 (44.5)	139 (47.4)	660 (43.9)
Enrolment variables			
Age, years	9.0 (6.0-12.0)	3.2 (2.4-4.0)	10.0 (7.5-12.4)
Weight (kg)	23.4 (16.4-32.0)	12.0 (11.0-14.1)	26.0 (20.0-34.0)
Presence of fever	1,686 (93.8)	280 (95.6)	1,406 (93.5)
Parasitaemia, parasites/mL	4,400 (1,596-10,900)	5,281(2,037-12,009)	4,176 (1,504-10,676)
Haemoglobin day 0, g/dL	11.7 (1.4)	10.9 (1.3)	11.8 (1.4)
Schizontocidal treatment			
Chloroquine	1,052 (58.5)	171 (58.4)	881 (58.6)
Artemether-lumefantrine	82 (4.6)	18 (6.1)	64 (4.3)
Artesunate	55 (3.1)	18 (6.1)	37 (2.5)
Dihydroartemisinin-piperaquine	576 (32.1)	84 (28.7)	492 (32.7)
Artesunate-SP	32 (1.8)	2 (0.7)	30 (2.0)
Primaquine dosing			
PQ total mg/kg dose	7.0 (6.4-7.8)	7.0 (6.6-7.1)	7.0 (6.3-8.0)
Primaquine total dose categories			
No primaquine	508 (28.3)	94 (32.1)	414 (27.5)
Very low dose primaquine (<2mg/kg)	22 (1.2)	3 (1.0)	19 (1.3)
Low dose total primaquine (2-<5mg/kg)	196 (10.9)	31 (10.6)	165 (11.0)
High dose total primaquine (≥5mg/kg)	1,071 (59.6)	165 (56.3)	906 (60.2)
Primaquine daily dose categories			
No primaquine	508 (28.3)	94 (32.1)	414 (27.5)
Low dose (<0.375mg/kg/day)	185 (10.3)	29 (9.9)	156 (10.4)
Intermediate dose (0.375-<0.75mg/kg/day)	561 (31.2)	89 (30.4)	472 (31.4)
High dose (≥0.75mg/kg/day)	543 (30.2)	81 (27.6)	462 (30.7)
Primaquine duration			
7 days	520/1,289 (40.3)	72/199 (36.2)	448/1,090 (41.1)
14 days	769/1,289 (59.7)	127/199 (63.8)	642/1,090 (58.9)
Region			
Africa	426 (23.7)	84 (28.7)	342 (22.7)
Asia-Pacific	1,371 (76.3)	209 (71.3)	1,162 (77.3)
G6PD status (data from qualitative or quantitative test)			
Deficient (<30% activity)	1 (0.1)	0 (0.0)	1 (0.1)
Intermediate (≥30 & <70% activity)	18 (1.0)	3 (1.0)	15 (1.0)
Normal*	1,540 (85.7)	255 (87.0)	1,285 (85.4)
Unknown	238 (13.2)	35 (11.9)	203 (13.5)

CI – confidence interval; G6PD – glucose-6-phosphate dehydrogenase; PQ – primaquine; Data recorded as number (%), median (range), or n/N (%); 4 children aged less than 12 months are included in the dataset, of

these 3 received primaquine; * Patients were classified as normal if they were not identified as deficient on a qualitative test in some studies or if they had G6PD activity ≥70% on a quantitative test in other studies.

Table S21. Patients reporting at least one adverse event within 28 days of initiating primaquine (or equivalent time schedule in placebo / no primaquine groups) in patients with *P. vivax* or mixed malaria infection, by age category and primaquine dose

Primaquine group	All	<5years	5 to <15 years
Any	403/1,797 (22.4)	61/293 (20.8)	342/1,504 (22.7)
No primaquine	66/508 (13.0)	17/94 (18.1)	49/414 (11.8)
Low daily dose	36/185 (19.4)	3/29 (10.3)	23/156 (14.7)
Intermediate daily dose	144/561 (25.7)	21/89 (23.6)	123/472 (26.1)
High daily dose	167/543 (30.8)	20/81 (24.7)	147/462 (31.8)

n with adverse event/N evaluated (%) is shown; low dose – <0.375 mg/kg/day; intermediate dose – 0.375-<0.75 mg/kg/day; high dose - ≥0.75 mg/kg/day.

Table S22. Patients with any abdominal pain within 28 days of initiating primaquine (or equivalent time schedule in placebo / no primaquine groups) in patients with *P. vivax* or mixed malaria infection, by age category and primaquine dose

Primaquine group	All	<5years	5 to <15 years
Any	113/1,797 (6.3)	4/293 (1.4)	109/1,504 (7.3)
No primaquine	9/508 (1.8)	0/94 (0.0)	9/414 (2.2)
Low daily dose	3/185 (1.6)	0/29 (0.0)	3/156 (1.9)
Intermediate daily dose	38/561 (6.8)	1/89 (1.1)	37/472 (7.8)
High daily dose	63/543 (11.6)	3/81 (3.7)	60/462 (13.0)

n with adverse event/N evaluated (%) is shown; low dose – <0.375 mg/kg/day; intermediate dose – 0.375-<0.75 mg/kg/day; high dose - ≥0.75 mg/kg/day.

Table S23. Patients with any anaemia* within 28 days of initiating primaquine (or equivalent time schedule in placebo / no primaquine groups) in patients with *P. vivax* or mixed malaria infection, by age category and primaquine dose

Primaquine group	All	<5years	5 to <15 years
Any	43/1,797 (2.4)	10/293 (3.4)	33/1,504(2.2)
No primaquine	0/508 (0.0)	0/94 (0.0)	0/414 (0.0)
Low daily dose	2/185 (1.1)	0/29 (0.0)	2/156 (1.3)
Intermediate daily dose	23/561 (4.1)	6/89 (6.7)	17/472 (3.6)
High daily dose	18/543 (3.3)	4/81 (4.9)	14/462 (3.0)

n with adverse event/N evaluated (%) is shown; *Defined per study (See Table S2); low dose – <0.375 mg/kg/day; Intermediate dose – 0.375-<0.75 mg/kg/day; high dose - ≥0.75 mg/kg/day.

Table S24. Patients with any methaemoglobinaemia* reported within 28 days of initiating primaquine (or equivalent time schedule in placebo / no primaquine groups) in patients with *P. vivax* or mixed malaria infection, by age category and primaquine dose

Primaquine group	All	<5years	5 to <15 years
Any	11/1,797 (0.6)	1/293 (0.3)	10/1,504 (0.7)
No primaquine	0/508 (0.0)	0/94 (0.0)	0/414 (0.0)
Low daily dose	0/185 (0.0)	0/29 (0.0)	0/156 (0.0)
Intermediate daily dose	3/561 (0.5)	0/89 (0.0)	3/472 (0.6)
High daily dose	8/543 (1.5)	1/81 (1.2)	7/462 (1.5)

n with adverse event/N evaluated (%) is shown; *Includes MedDRA preferred term of cyanosis; low dose – <0.375 mg/kg/day; Intermediate dose – 0.375-<0.75 mg/kg/day; high dose - ≥0.75 mg/kg/day.

Table S25. Patients with any diarrhoea within 28 days of initiating primaquine (or equivalent time schedule in placebo / no primaquine groups) in patients with *P. vivax* or mixed malaria infection, by age category and primaquine dose

Primaquine group	All	<5years	5 to <15 years
Any	18/1,797 (1.0)	5/293 (1.7)	13/1,504 (0.9)
No primaquine	6/508 (1.2)	1/94 (1.1)	5/414 (1.2)
Low daily dose	1/185 (0.5)	1/29 (3.5)	0/156 (0.0)
Intermediate daily dose	4/561 (0.7)	1/89 (1.1)	3/472 (0.6)
High daily dose	7/543 (1.3)	2/81 (2.5)	5/462 (1.1)

n with adverse event/N evaluated (%) is shown; low dose – <0.375 mg/kg/day; Intermediate dose – 0.375-<0.75 mg/kg/day; high dose - ≥0.75 mg/kg/day.

Table S26. Patients with any anorexia within 28 days of initiating primaquine (or equivalent time schedule in placebo / no primaquine groups) in patients with *P. vivax* or mixed malaria infection, by age category and primaquine dose

Primaquine group	All	<5years	5 to <15 years
Any	21/1,797 (1.2)	4/293 (1.4)	17/1,504 (1.1)
No primaquine	4/508 (0.8)	1/94 (1.1)	3/414 (0.7)
Low daily dose	8/185 (4.3)	2/29 (6.9)	6/156 (3.9)
Intermediate daily dose	5/561 (0.9)	0/89 (0.0)	5/472 (1.1)
High daily dose	4/543 (0.7)	1/81 (1.2)	3/462 (0.7)

n with adverse event/N evaluated (%) is shown; low dose – <0.375 mg/kg/day; Intermediate dose – 0.375-<0.75 mg/kg/day; high dose - ≥0.75 mg/kg/day.

Table S27. Patients with any vomiting within 28 days of initiating primaquine (or equivalent time schedule in placebo / no primaquine groups) in patients with *P. vivax* or mixed malaria infection, by age category and primaquine dose

Primaquine group	All	<5years	5 to <15 years
Any	28/1,797 (1.6)	6/293 (2.1)	22/1,504 (1.5)
No primaquine	6/508 (1.2)	2/94 (2.1)	4/414 (1.0)
Low daily dose	2/185 (1.1)	0/29 (0.0)	2/156 (1.3)
Intermediate daily dose	8/561 (1.4)	2/89 (2.3)	6/472 (1.3)
High daily dose	12/543 (2.2)	2/81 (2.5)	10/462 (2.2)

n with adverse event/N evaluated (%) is shown; low dose – <0.375 mg/kg/day; Intermediate dose – 0.375-<0.75 mg/kg/day; high dose - ≥0.75 mg/kg/day.

Table S28: Description of serious adverse events considered by study investigator to be primaquine related (possibly, probably or definitely related)

Study	Country	Sch	Primaquine regimen	Age (yr)	Sex	G6PD Status	Description	Manage-ment	Criteria for SAE Report	Sever-ity*	Outcome	Causality#
**Haematological**												
Taylor 2019	Ethiopia	CQ	Intermediate daily dose Primaquine over 14 days	3	M	Screened as G6PD Normal (>=30%)	Haemolysis 3 days after starting PQ; Hb drop from 10.2 to 6.8/dL	Primaquine permanently discontinued, not hospitalised	Lab: Fall in Hb below 7g/dl	Severe	Full recovery	Probably related
Taylor 2019	Indonesia	DHP	High Daily dose Primaquine over 7 days	11	F	G6PD Normal (>30%). Subsequent Spectrophotometry G6PD activity =37%	Drop in Hb (haemolysis) to less than 7g/dL (from 11.6 to 6.9) on day 3 after starting primaquine	Temporary discontinuation of primaquine for 1 day, then completed course	Lab: Fall in Hb below 7g/dl	Moderate	Full recovery	Possibly related
Chu 2019	Thailand	DHP	High Daily dose Primaquine over 7 days	13	F	Screened G6PD Normal (>=30%). Subsequent Spectro activity = 62%"	Haemolysis 5 days after starting primaquine. Hb fell from 8.8 to 4.4g/dL	Primaquine ceased. Hospitalised for blood transfusion	Clinical: Blood transfusion	Severe	Full recovery	Probably related
Chu 2019	Thailand	CQ	Intermediate daily dose Primaquine over 14 days	13	M	Screened as G6PD Normal (>=30%)	Methaemoglobinaemia (and scrub typhus) on day 6 after starting primaquine	Hospitalised, primaquine ceased	Clinical: Hospitalised	Severe	Full recovery	Probably related
Taylor 2019	Indonesia	DHP	High Daily dose Primaquine over 7 days	11	F	Screened as G6PD Normal (>=30%)	Methaemoglobinaemia (symptomatic) and bronchopneumonia on day 10 after starting primaquine	Hospitalised, primaquine permanently ceased	Clinical: Hospitalised	Severe	Full recovery	Definitely related
Chu 2019	Thailand	DHP	Intermediate daily dose Primaquine over 14 days	14	M	Screened as G6PD Normal (>=30%)	Methaemoglobinaemia on day 12 after starting primaquine	Hospitalised for observation, primaquine ceased	Clinical (peri-oral cyanosis): Hospitalised	Moderate	Full recovery	Probably related
Chu 2019	Thailand	DHP	High Daily dose Primaquine over 7 days	4	F	Screened as G6PD Normal (>=30%)	Methaemoglobinaemia on day 5 after starting primaquine	Hospitalised for observation, primaquine ceased	Clinical (peri-oral cyanosis): Hospitalised	Moderate	Full recovery	Probably related

Chu 2019	Thailand	DHP	High Daily dose Primaquine over 7 days	14	M	Screened as G6PD Normal (>=30%)	Methaemoglobinaemia on day 5 after starting primaquine	Hospitalised for observation, primaquine ceased	Clinical (peri-oral cyanosis): Hospitalised	Modera te	Lost to follow up	Probably related
Chu 2019	Thailand	CQ	High Daily dose Primaquine over 7 days	5	M	Screened as G6PD Normal (>=30%)	Methaemoglobinaemia on day 5 after starting primaquine	Hospitalised for observation, primaquine ceased	Clinical (peri-oral cyanosis): Hospitalised	Modera te	Full recovery	Probably related
**Gastrointestinal SAEs**												
Taylor 2019	Ethiopia	CQ	High Daily dose Primaquine over 7 days	13	F	Screened as G6PD Normal (>=30%)	Severe persistent vomiting for 4 days from 2 days after starting primaquine	Hospitalised, primaquine temporarily ceased for 3 days, then completed full course	Clinical: Hospitalised	Severe	Full recovery	Possibly related
Taylor 2019	Indonesia	DHP	High Daily dose Primaquine over 7 days	9	F	Screened as G6PD Normal (>=30%)	Moderate dyspepsia (epigastric pain) 4 days after starting primaquine	Temporarily discontinued primaquine for 3 days	Clinical: Hospitalised	Modera te	Full recovery	Possibly related
Taylor 2019	Vietnam	CQ	High Daily dose Primaquine over 7 days	7	F	Screened as G6PD Normal (>=30%)	Moderate diarrhoea for 4 days from day 7 after starting PQ	Temporarily ceased primaquine for 3 days, then completed course	Clinical: Hospitalised	Modera te	Full recovery	Possibly related
**Hepatobiliary SAEs**												
Awab 2017	Afghanist an	CQ	Low daily Primaquine over 14 days	13	M	Screened as G6PD Normal (>=30%)	Jaundice and haemoglobinuria 2 days after starting primaquine	Primaquine stopped for two days to day 7, changed to weekly dose	Clinical: important medical event	Modera te	Full recovery	Possibly related
**Other Related SAEs**												
Chu 2019	Thailand	DHP	Intermediate daily dose Primaquine over 14 days	14	F	Screened as G6PD Normal (>=30%)	Pneumonia/oesophagitis on day 16 after starting primaquine	Hospitalised, IV antibiotics	Clinical: Hospitalised	Modera te	Full recovery	Possibly related

Awab 2017	Afghanistan	CQ	Low daily Primaquine over 14 days	13	F	Missing	Anaemia, tachycardia, lethargy and abdominal pain severe on day 7 after starting primaquine. Hb did not fall by ≥2 g/dL or to <7g/dL	Hospitalised on day 7 and withdrawn from study	Clinical: Hospitalised	Severe	Full recovery	Possibly related

Sch - Schizonticide; CQ - chloroquine, DHP - dihydroartemisinin-piperaquine; NA - not available; M - Male; F - Female; *Severity at highest when more than one event; # Causality defined according to categorisation by original study investigators.

References S1.

47. World Health Organization. WHO Child Growth Standards: Length/height-for-age, weight-for-age, weight-for-length, weight-for-height and body mass index-for-age: Methods and development. Geneva, 2006.
48. Battle KE, Lucas TCD, Nguyen M, et al. Mapping the global endemicity and clinical burden of Plasmodium vivax, 2000-17: a spatial and temporal modelling study. *Lancet* 2019.
49. Battle KE, Karhunen MS, Bhatt S, et al. Geographical variation in Plasmodium vivax relapse. *Malar J* 2014; **13**: 144.
50. Gaedigk A, Sangkuhl K, Whirl-Carrillo M, Klein T, Leeder JS. Prediction of CYP2D6 phenotype from genotype across world populations. *Genet Med* 2017; **19**(1): 69-76.
51. Gaedigk A, Simon SD, Pearce RE, Bradford LD, Kennedy MJ, Leeder JS. The CYP2D6 activity score: translating genotype information into a qualitative measure of phenotype. *Clin Pharmacol Ther* 2008; **83**(2): 234-42.
52. Caudle KE, Sangkuhl K, Whirl-Carrillo M, et al. Standardizing CYP2D6 Genotype to Phenotype Translation: Consensus Recommendations from the Clinical Pharmacogenetics Implementation Consortium and Dutch Pharmacogenetics Working Group. *Clin Transl Sci* 2020; **13**(1): 116-24.
53. Carmona-Fonseca J, Maestre A. Prevention of Plasmodium vivax malaria recurrence: efficacy of the standard total dose of primaquine administered over 3 days. *Acta Trop* 2009; **112**(2): 188-92.
54. Carmona-Fonseca J. Vivax malaria in children: Recurrences with standard total dose of primaquine administered in 3 vs. 7 days *Iatreia* 2010; **23**(1): 10-20.
55. Kheng S, Muth S, Taylor WR, et al. Tolerability and safety of weekly primaquine against relapse of Plasmodium vivax in Cambodians with glucose-6-phosphate dehydrogenase deficiency. *BMC Med* 2015; **13**: 203.
56. Valecha N, Savargaonkar D, Srivastava B, et al. Comparison of the safety and efficacy of fixed-dose combination of arterolane maleate and piperaquine phosphate with chloroquine in acute, uncomplicated Plasmodium vivax malaria: a phase III, multicentric, open-label study. *Malar J* 2016; **15**: 42.
57. Dharmawardena P, Rodrigo C, Mendis K, et al. Response of imported malaria patients to antimalarial medicines in Sri Lanka following malaria elimination. *PLoS One* 2017; **12**(11): e0188613.
58. Mekonnen DA, Abadura GS, Behaksra SW, et al. Treatment of uncomplicated Plasmodium vivax with chloroquine plus radical cure with primaquine without G6PDd testing is safe in Arba Minch, Ethiopia: assessment of clinical and parasitological response. *Malar J* 2023; **22**(1): 135.
59. Moore BR, Salman S, Tobe R, et al. Short-course, high-dose primaquine regimens for treatment of liver-stage vivax malaria in children. *Int J Infect Dis* 2023.
60. Arcelia F, Pasaribu AP, Yanni GN. Effectiveness of dihydroartemisinin-piperaquine after 10 years as treatment for vivax malaria in Indonesia. *J Infect Dev Ctries* 2023; **17**(5): 700-6.
61. Malla P, Wang Z, Brashear A, et al. Effectiveness of an Unsupervised Primaquine Regimen for Preventing Plasmodium vivax Malaria Relapses in Northeast Myanmar: A Single-Arm Nonrandomized Observational Study. *J Infect Dis* 2024; **229**(5): 1557-64.
62. Liu H, Xu JW, Deng DW, et al. Artemisinin-naphthoquine plus lower-dose primaquine to treat and prevent recurrence of Plasmodium vivax malaria: an open-label randomized and non-inferiority trial. *Parasit Vectors* 2024; **17**(1): 28.

63. Gebrie H, Yimer M, Ayehu A, et al. Efficacy and safety of chloroquine plus primaquine for the treatment of Plasmodium vivax malaria in Hamusit site, Northwestern Ethiopia. *Malar J* 2024; **23**(1): 202.
64. Manh ND, Thanh NV, Quang HH, et al. Therapeutic efficacy of pyronaridine-artesunate (Pyramax) in treating Plasmodium vivax malaria in the central highlands of Vietnam. *Antimicrob Agents Chemother* 2024: e0004424.
65. Villalobos-Salcedo JM, Tada MS, Kimura E, Menezes MJ, Pereira da Silva LH. In-vivo sensitivity of Plasmodium vivax isolates from Rond nia (western Amazon region, Brazil) to regimens including chloroquine and primaquine. *Ann Trop Med Parasitol* 2000; **94**(8): 749-58.
66. Eibach D, Ceron N, Krishnalall K, et al. Therapeutic efficacy of artemether-lumefantrine for Plasmodium vivax infections in a prospective study in Guyana. *Malar J* 2012; **11**: 347.
67. Leslie T, Rab MA, Ahmadzai H, et al. Compliance with 14-day primaquine therapy for radical cure of vivax malaria--a randomized placebo-controlled trial comparing unsupervised with supervised treatment. *Trans R Soc Trop Med Hyg* 2004; **98**(3): 168-73.
68. Orjuela-Sanchez P, da Silva NS, da Silva-Nunes M, Ferreira MU. Recurrent parasitemias and population dynamics of Plasmodium vivax polymorphisms in rural Amazonia. *Am J Trop Med Hyg* 2009; **81**(6): 961-8.
69. Van den Eede P, Soto-Calle VE, Delgado C, et al. Plasmodium vivax sub-patent infections after radical treatment are common in Peruvian patients: results of a 1-year prospective cohort study. *PLoS One* 2011; **6**(1): e16257.
70. Betuela I, Rosanas-Urgell A, Kiniboro B, et al. Relapses contribute significantly to the risk of Plasmodium vivax infection and disease in Papua New Guinean children 1-5 years of age. *J Infect Dis* 2012; **206**(11): 1771-80.
71. Negreiros S, Farias S, Viana GM, et al. Efficacy of Chloroquine and Primaquine for the Treatment of Uncomplicated Plasmodium vivax Malaria in Cruzeiro do Sul, Brazil. *Am J Trop Med Hyg* 2016; **95**(5): 1061-8.
72. Pham TV, Nguyen HV, Aguirre AR, et al. Plasmodium vivax morbidity after radical cure: A cohort study in Central Vietnam. *PLoS Med* 2019; **16**(5): e1002784.
73. Abdon NP, Pinto AY, das Silva Rdo S, de Souza JM. [Assessment of the response to reduced treatment schemes for vivax malaria]. *Rev Soc Bras Med Trop* 2001; **34**(4): 343-8.
74. Buchachart K, Krudsood S, Singhasivanon P, et al. Effect of primaquine standard dose (15 mg/day for 14 days) in the treatment of vivax malaria patients in Thailand. *Southeast Asian J Trop Med Public Health* 2001; **32**(4): 720-6.
75. Solari Soto L, Soto Tarazona AR, Mendoza Requena D, Llanos Cuentas EA. Ensayo clinico del tratamiento de la malaria vivax con esquema acortado de primaquina comparado con el esquema tradicional. *Rev Soc Peru Med Interna* 2002; **15**(4): 197-9.
76. Yadav RS, Ghosh SK. Radical curative efficacy of five-day regimen of primaquine for treatment of Plasmodium vivax malaria in India. *J Parasitol* 2002; **88**(5): 1042-4.
77. Pinto AY, Azevedo CH, da Silva JB, de Souza JM. Assessment of chloroquine single dose treatment of malaria due to Plasmodium vivax in Brazilian Amazon. *Rev Inst Med Trop Sao Paulo* 2003; **45**(6): 327-31.
78. Maguire JD, Krisin, Marwoto H, Richie TL, Fryauff DJ, Baird JK. Mefloquine is highly efficacious against chloroquine-resistant Plasmodium vivax malaria and Plasmodium falciparum malaria in Papua, Indonesia. *Clin Infect Dis* 2006; **42**(8): 1067-72.
79. Krudsood S, Tangpukdee N, Wilairatana P, et al. High-dose primaquine regimens against relapse of Plasmodium vivax malaria. *Am J Trop Med Hyg* 2008; **78**(5): 736-40.

80. Nateghpour M, Edrissian G, Torabi A, et al. Monitoring of Plasmodium vivax and Plasmodium falciparum response to chloroquine in Bandar-Abbas district, Hormozgan province, Iran. [Arabic]. *Tehran Uni Med J* 2009; **67**(3): 178-83.
81. Takeuchi R, Lawpoolsri S, Imwong M, et al. Directly-observed therapy (DOT) for the radical 14-day primaquine treatment of Plasmodium vivax malaria on the Thai-Myanmar border. *Malar J* 2010; **9**: 308.
82. Maneeboonyang W, Lawpoolsri S, Puangsa-Art S, et al. Directly observed therapy with primaquine to reduce the recurrence rate of plasmodium vivax infection along the Thai-Myanmar border. *Southeast Asian J Trop Med Public Health* 2011; **42**(1): 9-18.
83. Graf PC, Durand S, Alvarez Antonio C, et al. Failure of Supervised Chloroquine and Primaquine Regimen for the Treatment of Plasmodium vivax in the Peruvian Amazon. *Malar Res Treat* 2012; **2012**: 936067.
84. Pedro RS, Guaraldo L, Campos DP, Costa AP, Daniel-Ribeiro CT, Brasil P. Plasmodium vivax malaria relapses at a travel medicine centre in Rio de Janeiro, a non-endemic area in Brazil. *Malar J* 2012; **11**: 245.
85. Ganguly S, Saha P, Guha SK, et al. In vivo therapeutic efficacy of chloroquine alone or in combination with primaquine against vivax malaria in Kolkata, West Bengal, India, and polymorphism in pvmdr1 and pvcrt-o genes. *Antimicrob Agents Chemother* 2013; **57**(3): 1246-51.
86. Liu H, Yang HL, Xu JW, Wang JZ, Nie RH, Li CF. Artemisinin-naphthoquine combination versus chloroquine-primaquine to treat vivax malaria: an open-label randomized and non-inferiority trial in Yunnan Province, China. *Malar J* 2013; **12**: 409.
87. Miahipour A, Keshavarz H, Heidari A, Raeisi A, Rezaeian M, Rezaei S. Assessment of the efficacy of 8 weeks of primaquine for the prevention of relapse in vivax malaria patients using SSCP-PCR and sequencing in South and South-East Iran, 2008-2011. *Trans R Soc Trop Med Hyg* 2013; **107**(7): 420-6.
88. Zhu G, Lu F, Cao J, et al. Blood stage of Plasmodium vivax in central China is still susceptible to chloroquine plus primaquine combination therapy. *Am J Trop Med Hyg* 2013; **89**(1): 184-7.
89. Delgado-Ratto C, Soto-Calle VE, Van den Eede P, et al. Population structure and spatio-temporal transmission dynamics of Plasmodium vivax after radical cure treatment in a rural village of the Peruvian Amazon. *Malar J* 2014; **13**: 8.
90. Mac Donald-Ottevanger MS, Adhin MR, Jitan JK, Bretas G, Vreden SG. Primaquine double dose for 7 days is inferior to single-dose treatment for 14 days in preventing Plasmodium vivax recurrent episodes in Suriname. *Infect Drug Resist* 2018; **11**: 3-8.
91. Azarian Moghadam HN, Raeisi M, Motevalli Haghi A, Edrissian A, Farivar G. Monitoring the Response of *Plasmodium vivax* to Chloroquine and Uncomplicated *P. falciparum* to Artesunate-fansidar Antimalarials in Southeastern Iran. *Iran J Parasitol* 2018; **13**(1): 31-8.
92. Han KT, Lin K, Han ZY, et al. Efficacy and Safety of Pyronaridine-Artesunate for the Treatment of Uncomplicated Plasmodium falciparum and Plasmodium vivax Malaria in Myanmar. *Am J Trop Med Hyg* 2020; **103**(3): 1088-93.
93. Shaikh S, Chohan MN, Touseef M, Buriro HWA, Shah MA, Muzaffar S. Safety of Weekly Primaquine in Glucose 6 Phosphatase Dehydrogenase (G6PD) Deficient Children. *J Pharm Res Int* 2021; **33**(57B): 452-8.
